# GeneWalk identifies relevant gene functions for a biological context using network representation learning

**DOI:** 10.1186/s13059-021-02264-8

**Published:** 2021-02-02

**Authors:** Robert Ietswaart, Benjamin M. Gyori, John A. Bachman, Peter K. Sorger, L. Stirling Churchman

**Affiliations:** 1 Department of Genetics, Blavatnik Institute, Harvard Medical School, Boston, MA 02115 USA; 2 Laboratory of Systems Pharmacology, Harvard Medical School, Boston, MA 02115 USA

**Keywords:** GeneWalk, Functional analysis, Differential expression, Machine learning, Network representation learning, INDRA (Integrated Network and Dynamical Reasoning Assembler), Pathway Commons, GO enrichment, Gene set enrichment analysis, Next-generation sequencing, RNA-seq, NET-seq

## Abstract

A bottleneck in high-throughput functional genomics experiments is identifying the most important genes and their relevant functions from a list of gene hits. Gene Ontology (GO) enrichment methods provide insight at the gene set level. Here, we introduce GeneWalk (github.com/churchmanlab/genewalk) that identifies individual genes and their relevant functions critical for the experimental setting under examination. After the automatic assembly of an experiment-specific gene regulatory network, GeneWalk uses representation learning to quantify the similarity between vector representations of each gene and its GO annotations, yielding annotation significance scores that reflect the experimental context. By performing gene- and condition-specific functional analysis, GeneWalk converts a list of genes into data-driven hypotheses.

**Supplementary Information:**

The online version contains supplementary material available at 10.1186/s13059-021-02264-8.

## Background

High-throughput functional genomics experiments generate genome-scale datasets that require computational analyses [1–5], which yield lists of “hit” genes [2]. Such lists typically include hundreds to thousands of genes of interest, ranked by *p* values, whose biological significance (as opposed to technical validity) is not always clear [6]. The main bottleneck is in determining which genes, and which specific functions of those genes, are most relevant to the biological context of the experiment. Many genes have slightly different functions that depend on its context, such as cell type or stress response (e.g., *EGFR*, which affects transcription, signal transduction, cell division, survival, motility, and other processes [7]). At the extreme, some genes encode so-called moonlighting proteins that serve vastly different functions [8]. Thus, the challenge lies in prioritizing those genes worthy of further study and identifying their most pertinent functions for the particular biological context. For example with EGFR, identifying actin filament binding and cell division activities as being higher relevance than signal transduction and kinase activities would inform hypotheses that help prioritize downstream experiments: altering the EGFR actin-binding domain and testing for cell division phenotypes would be given more precedence than inhibiting the EGFR kinase activity. In this way, gene-specific knowledge provides data-driven, mechanistic hypotheses that are experimentally testable and help accelerate the biological discovery process.

Gene Ontology (GO) annotations are commonly used to add information related to biological processes, molecular functions, and cellular components to genes and gene lists [7], but they list all of a gene’s functions across many biological contexts. GO and gene set enrichment analysis (GSEA) are used to reveal which biological processes are enriched, i.e., globally relevant, under each specific condition [1, 3, 4, 9–16]. Gene sets are functionally annotated collections of genes, while pathways are described by gene networks (graphs) consisting of a set of genes as the vertices (nodes) and their interactions (e.g., activation, repression, or phosphorylation) as edges [14, 16–21]. A network structure (topology) provides additional biological information that is leveraged by pathway analysis methods [14, 17, 20, 22]. Many GO functional analysis methods have been developed and assessed [1, 3, 5, 9–16, 19, 23–30], ranging from relatively simple yet powerful algorithms that perform gene set overrepresentation analyses [1, 9, 11–13, 16, 23], to more sophisticated GO or pathway topology-based [5, 10, 14, 22] or permutation-based (functional class scoring) methods that take genome-wide expression levels into account [3, 15]. These enrichment approaches are not designed to provide gene-specific functional information; nevertheless, the methods can be inverted by focusing only on globally enriched GO terms when considering an individual gene. However, this inversion procedure is typically inconvenient to perform for the end-user and underpowered because the relevant function of an individual gene is not necessarily globally relevant. For instance, the actin filament binding activity of EGFR might not be an enriched GO term and vice versa, a globally enriched unspecific GO term such as “protein binding” might not be relevant for all input genes with that annotation. Thus, researchers typically rely on expert knowledge gleaned from experience and literature searches to identify relevant gene functions. While effective, obtaining expertise is time consuming and is not possible for unexplored biological contexts. Accordingly, new methods are required to generate functional information about individual genes under particular conditions of interest or biological contexts. To address this need, we developed GeneWalk, a knowledge-based machine learning and statistical modeling method that highlights the gene functions that are relevant for a specific biological context.

GeneWalk takes advantage of two recent advances in computational biology [31, 32]: deep learning to condense information [33–36], and generation of gene networks derived from database aggregation efforts [14, 16, 18, 21, 37, 38]. Unsupervised representation learning by neural networks can reduce the dimensionality of complex datasets or networks [33, 34]. Thus, nodes in any network can be represented by vectors of low dimensionality defined based on the network topology [33–35, 39–41]. Networks of biological mechanisms are now available from knowledge bases [16, 38], such as Pathway Commons [18, 42], STRING [16], OmniPath [38], and the Integrated Network and Dynamical Reasoning Assembler (INDRA [21, 37]). INDRA reaction statements (e.g., protein phosphorylation, transcriptional regulation, or biological process regulation) are extracted from the body of biomedical literature using either natural language processing systems of primary literature in the minable NCBI corpus or queries on pathway databases.

**Fig. 1:**
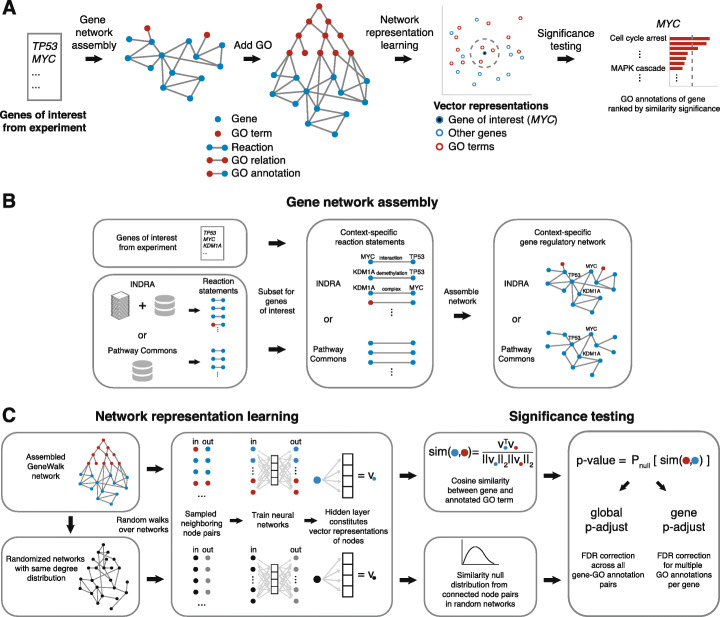
GeneWalk methodology. **a** Schematic introducing the key aspects of the GeneWalk method. The input is a list with genes of interest, e.g., all differentially expressed genes under a certain experimental condition. Using the INDRA [21, 37] or Pathway Commons [18, 43] knowledge base, all molecular reactions in which these genes are involved are retrieved and assembled in a condition-specific gene regulatory network, to which GO ontology and annotations are then connected. Through network representation learning, each gene and GO term can be represented as vectors, suitable for similarity significance testing. For each gene, GeneWalk gives as output the similarities with all of the connected GO terms, along with their significance, specifying which annotated functions are relevant under the experimental condition. **b** Schematic details of the gene network assembly procedure from the input list with genes of interest and knowledge base INDRA or Pathway Commons (PC). These knowledge bases provide reaction statements. INDRA accumulates these from automated literature reading and database queries [21, 37], while Pathway Common only queries databases [18, 43]. Another difference between INDRA and PC is that INDRA also provides gene–GO relations through automated text mining. Then a strict subset results in the collection of context-specific reaction statements that involve only genes of interest. These reaction statements are then assembled into a gene regulatory network. **c** Schematic details of the network representation learning and significance testing parts of GeneWalk. Random walks are generated from the assembled GeneWalk Network (GWN), yielding a large collection of sampled neighboring node pairs, which form the training set of (input,output) of a fully connected shallow neural network (NN) where each node from the GWN is represented as a single feature. The learned hidden layer is the vector representation of a node. And the similarity of a node pair then equals the cosine similarity between the corresponding node vectors. To enable similarity significance testing, we generated randomized networks that were also subjected to DeepWalk and whose resulting cosine similarity values form the null distributions used to calculate a *p* value of the experimental similarities between a gene and GO term node. Finally, because multiple GO terms were tested, we applied two FDR corrections that address different questions. The gene *p*-adjust values rank the context-specific relevance of all annotated GO terms for a pre-defined gene of interest. The global *p*-adjust can be used to identify relevant genes and their functions across the whole input gene list

GeneWalk is developed to generate functional relevance information about individual genes in a biological context under study. GeneWalk first automatically assembles a biological network from a knowledge base and the GO ontology starting with a list of genes of interest (e.g., differentially expressed genes or hits from a genetic screen) as input (Fig. 1a). The network structure is learned through random walks using an unsupervised network representation learning algorithm (DeepWalk [33]). The resultant vector representations enable a quantitative comparison between genes and GO terms, highlighting the GO terms most relevant for the biological context under study. As output, GeneWalk provides for each input gene its direct GO annotations ranked by their statistical relevance. We demonstrate the applicability of GeneWalk by using it to analyze three experiments in which the data were obtained by either RNA-seq or native elongating transcript sequencing (NET-seq), which probes the nascent transcriptome. GeneWalk identified context-relevant GO terms while filtering out the majority of irrelevant GO terms for each gene, allowing the researcher to quickly hone in on relevant targets. Thus, GeneWalk serves as a rapid data-driven hypothesis-generating tool for exploratory functional gene analysis.

## Results

### Assembly of a context-specific GeneWalk network

The first step in GeneWalk is assembly of a network that describes the relationships between genes and GO terms, starting with a list of relevant genes obtained from a specific experimental assay (Fig. 1a). These genes could be differentially expressed (DE) between some condition (such as a genetic mutation or drug treatment) and a control experiment, or the results of a high-throughput genetic screen. GeneWalk can run with any number of input genes*,* but the context generally becomes better defined in the presence of many (> 10) input genes (see the “Methods” section for details). A context-specific gene network (Fig. 1a, b) is then assembled using a knowledge base such as INDRA [21, 37]. Collections of INDRA statements involving at least two different differentially expressed (DE) genes or a DE gene and GO term are assembled into a gene network such that each gene is represented as a node and each statement as an edge (Fig. 1b). For comparison, we also generated a context-specific gene network using Pathway Commons [18, 43], which generally resulted in fewer gene–gene connections and no (INDRA-originating) gene–GO connections [18, 43] (Fig. 1b). This gene network, either from INDRA or PC, is then appended to a GO network [4] in which edges represent ontological relationships between GO terms as nodes (Fig. 1a). To further connect genes to GO terms in the network, we add edges between genes and their annotated GO terms (Fig. 1a), resulting in a full GeneWalk network (GWN).

### Network representation learning with random walks

To determine how genes and GO terms that constitute GWN nodes relate to one another, we perform random walks in the network. A network representation learning algorithm (DeepWalk [33]) transforms the random walks into descriptions of how the nodes are embedded in the network, yielding vector representations for each node (Fig. 1c). Specifically, short random walks sample the local neighborhood of all nodes, providing a collection of neighboring node pairs, which in turn form a training set of input–output pairs for a fully connected neural network (NN) with one hidden layer (Fig. 1c). Each input and output GWN node from each sampled pair are one-hot encoded to form respectively the input and output to the NN during training. So, this NN learns which output GWN nodes have been sampled for a given input GWN node. After training, the resultant hidden layer weights form the vector representation of any (one-hot encoded) GWN input node (Fig. 1c, see the “Methods” section for further details). In this way, groups of interconnected genes and GO terms that are mechanistically or functionally linked to each other occur most frequently as sampled gene–GO term pairs, which can be scored by the cosine similarity between their NN-derived vector representations (Fig. 1c).

### Gene–GO term similarity significance testing

Next, GeneWalk calculates whether the cosine similarity values between a gene and GO terms are higher than expected by chance using a significance test (Fig. 1c). A null distribution of similarity values between node vectors is generated using representation learning on networks with randomly permuted edges (Additional file 1: Supplementary Fig. S1A). Comparisons with the null distribution yield *p* values for all experimental gene–GO term pairs (Fig. 1c). These *p* values are then corrected for multiple GO annotation testing using the Benjamini-Hochberg false discovery rate (FDR), either across all gene–GO term pairs yielding a global adjusted *p* value (global *p*-adjust), or across all GO annotations per gene (gene p-adjust). To decrease variability arising from stochastic walk sampling, network representation learning and significance testing are repeated 10 times to generate the mean and 95% confidence intervals of the p-adjust estimates as the final outputs. The gene *p*-adjust values rank the context-specific relevance of all annotated GO terms for a pre-defined gene of interest. The global *p*-adjust can be used to identify relevant genes and their functions across the whole input gene list. For both global and gene *p*-adjust, an FDR threshold can then be set to classify all annotated GO terms that have a high cosine similarity with this gene in a statistically significant manner. We term these GO terms as “relevant” to the gene for this biological context defined by the experimental input gene set. Gene function significance arises through a high degree of interconnections with other functionally related genes in the GWN. So genes with many relevant functions are likely central to the specific biological context and thus are prime candidates for further investigation.

### Identification of ground truth benchmark datasets for testing GeneWalk

To test GeneWalk and compare its predictions, we set out to identify ground truth benchmark datasets where the relevant subset of GO annotations of individual genes are known for the specific biological context. However, as far as we could determine, no such dataset exists. Existing gene function prediction benchmarks [44] were not suitable to serve as a ground truth for this learning task due to the lack of context-specificity. We considered comparing GeneWalk predictions using simulated data. However, this approach might not adequately reflect reality and would suffer from human bias, since an in silico ground truth would be constructed from chosen first principles. We recognized that GeneWalk’s task is similar to what researchers with expert knowledge do when considering a list of genes. They use their expertise to identify which GO annotations for each gene are the most relevant for the experimental context they investigate. So to test GeneWalk, we applied it to two experimental contexts in which phenotypes and molecular mechanisms are already well characterized. We unbiasedly text-mined the primary publications that first described the experimental contexts to identify the genes and their functions that were deemed relevant according to the expertise of the authors. In this manner, we generated two ground truth datasets that enable systematic and unbiased performance assessment of GeneWalk and other functional analysis approaches on the task of identifying the relevant GO terms for each gene of interest in a particular biological context.

### GeneWalk application to brain myelination RNA-seq data

**Fig. 2:**
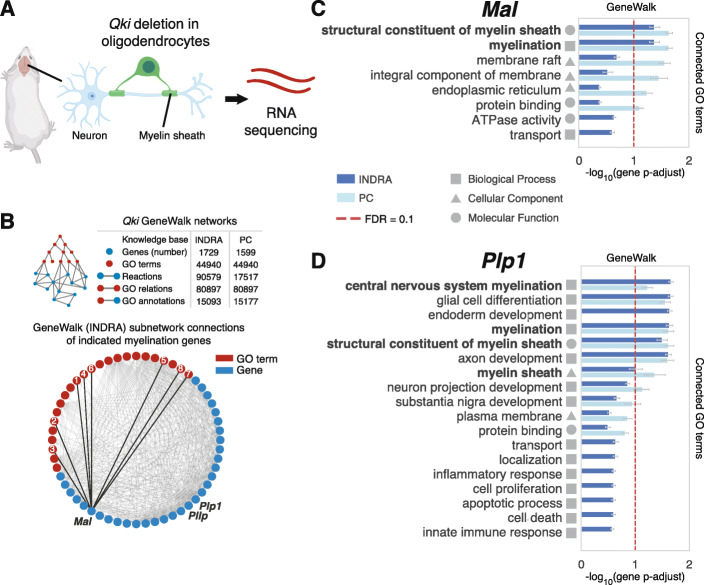
GeneWalk identifies myelination functions from mouse brain RNA-seq. **a** Schematic of the experimental design in Darbelli et al. [45]. Deletion of *Qki*, a gene that encodes RNA-binding proteins, in oligodendrocytes results in hypomyelination in the mouse brain. RNA-seq was performed on *Qki*-deficient and control mice (each three biological replicates). **b** Schematic with statistics of the *qki* GeneWalk networks (GWNs) using either INDRA or Pathway Commons (PC) as a knowledge base. Also shown is a visualization of the INDRA GWN subnetwork of myelination-related genes *Mal*, *PllP*, and *Plp1*, all their connected genes and GO terms. Edges (gray) connecting node pairs indicate the presence of INDRA reaction statements or GO annotations between the two respective nodes. Edges between *Mal* and its GO connections (numbered according to rank order in **c**) are highlighted (bold). **c** GeneWalk results for *Mal* in the *qki* condition using either INDRA or Pathway Commons (PC) as a knowledge base source to assemble the GeneWalk network. All GO terms connected to *Mal* are rank-ordered by Benjamini-Hochberg FDR-adjusted *p*-value (*p*-adjust), indicating their functional relevance to *Mal* in the context of *Qki* deletion in oligodendrocytes. Error bars indicate 95% confidence intervals of gene *p*-adjust. FDR = 0.1 (dashed red line) and domains of GO annotations (square: biological process, triangle: cellular component, and circle: molecular function) are also shown. Additional file 1 shows full GeneWalk results using the INDRA or PC knowledge base. **d** As in **c** for *Plp1*

In the brain (Fig. 2a), neurons are myelinated in a *Qki*-dependent manner by oligodendrocytes [45, 46]. The *Qki* gene encodes an RNA binding protein involved in alternative splicing [45, 46], and conditional *Qki* deletion in mouse oligodendrocytes (Fig. 2a) results in severe hypomyelination and death of the animal [46]. Analysis of RNA-seq comparing animals with *Qki-*deficient and *Qki*-proficient oligodendrocytes [45] revealed 1899 DE genes (Additional file 1: Supplementary Fig. S1B).

We initiated GeneWalk with 1861 unique Mouse Gene Database (MGD) identifiers [47] corresponding to the DE gene set (Additional File 1: Supplementary Fig. S1B), of which 94% (1750) mapped to different human orthologs using INDRA’s integrated mouse-to-human gene mappings [47, 48]. INDRA statements were retrieved for 83% of the genes, of which the vast majority (82% of the initial 1861) had at least one connected GO term (Fig. 2b). We first investigated *Myelin and lymphocyte protein (Mal)*, *Plasmolipin (Pllp)*, and *Proteolipid protein 1* (*Plp1)*: the three most strongly downregulated genes (Additional file 1: Supplementary Fig. S1B) that had been previously characterized as essential for myelination [49–52]. GeneWalk determined that annotated GO terms related to myelination were most relevant to these DE genes *Mal*, *Plp1*, and *Pllp* (Fig. 2c, d, Additional file 1: Supplementary Fig. S1C), verifying that GeneWalk can identify GO terms for each of these genes that are pertinent for the biological context.

To investigate the algorithm’s general applicability, we also performed a GeneWalk analysis using Pathway Commons (PC), which provided 5-fold fewer reaction statements (Fig. 2b, Additional file 1: Supplementary Fig. S1D) compared to the INDRA knowledge base. INDRA also provides gene–GO term connections obtained from the literature, for example *Plp1* and “inflammatory response” (Fig. 2d, Additional file 2), while GeneWalk with PC utilizes GO annotations provided by the GO consortium only (Additional file 2). Nevertheless, the ordering of GO term significance for these myelination genes was similar regardless of whether PC or INDRA was used to generate the GWN (Fig. 2c, d, Additional file 1: Supplementary Fig. S1C), demonstrating that GeneWalk is robust to differences in the underlying knowledge base and the amount of available molecular information.

### Performance comparison on *qki* ground truth between GeneWalk and alternative functional analysis methods

**Fig. 3:**
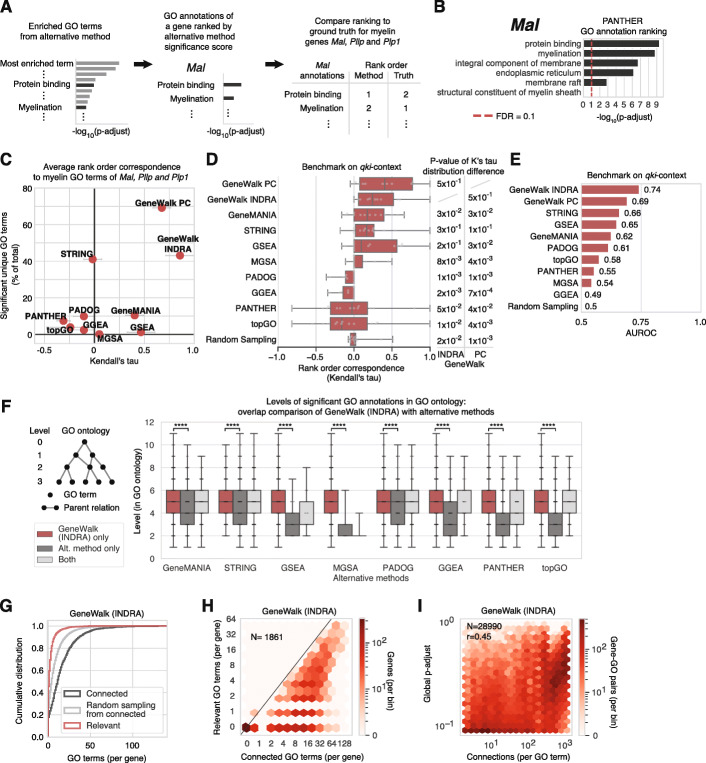
Systematic comparison of GeneWalk with alternative methods and model robustness analysis. **a** Schematic of systematic procedure to compare alternative methods with GeneWalk. The alternative methods (see Table 1 for brief descriptions and “Methods” section for details) are mostly based on a form of GO enrichment analysis, and result in a list of (globally) overrepresented GO terms with a significance value (*p*-adjust). For individual genes, such as *Mal*, we select the GO terms that are also direct annotations of that gene and form a GO annotation relevance rank order based on the method’s significance levels. Lastly for myelin-related genes *Mal*, *Pllp*, *and Plp1*, we compare the results of GeneWalk (gene *p*-adjust) and all other methods to the same ground truth ranking which is myelin terms shared 1st and all other annotations shared 2nd using Kendall’s tau to assess the rank order correspondence with the ground truth. **b** Example of GO annotation relevance ranking for *Mal* with the procedure outlined in (**a**) with alternative method PANTHER. **c** Results of systematic comparison outlined in (**a**), with average Kendall’s tau values (*x*-axis) over the three myelin genes. Error bars indicate standard error on the mean. The *y*-axis indicates the number of different unique GO annotations that are significant (for GeneWalk global p-adjust and for alternative methods *p*-adjust at FDR = 0.1) as a percentage of all unique GO annotation terms across all *qki* DE genes present in the GWN. **d** Distribution of Kendall’s tau rank order correspondences of predictions from GeneWalk and alternative methods (Table 1) to the ground truth benchmark of the *qki-*context where all gene GO annotations pairs mentioned by Darbelli et al. in [45] are jointly top-ranked and all other gene–GO annotations pairs are jointly bottom ranked. All methods are ordered by the median of their Kendall’s tau distribution, indicating their relative performances. Statistical differences between GeneWalk (INDRA or PC) and other methods are determined with the Wilcoxon signed-rank sum test. See Methods for details. **e** Bar chart of the area under receiver operating characteristic (AUROC) performance metric for GeneWalk and alternative methods (Table 1) on the benchmark described in (**e**) when considered as a binary classification task: identifying gene-function pairs as relevant or not. **f** Boxplots of the GO term levels of all significant (for GeneWalk global p-adjust and for alternative methods p-adjust at FDR = 0.1) gene–GO annotation pairs across all *qki* DE genes present in the GWN. A higher GO level reflects more specific concept information in the GO ontology [7]. Direct overlap comparison of GeneWalk (with INDRA) with the rankings from alternative methods is indicated with individual data points shown. For comparison of GeneWalk (with PC), see Additional file 1: Supplementary Fig. S1F. A Mann-Whitney *U* test indicates the statistical differences in median levels between levels significant for only GeneWalk as compared to only the alternative method, ****p < 10^−4^. **g** Cumulative distribution of number of connected (black) and relevant (red) GO terms per gene, alongside a simulation that uniformly randomly sampled from the number of connected terms (gray) for GWNs with INDRA. The number of relevant GO terms was smaller than with randomly sampling connections (KS test: *p* < 1e−16). **h** Hexagon density plot for all genes of interest (*N* = 1861) in terms of number of connected GO terms and number of relevant GO terms (at FDR = 0.1) resulting from the *Qki-*deficient condition GeneWalk using INDRA as a knowledge base. **i** Hexagon density plot of all tested gene–GO pairs (*N* = 28,990) as a function of GO term connectivity and similarity significance (global *p*-adjust, Pearson correlation *r* = 0.45) for the GWN described in (**h**)

Most analyses of functional genomics data use gene set-based analyses to identify enriched GO terms, but they are not designed for the end-user to easily retrieve gene-specific information. To illustrate with PANTHER GO enrichment analysis, we find that *Mal* is absent from the gene sets corresponding to the most highly enriched biological process GO terms and only first appears as part of “ensheathment of neurons” (108 genes) and “myelination” (106 genes), the 15th and 17th term when ranked by fold enrichment (Additional file 1: Supplementary Fig. S1E), and 63rd and 70th when ranked by *p*-adjust from its Fisher’s exact test. Nevertheless, we systematically compared GeneWalk against eight alternative methods [1, 3, 5, 10, 13–16] (Table 1) in their ability to rank-order myelin-related GO terms above all other direct GO annotations for the three myelin genes, *Mal*, *Plp1*, and *Pllp*, as an initial ground truth benchmark task (Fig. 3a, see the “Methods” section for details). The ground truth rank order for these three genes was a tied rank 1 for all GO annotations that contained the string “myelin” and thus considered relevant, and a tied rank 2 for all other GO annotations that were labeled as not relevant. For fair comparison, GO annotation versions and evidence codes used by the alternative methods were matched to those of GeneWalk, as much as their publicly available software implementations allowed these specifications (see Additional file 1: Supplementary methods for details). The alternative methods yield a set of enriched GO terms with a statistical significance score that depends on the method (e.g., the *p*-adjust values for PANTHER). For each gene, GO annotations are sorted by their significance scores and compared to the ground truth ranking (Fig. 3a) by Kendall’s tau rank order correspondence. For example, GO term “structural constituent of myelin sheath” is relevant specifically for *Mal* according to GeneWalk (Fig. 2c), but it is not enriched across the whole input gene set with PANTHER (Fig. 3b). Conversely, “protein binding” is an enriched GO term with PANTHER and also a GO annotation of *Mal* (Fig. 3b), but it is not related to myelin and thus contributes negatively to PANTHER’s Kendall’s tau rank order score (Fig. 3c). For this initial benchmark test of top ranking myelin GO annotations for the three myelin genes, GeneWalk outperformed all alternative methods (Fig. 3c).

**Table 1:** Overview of GeneWalk and alternative methods used for systematic comparison of gene function relevance scoring. The alternative methods were selected based on prevalence of usage or characteristic model features

		Requirements			
Method	Objective	Input type	GO annotations	Gene network	Defining model characteristic
**GeneWalk**	Gene function relevance scoring	Gene list	Yes	Yes	Network representation learning (cosine similarity)
**PANTHER**	Gene set enrichment	Gene list	Yes	No	Overrepresentation analysis (Fisher Exact test)
**GeneMANIA**	Gene function prediction (binary classification)	Gene list	Yes	Yes	Network label propagation algorithm
**GGEA**	Gene set enrichment	Quantitative expression score for all genes	Yes	Yes	Gene set overrepresentation analysis accounting for gene network
**GSEA**	Gene set enrichment	Quantitative expression score for all genes	Yes	No	Gene set enrichment analysis (permutation score test)
**MGSA**	Gene set relevance scoring	Gene list	Yes	No	Bayesian network (posterior probability)
**PADOG**	Gene set enrichment	Expression levels for all genes	Yes	No	Pathway Analysis with Down-weighting of Overlapping Genes (permutation score test)
**STRING**	Gene set enrichment	Gene list	Yes	No	Overrepresentation analysis (Hypergeometric test)
**topGO**	Gene set enrichment	Gene list	Yes	No	Overrepresentation analysis (Fisher Exact test) with decorrelation of parental GO terms

To compare the methods further, we extended our benchmark performance analysis to a larger set of genes. We unbiasedly defined the *qki*-context ground truth from [45], the primary publication that describes the *Qki*-deletion RNA-seq experiment and the gene regulation relevant to the hypomyelination phenotype of the mouse. Through systematic, manual text mining, we tabulated all gene-biological term pairs (Additional file 3) mentioned in the same text sentences or figures from the publication [45]. Then for each gene, its GO annotations that contained the biological terms were classified as “relevant” and assigned a tied rank order 1, and the remaining annotations as “not relevant” and assigned tied rank order 2. We cannot rule out that additional genes and functions are in truth relevant, but not mentioned in publication [45]. However, our conservative methodology does capture those considered relevant enough to be mentioned by the authors, given their expert-level knowledge of the *qki*-context [45]. This systematic procedure resulted in 29 different listed genes (Additional file 3). Fourteen of them were DE and had at least one GO annotation that contained the corresponding biological term, which cumulated into the unbiased ground truth benchmark data set of 37 relevant gene–GO annotation pairs and 100 not-relevant pairs (Additional file 3).

On the task of ranking the relevant GO annotations higher than not-relevant annotations across all genes present in this ground truth benchmark, GeneWalk (with PC and INDRA) had the highest median rank order correspondences compared to the alternative methods (Fig. 3d). Most of the Kendall’s tau distribution differences were also statistically significant (Fig. 3d, Wilcoxon paired-rank sum test). Moreover, we compared the methods through a binary classification task (gene GO annotation pairs are relevant or not-relevant), through the metric area under receiver operating characteristic (AUROC, see the “Methods” section for details). The AUROC is determined using the quantitative significance score -log_10_(p-adj), but it remains a less comprehensive metric than the Kendall’s tau, since it does not consider the relative GO annotation ranking order per gene. GeneWalk (AUROC = 0.74 and 0.69 for INDRA and PC respectively) performed better than all other methods and (AUROCs < 0.67) random selection (AUROC = 0.5, Fig. 3e, Additional file 1: Supplementary Fig. S1F, see the “Methods” section for details). The GeneWalk (INDRA) network contains 3 edges (out of 186569, Fig. 2b) that originate from the ground truth publication through INDRA’s automated text mining [21, 37]. Removal of these edges from the GWN reduces its benchmark performance only marginally and all our conclusions on the comparison between GeneWalk and other methods remain unaltered (Additional File 1: Supplementary Fig. S1G).

Enrichment-based methods also provide significance values for GO terms that are transitively connected to a gene’s direct GO annotations through at least one parental relation in the GO ontology. Extending the ground truth positives to include GO terms that are parentally related to a relevant direct GO annotation does not make a difference to our results (Additional file 1: Fig. S1H), because these additional GO terms are not direct GO annotations and thus do not contribute to the ranking. When we “parentally enhanced” the methods by propagating significant *p*-adjust values from any such parent GO terms down to any direct GO annotation that was not called as significant, our results remained again unaffected (Additional file 1: Fig. S1I). This demonstrates that, even when considering enriched parental GO terms, enrichment-based methods do not provide the same gene-specific information as GeneWalk.

Compared to the alternative methods, GeneWalk identified more unique GO terms for all input genes (Fig. 3c). All the alternative methods, except GeneMANIA [5], seek to find a limited number of GO terms that are relevant across *all* members of the corresponding input gene set (Table 1). In contrast, GeneWalk’s objective is to identify GO terms relevant to *individual* genes by sampling its connectivity with direct GO annotations, explaining why more unique GO terms are found (Fig. 3c). Consistently, across all input genes, GeneWalk finds GO terms that are more specific in terms of concept generality compared to the other methods (Fig. 3f, Additional file 1: Supplementary Fig. S1J-L), which we quantified via each GO term’s level in the ontology [7] (Fig. 3f). We conclude that GeneWalk ranks the known molecular functions of myelin and other genes relevant to the *qki-*context systematically better than all tested alternative functional analysis methods and provides more detailed gene function information across the input gene set.

### Systematic GeneWalk model robustness analysis

To understand the robustness of GeneWalk performances, we assessed several model assumptions. First, we found that GeneWalk is selective by focusing on the statistically relevant genes and their functions as the total number of relevant GO terms was smaller than expected by chance (KS test, *p* < 10^−16^ for both INDRA and PC derived GWNs; Fig. 3g, Additional file 1: Supplementary Fig. S2A). Fifty-four percent (1011) of the DE genes in the GWN had at least one relevant GO term (global *p*-adjust < 0.1, Additional file 2). Second, despite the fact that the GeneWalk algorithm contains stochastic procedures, its output predictions are reproducible between replicate runs: no statistically significant differences were observed between the global *p*-adjust values of a gene–GO connection pair when GeneWalk was independently run twice and compared through a two-tailed *t*-test with Benjamini-Hochberg multiple testing correction (with FDR = 0.01). Third, GeneWalk performance relies on the GO ontology and gene–gene interactions in the GWN (Additional file 1: Supplementary Fig. S2, see the “Methods” section): the exclusion of either of these features weakened or abolished the ability to top rank the relevance of myelin terms for *Mal*, *Pllp*, and *Plp1* (Additional file 1: Supplementary Fig. S2B). Furthermore, it resulted in a much reduced correlation with the default GeneWalk model across all gene–GO annotation similarity and global *p*-adjust values (Additional file 1: Supplementary Fig. S2C). Fourth, GeneWalk is context-specific: the use of all expressed genes in the genome as input substantially alters predictions (Additional file 1: Supplementary Fig. S2C). Fifth, GeneWalk does not use the GO ontology transitivity property directly: performance deterioration resulted from inclusion of direct edges between transitive gene–GO relations (Additional file 1: Supplementary Fig. S2B,C). Sixth, GeneWalk performance is robust against repeating DeepWalk 3 times instead of 10 times, or the inclusion of all input DE genes, instead of only those connected through direct gene–gene edges. These modifications had little effect on all model performances (Additional file 1: Supplementary Fig. S2B,C,D), with only minor stochastic variation between replicates (Additional file 1: Supplementary Fig. S2C,D). Seventh, GeneWalk is fairly robust against variations of the network representation learning technique: the use of biased random walks through node2vec [34] or DeepWalk [33] with very long random walks did not improve and slightly reduced their respective GeneWalk performances (Additional file 1: Supplementary Fig. S2B,C). DeepWalk with infinitely long walks is mathematically equivalent to a matrix factorization approach that generates low-dimensional vector representations through spectral decomposition [53]. So GeneWalk, which employs DeepWalk with short random walks, remains preferred to these two alternative network embedding approaches. Finally, GeneWalk’s similarity null distribution randomization scheme is robust against variations: randomization of only the gene–gene and gene–GO connections instead of all GWN edges did not substantially affect the performance or resulting similarity null distribution (Additional file 1: Supplementary Fig. S2B,C). All these conclusions were reconfirmed in our rank order correspondence task applied to a second ground truth case study detailed in the next sections (Additional file 1: Supplementary Fig. S2E,F). Overall, GeneWalk utilizes the network structure of all its data sources: the gene–gene interactions, gene–GO annotations, and the GO ontology in a robust and reproducible manner with limited stochastic variation.

### GeneWalk determines function relevance independent of the degree of annotation

Genes are annotated with different numbers of GO terms. To determine whether GeneWalk is biased with respect to the number of connected GO terms per gene node (the annotation degree), we compared the number of significant GO terms to node degree. The annotation degree is known to introduce a bias into enrichment analyses based on the Fisher exact test, which overestimates significance for GO terms with large annotated gene sets [13]. We found that with GeneWalk the distribution of relevant GO terms was relatively uniform for all DE genes (Fig. 3h, Additional file 1: Supplementary Fig. S3A, Likelihood Ratio test, *χ*^2^ test *p* value = 1 for both INDRA and PC), showing that there was no correlation between the numbers of connected and similar GO terms. When we considered only gene–GO term connections originating from INDRA through its automated literature reading functionality, as opposed to GO annotation, we also observed a dispersed distribution (Additional file 1: Supplementary Fig. S3B), although it was not completely uniform (Likelihood Ratio test, χ^2^-test p-value < 10^−16^). The results show that GeneWalk does not suffer from many biases in significance testing towards genes with high or low degrees of annotation.

We also asked whether a GO term with high connectivity is more likely to exhibit strong similarity to a gene simply because it is a highly connected node in the GWN. We found that this was not the case in general (Fig. 3i), although there was a weak correlation between the number of connections for a GO term and GeneWalk global *p*-adjust values (Pearson correlation coefficient *r* = 0.45). This effect could mostly be explained by a few highly connected GO terms (Additional file 1: Supplementary Fig. S3C), e.g., “cell proliferation” (1152 connections), “apoptotic process” (967 connections), or “localization” (536 connections), for which INDRA detects many genetic associations reported in the literature. However, these GO terms reflect high-level biological concepts that are rarely the specific function of an individual gene. Indeed, in the Pathway Commons-derived GWN, which only contains GO annotations, these GO terms have far fewer connections (42, 33, and 12, respectively), and the correlation between connectivity and similarity significance was lower (*r* = 0.26; Additional file 1: Supplementary Fig. S3D). Therefore, we conclude that GeneWalk controls for concept generality in GO term relevance ranking and does not suffer from substantial biases related to the degree of GO term connectivity.

### Generation of gene-specific functions and systematic hypotheses for *Plxnb3* using GeneWalk

GeneWalk helps generate gene-specific mechanistic hypotheses. *Plxnb3* was one of the most strongly downregulated genes upon *Qki* deletion (Fig. S1B). GeneWalk revealed that more than half of its connected GO terms were relevant (gene *p*-adjust < 0.1), suggesting that *Plxnb3* is a priority candidate with many of its annotated functions affected by the *Qki* deletion (Additional file 1: Supplementary Fig. S3E). *Plxnb3* is expressed in oligodendrocytes specifically [54], but it is not annotated to be involved in myelination or related to *Qki* (Additional file 1: Supplementary Fig. S3E, Additional file 2). Furthermore, a PubMed search of *Plxnb3* with the query terms “myelination” or “*Qki*” yielded no results. The most relevant functions of *Plxnb3* were “cell–cell adhesion mediator activity,” “semaphorin receptor complex,” “regulation of GTPase activity,” “cell chemotaxis,” and “semaphorin receptor activity” (Additional file 1: Supplementary Fig. S3E), raising the possibility that *Plxnb3* could contribute to the myelination process through one of these activities. This procedure illustrates how GeneWalk can be utilized in combination with differential expression strength to predict gene-specific functions and hypotheses in a systematic manner.

### Nascent transcriptome response to bromodomain inhibitor JQ1 using human NET-seq

**Fig. 4:**
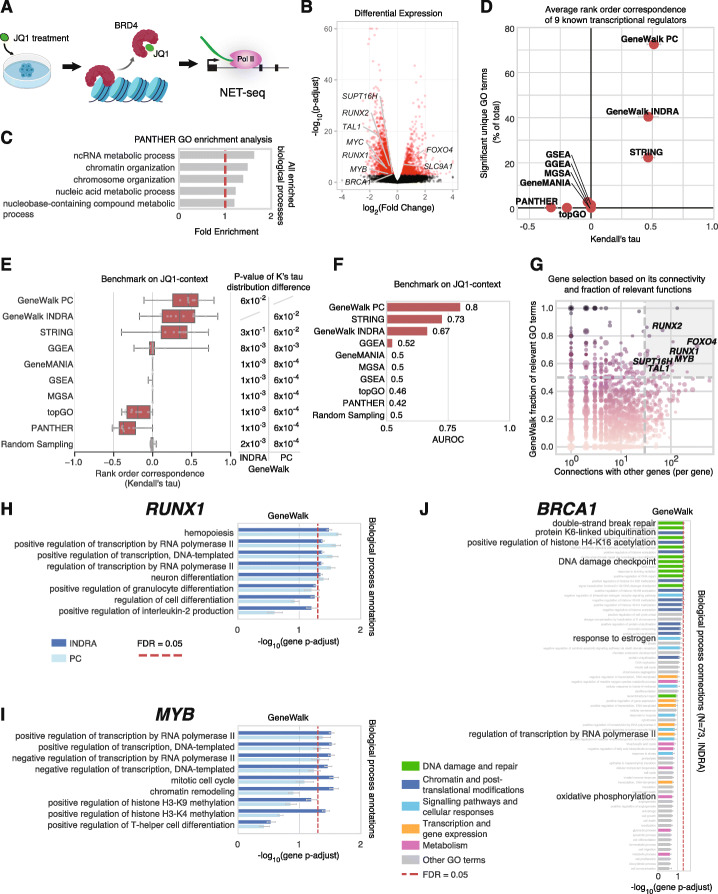
GeneWalk analysis of nascent transcriptome response to BRD4 inhibition in T-ALL cells. **a** Schematic of the experimental design in Winter et al. [55]. NET-seq was performed on JQ1-treated MOLT4 cells (1 μM for 2 h, alongside DMSO controls, two biological replicates each). JQ1 targets BRD4 and other BET bromodomain family members, causing BRD4 to dissociate from chromatin [55]. **b** Volcano plot showing the results of a differential expression (DE) analysis comparing RNA Polymerase II gene coverage between JQ1 and DMSO control samples. DE genes (*N* = 2692), indicated in red, were used as an input to GeneWalk. All other genes are depicted in black. **c** All enriched Biological Process GO terms (five enriched terms, Fisher exact test, FDR = 0.05) in JQ1 condition, ranked by fold enrichment, obtained by GO enrichment analysis using PANTHER [1]. Red line indicates a fold enrichment value of 1, indicating the background. **d** The number of different unique GO annotations (*y*-axis) that are significant (*p*-adjust < 0.1) as a percentage of all unique GO annotation terms across all JQ1 DE genes present in the GWN. Average Kendall’s tau rank order correspondences of predictions from GeneWalk and alternative methods (*x*-axis) over previously identified transcriptional regulators that are part of the JQ1-context (Additional file 3) [55, 56] *MYC*, *MYB*, *RUNX1*, *RUNX2*, *TAL1*, *SATB1*, *ERG*, *ETV6*, and *TCF12*. Error bars indicate standard error on the mean. **e** Distribution of Kendall’s tau rank order correspondences of predictions from GeneWalk and seven tested alternative methods (Table 1) to the ground truth benchmark of the JQ1*-*context where all gene GO annotations pairs mentioned in [55–57] are jointly top-ranked and all other gene–GO annotations pairs are jointly bottom ranked. All methods are ordered by the median of their Kendall’s tau distribution, indicating their relative performances. Statistical differences between GeneWalk (INDRA or PC) and other methods are determined with the Wilcoxon signed-rank sum test. See the “Methods” for details. **f** Bar chart of the area under receiver operating characteristic (AUROC) performance metric for GeneWalk and alternative methods (Table 1) on the benchmark described in (**e**) when considered as a binary classification task: identifying gene-function pairs as relevant or not. **g** Scatter plot with DE genes as data points showing the GeneWalk fraction of relevant GO terms over total number of connected GO terms (min_f, minimum value between INDRA and PC GWNs) as a function of the number of gene connections in the GWN (N^gene^, again minimal value between INDRA and PC). The circle size scales with the differential expression significance strength (−log_10_(*p*-adjust)) and the color hue with min_f. Twenty genes were identified with min_f > 0.5 and *N*^gene^ > 30 (gray-shaded area, see Table 2 for complete list). **h** GeneWalk results for the transcriptional regulator *RUNX1* under JQ1 treatment. Annotated biological process terms are rank-ordered by gene FDR adjusted *p* value. Error bars indicate 95% confidence intervals of gene *p*-adjust. FDR = 0.05 (dashed red line) is also shown. See Additional file 1 for full details. **i** As in (**h**) for transcriptional regulator *MYB.* **j** As in (**h**) for transcriptional regulator *BRCA1*. INDRA annotations are indicated by class: DNA damage and repair (green), chromatin, and post-translational modifications (dark blue), signaling pathways and cellular responses (light blue), transcription and gene expression (yellow), metabolism (purple), and other GO terms (gray)

To test GeneWalk on another well-characterized model system, we reanalyzed published NET-seq data [55] describing the response of a human T-cell acute lymphoblastic leukemia (T-ALL) cell line to treatment with JQ1 (Fig. 4a), a small molecule that targets the BET bromodomain in BRD4 and other BET family members [58]. NET-seq measures RNA polymerase position genome-wide at single-nucleotide resolution [55, 59], yielding a quantitative description of the nascent transcriptome. JQ1 treatment resulted in large genome-wide transcriptional changes [55, 58]. We calculated Pol II coverage per gene and identified differentially transcribed protein-coding genes using DEseq2 [2] (Fig. 4b). INDRA statements were retrieved for 82% of DE genes (*N* = 2670), 79% of which had connected GO terms. GeneWalk identified relevant GO terms for 48% of DE genes (global *p*-adjust < 0.1, Additional file 2), similar to the statistics for the mouse brain RNA-seq data.

### Systematic comparison of GeneWalk with alternative functional analysis methods using JQ1 ground truth

PANTHER GO enrichment analysis of the JQ1 DE gene set only yielded five high-level (generic) functions such as “ncRNA metabolic process” and “chromatin organization” with low fold enrichment (range, 1.2–1.7; Fig. 4c, Fisher’s exact test, FDR = 0.05). One alternative functional analysis method, PADOG [15], was not included because it requires as input at least three replicates and the JQ1 experiment consisted of two biological replicates per treatment [55]. Thus, we benchmarked GeneWalk and the remaining seven alternatives (Table 1) to our JQ1 context. In comparison to the seven tested alternative methods (Fig. 4d), GeneWalk identified even more unique relevant GO terms than in the application to the *qki* study (Fig. 3c). To compare the relevance identification performance of GeneWalk against alternative methods, we generated an unbiased JQ1-context ground truth data set through the systematic text mining procedure as described for the *qki*-context benchmark analysis. We extracted all gene-biological term pairs mentioned in Winter et al. [55], the primary publication that described the JQ1 NET-seq experiment, as well as the abstracts from Sanda et al. [56] and Sharma et al. [57], that altogether characterized the JQ1-context in T-ALL cells: a total of 88 relevant and 196 not-relevant gene–GO annotation pairs, from 14 different DE genes (Additional file 3). The relevance rank order correspondence test for JQ1 indicated that GeneWalk with PC outperformed all the other methods when ranked by the median of the Kendall’s tau distributions (Fig. 4e), while GeneWalk with INDRA performed on par with STRING and better than the rest. With binary classification (Fig. 4f, S3F), GeneWalk (PC) performed best (AUROC = 0.80), STRING came second (AUROC = 0.73), and GeneWalk (INDRA) ranking third (AUROC = 0.67). The other methods had AUROC values around the baseline value of 0.5 (Fig. 4f, S3F), due to their lack of significant results. Removal of the 10 GeneWalk (INDRA) network edges originating from the JQ1 ground truth publications, extending the ground truth with indirect GO annotations, or “parentally enhancing” methods with enriched indirect GO annotations did not affect the above conclusions as the results remained largely unaltered (Additional File 1: Supplementary Fig. S3G-I). The performances over the combination of *qki* and JQ1 benchmark data (Additional file 1: Supplementary Fig. S3J-L) reconfirm the conclusion that GeneWalk overall performs better than the alternative methods on the tasks on ranking (Additional file 1: Supplementary Fig. S3J) and binary classification of relevant GO annotations (Additional file 1: Supplementary Fig. S3K-L). We conclude that these results reveal the limitations of GO enrichment analysis when many functionally unrelated genes are misregulated. GeneWalk does not suffer from this limitation, because it is based on the local regulatory network connectivity with other treatment-affected genes.

### GeneWalk identifies known transcriptional regulators responding to JQ1 treatment

To test whether we could identify any previously identified transcriptional regulator genes that were affected by JQ1 treatment, we focused on genes with a high fraction of relevant GO terms over all connected terms according to GeneWalk with both INDRA and Pathway Commons knowledge bases (Fig. 4g, fraction > 0.5). We reasoned that by further selecting for genes with a large connectivity with other DE genes (Fig. 4g, gene connectivity > 30), we might identify candidate genes that mediate the observed transcriptional changes. With this procedure, we identified 21 genes (Fig. 4g, Table 2), of which 14 (Fisher Exact test, odds ratio = 13, *p* = 3 × 10^−8^) had relevant transcription-related annotations (Table 2). When also including gene–GO term relations obtained through the literature with INDRA, this number rose to 17 (Fisher’s exact test, odds ratio = 11 *p* = 7 × 10^−7^, Table 1). Among these were *RUNX1* (Fig. 4h), *MYB* (Fig. 4i), and *TAL1*, 3 out of 8 DE genes (Fisher Exact test, odds ratio = 93, *p* = 2 × 10^−5^) that have previously been identified as part of a core transcriptional circuitry important to our leukemia model system [55, 56]. The other 5 DE genes with transcription-related GO annotations in this reported core circuitry are [55, 56] *MYC*, *SATB1*, *ERG*, *ERV6*, and *TCF12* (Additional file 3)*.* Additionally, *RUNX2*, a previously reported transcriptional regulator of T-ALL [57], was also identified by GeneWalk (Fig. 4g). All other core circuitry components previously reported in [55, 56] were either not DE and thus not part of the input gene list or did not have any transcription-related GO annotations (Additional file 3). For this test set of 9 previously identified transcriptional regulators, GeneWalk systematically ranks transcription-related GO terms as most relevant according to Kendall’s tau rank order correspondence (Fig. 4d). Lastly, GeneWalk also found newly implicated genes (Fig. 4g) such as *SUPT16H* (Additional file 1: Supplementary Fig. S4A), with its most relevant cellular component term being “FACT complex” (gene p-adjust = 0.01, Additional file 2), as expected, and *FOXO4* (Additional file 1: Supplementary Fig. S4B) with relevant molecular functions such as “RNA polymerase II transcription factor activity, sequence-specific DNA binding” (gene p-adjust = 0.03, Additional file 2). These results demonstrate the capability of GeneWalk to systematically identify genes with relevant transcription-related functions in the context of the JQ1 response.

**Table 2:** GeneWalk identifies transcriptional regulators among highly connected genes with many significant functions in the JQ1 condition

Gene (ranked by connectivity with other genes)	Most relevant biological process annotation (GeneWalk with Pathway Commons knowledge base)	Gene has any significant transcription-related annotations (FDR = 0.125)?
*FOXO4*	Positive regulation of transcription by RNA polymerase II	Yes
*CTNNB1*	Canonical Wnt signaling pathway involved in negative regulation of apoptotic process	Yes
*MYB*	Positive regulation of transcription by RNA polymerase II	Yes
*RUNX1*	Hemopoiesis	Yes
*GABPB2*	Positive regulation of transcription by RNA polymerase II	Yes
*CDKN1A*	DNA damage response, signal transduction by p53 class mediator resulting in cell cycle arrest	INDRA only
*PPARG*	Response to lipid	Yes
*TFAP4*	Positive regulation of transcription	Yes
*VCL*	Platelet aggregation	No
*TFDP2*	Positive regulation of transcription by RNA polymerase II	Yes
*RUNX2*	Hemopoiesis	Yes
*CDC5L*	Positive regulation of transcription by RNA polymerase II	Yes
*DICER1*	Conversion of ds siRNA to ss siRNA involved in RNA interference	No
*RREB1*	Positive regulation of transcription by RNA polymerase II	Yes
*TAL1*	Positive regulation of transcription by RNA polymerase II	Yes
*MRE11*	Double-strand break repair via nonhomologous end joining	No
*ELOVL6*	Fatty acid elongation, saturated fatty acid	INDRA only
*EPAS1*	Cellular response to hypoxia	Yes
*EDNRA*	Artery smooth muscle contraction	INDRA only
*SUPT16H*	DNA replication-independent nucleosome organization	Yes
*HIST2H2AC*	Chromatin organization	No

### GeneWalk quantitatively ranks GO annotation relevance for genes with many functions

Many genes are involved in a large variety of different processes that frequently occur through the encoded-protein serving moonlighting functions in different cellular, environmental, or biological contexts [8]. These genes will have a large number of GO annotations that might not all be relevant in a particular context. GeneWalk is well suited to identify the relevant functions for genes encoding moonlighting proteins. To look at genes serving a specific role after JQ1 treatment, we identified 20 DE genes with at least 40 connected GO terms, of which no more than 50% were relevant (Additional file 1: Supplementary Fig. S4C, Additional file 4). Among them were *EGFR*, a gene with many established functions discussed above, and *MYC*, a widely studied proto-oncogene and member of the reported T-ALL core transcriptional circuitry [55]. This explains why *MYC* was not identified with our transcriptional regulator analysis (Fig. 4g): the majority of *MYC* annotations, especially those unrelated to transcription, were insignificant in the JQ1 condition (Additional file 2). *BRCA1* was another downregulated gene (Fig. 4b, Additional file 1: Supplementary Fig. S4C,D) with 23% (17) of its 73 connected biological processes being relevant (Fig. 4j, FDR = 0.05, Additional file 2). GeneWalk ranked DNA damage and repair-related processes as most relevant (Fig. 4j, gene p-adjust < 0.05), followed by histone and other post-translational modification-related terms (gene p-adjust = 0.05–0.07). Transcription, metabolism, and other GO terms were the least relevant (gene *p*-adjust > 0.09). These results demonstrate the capability of GeneWalk to systematically prioritize context-specific functions over less plausible alternatives, which is especially useful when considering genes encoding moonlighting proteins.

### GeneWalk investigation of cellular response to isoginkgetin

**Fig. 5:**
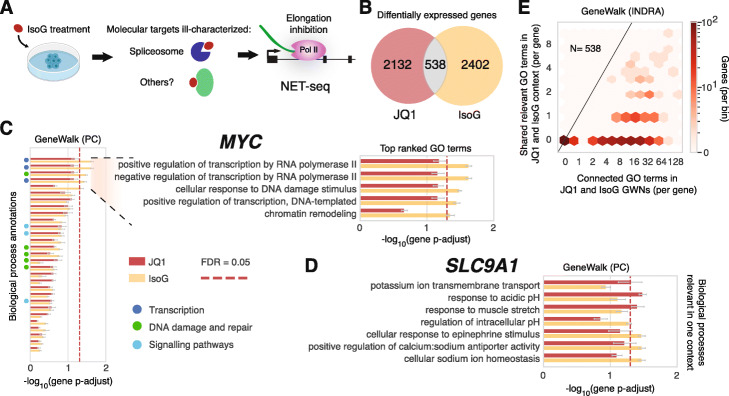
GeneWalk determines condition-specific functions through comparison of nascent transcriptome response to IsoG and JQ1 treatment. **a** Schematic of the experimental design in Boswell et al. [61]. NET-seq was performed on isoginkgetin (IsoG)-treated HeLa S3 cells (30 μM IsoG for 6 h, alongside DMSO controls, two biological replicates each). The in vivo molecular targets remain incompletely characterized, as IsoG treatment causes widespread Pol II elongation inhibition. **b** Venn diagram detailing the overlap (Fisher’s exact test: *p* = 0.02, odds ratio = 1.1, 95% confidence interval [1.0, 1.3]) of DE genes between JQ1 and IsoG treatments as described in Fig. 4b and S5A. **c** GeneWalk results (with PC as data source) for *MYC* in the JQ1 (red) and IsoG (yellow) condition. Annotated biological processes are rank-ordered by FDR-adjusted *p* value, indicating the relative functional importance of transcription (dark blue), DNA damage and repair (green), and signaling pathways (light blue) to *MYC* under the IsoG condition. The top five most relevant GO terms are described in the insets. See Additional file 2 for full details. Red dashed line indicates FDR = 0.05*.* **d** As in **c** for *SLC9A1*, showing the biological process terms that are relevant in either JQ1 or IsoG condition. **e** Hexagon density plot for overlapping DE genes (*N* = 538) in terms of number of overlapping relevant GO terms (FDR = 0.1) and number of possible shared connected GO terms for the GeneWalk network using INDRA as a knowledge base

To investigate the context-specificity of GeneWalk model predictions, we compared the transcriptional responses induced by JQ1 to those with the biflavonoid isoginkgetin (IsoG), a plant natural product and putative anti-tumor compound whose mechanism of action remains unknown. IsoG inhibits pre-mRNA splicing in vitro and in vivo [60] and also causes widespread accumulation of PoI II at the 5′ ends of genes, indicating an additional role as a Pol II elongation inhibitor [61]. Through DE analysis of NET-seq data obtained from HeLa S3 cells treated with IsoG (Fig. 5a), we identified a total of 2940 genes as differentially transcribed, most of which exhibited upregulated Pol II occupancy (Additional file 1: Supplementary Fig. S5A, FDR = 0.001). Using INDRA and Pathway Commons as the knowledge bases, we applied GeneWalk to these DE genes and found that 18% had at least one relevant GO term (FDR = 0.1, Additional file 2).

To identify candidate genes that could be involved in the IsoG-mediated transcriptional response, we searched for genes that were both strongly differentially expressed (p-adjust < 10^−25^) and had a large fraction of functions significantly affected according to the GeneWalk analyses with both INDRA and Pathway Commons (Additional file 1: Supplementary Fig. S5B, fraction > 0.8). In this manner, we identified three genes: *HES1*, *EGR1*, and *IRF1* (Additional file 1: Supplementary Fig. S5B)*. HES1* had “negative regulation of transcription, DNA templated” as one of the most relevant biological processes (Additional file 1: Supplementary Fig. S5C) and has been reported to inhibit transcription elongation [62]. *EGR1* and *IRF1* both had as most relevant term “positive regulation of transcription by RNA polymerase II” (Additional file 1: Supplementary Fig. S5D,E).

### Comparison between JQ1 and IsoG analyses indicates that GeneWalk yields condition-specific gene functions

To confirm that GeneWalk’s function assignments are not constant and depend on the experimental condition, we compared GeneWalk analyses of JQ1 and IsoG treatments. Between the JQ1 and IsoG condition, 538 DE genes were shared (Fig. 5b), marginally more than expected by chance (Fisher’s exact test: *p* = 0.02, odds ratio = 1.1, 95% confidence interval [1.0, 1.3]). As examples, we compared the overlap of relevant GO terms of *MYC* and *SLC9A1*, which are common DE genes between JQ1 (Fig. 4b) and IsoG treatment (Additional file 1: Supplementary Fig. S5A). *MYC* is annotated to be involved in 29 biological processes (Fig. 5c). Between the two GeneWalk analyses, *MYC* showed 5 significant biological processes and 9 molecular functions for IsoG and 0 and 1 respectively for JQ1 (Fig. 5c, Additional file 1: Supplementary Fig. S6A, FDR = 0.05). “Nucleus” and “nucleoplasm” were significant cellular components in both conditions (Additional file 1: Supplementary Fig. S6B). For *SLC9A1*, different biological processes were significant for each condition. For example, *SLC9A1* had “potassium ion transmembrane transport” and “response to acidic pH” as relevant only for the JQ1-context and “cellular sodium ion homeostasis” specific to IsoG treatment (Fig. 5d, FDR = 0.05). Thus, despite the common technical aspects such as organism under study and sequencing assay type, GeneWalk is capable of selecting which functions are specifically relevant for each experimental condition.

Overall, the numbers of shared relevant GO terms determined by GeneWalk were relatively uniformly distributed (Fig. 5e, Additional file 1: Supplementary Fig. S6C, Likelihood Ratio test, *χ*^2^-test *p* value = 1 for both INDRA and PC), indicating a lack of systematic bias in function assignment. Many genes had no shared terms between the two drug treatments (Fig. 5e), suggesting that those DE genes have different roles in each condition. We found similar results for GO terms originating from INDRA (Additional file 1: Supplementary Fig. S6D, Likelihood Ratio test, *χ*^2^ test *p* value = 1). We conclude that GeneWalk is able to determine context-specific functions as a consequence of differences in the context-specific gene–gene interactions part of the GeneWalk network.

## Discussion

Here we have described GeneWalk, a machine learning and statistical modeling method that identifies condition-specific functions of individual genes. Although we demonstrate its capabilities with differentially expressed genes obtained by two experimental approaches, RNA-seq and NET-seq, GeneWalk is capable of analyzing gene hit lists arising from many other types of experimental assays, such as CRISPR screens or mass spectrometry. In principle, for any gene of interest it is possible to recover relevant information by manual searches of the scientific literature. However, manual searching is time consuming when dozens or more genes are involved and potentially biased, because manual searches are typically incomplete. In contrast, GeneWalk provides a principled way to score gene–GO annotation associations based on systematic assembly of prior knowledge curated from the scientific literature. Information about context-specific gene functions can lead to hypotheses about gene regulation even when transcriptome-wide enrichment methods fail to yield significant results. If no GO annotations and molecular information on a gene have been reported, GeneWalk cannot make any functional relevance prediction. However, this bias towards studied genes is clearly also present for manual searches or enrichment analyses. Currently, only connected GO terms are considered for identification of function relevance, but we imagine that GeneWalk could be extended to predict novel gene functions because of high similarity scores between a gene and unconnected GO terms.

The GeneWalk applications in this study used the INDRA [21, 37] and Pathway Commons [18] knowledge bases which enable automated assembly of a GeneWalk network. Although these databases are optimized for human genes, we show that when mouse genes can be mapped unambiguously to their human orthologues, a network can still be assembled. For more distant species, this approach is likely to be insufficient. Nevertheless, GeneWalk should be readily applicable in other model organisms, such as yeast, given the availability of annotated gene regulatory networks, from knowledge bases such as STRING [16] and OmniPath [38], and GeneWalk’s option to analyze user-provided pre-assembled networks.

GeneWalk’s model architecture and hyperparameters are generally robust enough to accommodate user-provided input gene lists of various sizes. We have shown that three key components of the GWN are essential for GeneWalk: the GO ontology, GO annotations, and gene–gene connections that specify the biological context. GeneWalk only ranks a gene’s direct GO annotations as defined by the GO consortium [7], who generally assign the most biologically specific GO term as an annotation. Through this design, GeneWalk avoids redundancy, because parentally related indirect GO terms sometimes contain highly similar biological information. It also ensures the highest level of GO annotation specificity for individual genes. Generic terms such as “biological process” would otherwise also be considered as an indirect GO annotation of each gene. Inclusion of indirect GO annotations was tested and indeed showed a deteriorated benchmark performance (Additional file 1: Supplementary Fig. S2).

We demonstrated the use of DeepWalk for our network representation learning task, but matrix factorization or diffusion-based methods can also generate network embeddings and are applied to various biological problems [53, 63–69]. For some deep learning methods that use gene expression as input, the vector representation (latent space) dimensionality can affect the granularity of biological conclusions [70]. This is unlikely to be the case with GeneWalk for several reasons. GeneWalk does not model gene expression data, but instead a GWN network consisting of nodes and edges. Its network representation learning through DeepWalk relies only on the cosine similarity, a scalar derived from the vector representations without using any latent feature values directly. Furthermore, the latent space dimensionality (dim_rep = 8) is optimized to maximize the variance of the resulting gene–GO term cosine similarity distribution (see the “Methods” section for details), which is essentially a measure of information captured and optimum in the model’s bias-variance tradeoff. This cosine similarity distribution is dependent on the size and complexity of the GWN in terms of nodes and edges. Since there are more than 47,000 GO terms and typically at most ~ 3000 input genes, the number of nodes in the GWN is to first order determined by the GO ontology and thus constant. The network edges are originating from the GO ontology, gene–GO annotations and gene–gene interaction edges. We have shown that both for INDRA and PC, which have varying numbers of gene–gene edges, the results are consistent and correlations between replicate runs remain strong (Fig. S2). So to first order, the GWN network size and complexity is largely constant so adjustments of the vector dimensionality are not necessary. For significantly less complex organisms or very small input gene lists (order of 10 input genes), the number of gene–gene edges in the GWN might be far lower and sparsely distributed. In those cases, a smaller latent space dimensionality could be explored to optimally encode the GWN network structure.

## Conclusions

GeneWalk identifies relevant gene functions for a biological context under study. All existing knowledge on a user-provided gene list is assembled into a GeneWalk network that describes the context. Network representation learning together with statistical significance testing then enables systematic prioritization of relevant genes and their functions. We provide GeneWalk as a general open source tool (github.com/churchmanlab/genewalk [71]) for the scientific community to facilitate functional genomics experiment interpretation and data-driven hypothesis generation.

## Methods

### Assembly of mechanistic networks using INDRA

We used the Integrated Network and Dynamical Reasoning Assembler (INDRA) system [21] to collect and assemble a set of statements from the scientific literature and pathway databases. INDRA integrates content from (i) multiple natural language processing systems (REACH [72] and Sparser [73]) of primary literature in the minable NCBI corpus and (ii) queries on pathway databases (Pathway Commons [18, 43], BEL Large Corpus [74], SIGNOR [75]). INDRA extracts information about molecular mechanisms from these sources in a common statement representation, which has a rich functional semantic with respect to reactant and reaction types. Each statement represents a mechanistic relationship (e.g., activation/inhibition, regulation of amount, or post-translational modification) between two entities or between an entity and a biological process. For each data set described in this study, we queried the pathway databases and machine reading results from REACH and Sparser (run on all Medline abstracts and PubMedCentral manuscripts) for interactions involving the DE genes in the dataset. The resultant set of statements consisted only of relationships among DE genes, GO terms, and protein families and complexes containing the DE genes, obtained from the FamPlex ontology [37]. The final set of statements was then used as an input to the core GeneWalk algorithm as described below.

### Assembly of GeneWalk network with gene regulation, GO ontology, and annotation

To generate the gene network from each context-specific set of INDRA statements, we initialized a networkx (v2.2) multigraph in Python (v3.6) and defined from all statements with at least two different agents (human DE genes, their gene family names and/or GO identifiers), nodes for each agent and edge for the reaction itself (with edge label the reaction type). We added edges (label: “FPLX:is_a”) between genes and (if already present in the network) any corresponding gene family names according to relations defined with FamPlex [37].

When using Pathway Commons (PC) as a source for the gene reactions, we downloaded a simple interaction format (NodeA <relation_type> NodeB) PC network (PathwayCommons11.All.hgnc.sif.gz) from pathwaycommons.org, loaded the PC network as a networkx multigraph (with edge label the relation type), and maintained only the subnetwork of nodes (and existing edges between them) that corresponded to human DE gene symbols. When using a mouse DE gene list as an input, the MGD identifiers are first mapped to their human ortholog HGNC identifiers and gene symbols with INDRA’s integrated HGNC and MGD mappings [47, 48] (INDRA Python package v1.12) before proceeding with the network assembly steps described above.

Next, for each gene in the network (originating from either INDRA or PC), we added GO nodes and edges (label: “GO:annotation”) for each GO annotation (gaf-version: 2.1) as imported with GOAtools [4] (v0.8.12) by matching the gene’s UniProt identifier, an attribute provided by INDRA. We only included annotations without a “NOT” qualifier and based on manually reviewed, possibly phylogenetically inferred experimental evidence, i.e., those with the following GO evidence codes [7]: EXP, IDA, IPI, IMP, IGI, IEP, HTP, HDA, HMP, HGI, HEP, IBA, and IBD. Finally, we imported the GO ontologies (Biological Processes, Molecular Function, and Cellular Component, release 2018-06-20), again using GOAtools, and added to the network the remainder of GO term identifiers as nodes and parent relations from the ontology as edges (label: “GO:is_a”). For generality, we also provide a network assembly option: “edge_list,” which allows the user to provide a predefined GWN in an edge list format (text file in which each line indicates an edge connecting respective node pairs: NodeA NodeB), or “sif” (simple interaction format, as mentioned above). It is assumed that the nodes are either gene symbols or GO identifiers.

### Network representation learning using random walks

To learn the vector representations of all nodes in the GWN, we implemented a version of the unsupervised machine learning algorithm DeepWalk [33] in Python (v3.6) with all hyperparameters (L, N_iteration and dim_rep as described below) optimized to ensure the functionality and reproducibility of GeneWalk. The existence of different types of evidence can generate multiple edges between a node pair. In order to generate a network that reflects the unique nature of molecular interactions, we collapse such multiple edges, thereby reducing the network from a multigraph to a graph. Thus, for our purposes, the degree *d*(*n*) of a node *n* represents the number of nodes connected by at least one edge in the multigraph. We then sample random walks over the network. A random walk over a network represents a random sequence of nodes that are each directly connected by an edge. The probability *p* to jump from node *n* to any connected node equals *p* = 1/*d*(*n*). To sample the local neighborhood of a node *n*_1_, we start in n_1_ and sample a short random walk of a total of *L* = 10 nodes for *d*(*n*_1_) times and perform this procedure for each node in the network. To ensure the reproducibility of the resultant vector representations by having sufficient amounts of sampled walks, we repeat the above procedure *N*_iteration_ = 100 times. Longer walk lengths were tested (*L* = 100, 200, 400, 800, 1600, 4800) but are generally unsuitable for querying the local neighborhood of each node due to the high network connectivity of the GWN (Additional file 1: Supplementary Fig. S3C). Because the effective node distance traveled for a random walk scales with L^1/2^, shorter walk lengths would not sufficiently sample the local node neighborhood and were therefore not considered. Lower numbers of iterations (*N*_iteration_ = 1, 2, 4, 8, or 16) resulted in irreproducible similarity values due to stochastic sampling variation, whereas greater numbers of iterations (*N*_iteration_ = 200) or 50 (Additional file 1: Supplementary Fig. S2) did not alter our results relative to *N*_iteration_ = 100.

As described in the main text (Fig. 1c), the sampled random walks provide a collection of neighboring node pairs, which in turn form a training set of input–output pairs for a fully connected neural network (NN) with one hidden layer of dimensionality *d*. The NN input and output layers are one hot encodings of all nodes from the GWN. In practice, and as previously described for DeepWalk [33], this NN is trained through implementation of the word2vec algorithm [76] (in our case, with gensim package v3.7.1 with the following options: skip-gram model with *k* = 5 negative sampling words and without downsampling: sample = 0 and min_count = 0 and window/context size = 1, dimension dim_rep = 8; for further documentation see https://radimrehurek.com/gensim/models/word2vec.html). Intuitively, our sampled random sequences of nodes are analogous to sentences, which are then used for training to convert words (nodes) into vector representations. When the window size in word2vec is set to 1, it only considers directly connected node pairs from random sequences. Formally, the loss objective of the word2vec NN with input word *w*_*I*_ and output word *w*_*O*_ is [76]: $\log \sigma \left(\upsilon {\hbox{'}}_{\omega_O}^{\top }{\upsilon}_{\omega_I}\right)+\sum \limits_{i=1}^k{\mathbbm{E}}_{\omega_i\sim {P}_{noise}}\left[\log \sigma \left(\upsilon {\hbox{'}}_{\omega_i}^{\top }{\upsilon}_{\omega_I}\right)\right]$_,_ with *P*_*noise*_(*ω*) ∝ *U*(*ω*)^3/4^ and *U* (*ω*) the unigram distribution. Here, ${\upsilon}_{\omega_I}$ represent the input weights of *w*_*I*_, which constitute the vector representations used for our GeneWalk analysis, and ${\upsilon}_{\omega_o}^{\prime }$ the output weights for *w*_*O*_. For the vector dimensionality dim_rep, we tested different values (2, 3, 4, 6, 8, 12, 16, 32, 50, 500) and found that dim_rep = 8 was optimal because the variance of the resulting cosine similarity distributions was largest, indicating the highest sensitivity of detection of similarity between node pairs. Lower dimensionality generally resulted in high similarity between all nodes, whereas higher dimensionality lowered all similarity values; both cases resulted in a reduced variability. After training, for any input node from the GWN, the resultant hidden layer weights form the vector representation of that node (Fig. 1c). In practice, the gensim package provides a dictionary (gensim.models.word2vec.wv) with all the resultant node vectors, which can then be used for significance testing as described below.

### Determining statistical significance of GeneWalk similarity values

For similarity significance testing, we first generated a randomized network from the GWN, i.e., a network with the same number of nodes as in the GWN but with edges randomly permuted such that the GWN degree distribution is retained (networkx v2.2 configuration_model function) [77]. With this random network, we proceed with network representation learning as described above for the GWN to generate random node vectors, which are then used to form null distributions of cosine similarity values (gensim wv.similarity function). For each node *n* in the random network, we calculate the cosine similarities with all its neighbors to form a null distribution. We repeat this for nreps_null = 10 independently randomized networks and collate the similarity values from all replicates to assemble a sufficiently large null distribution. Next, we proceed with significance testing for each connected gene–GO term pair present in the GWN. The *p* value for such a pair then equals the normalized rank of the cosine similarity in the null distribution. To correct for multiple testing across all gene–GO term pairs (global *p*-adjust) or for all GO annotations per gene (gene *p*-adjust), we utilized the Benjamini–Hochberg FDR adjusted *p* value (Python package: statsmodels, function: stats.multitest.fdrcorrection). Finally, we repeat the above-described network representation learning and significance testing procedures of the GWN nreps_graph = 10 times and provide the mean and 95% confidence intervals of global and gene *p*-adjust as our final outputs alongside the mean and standard errors (s.e.m.) of the generated gene–GO pair similarity values.

GeneWalk methods and analyses presented in this study were run with code release version v1.0.0 [78] (stage = node_vectors, null_distribution) and GeneWalk v1.3.0 [79](stage = statistics), unless stated otherwise, which are available as Python packages and on github.com/churchmanlab/genewalk [71]. All arguments are described on the README page of our repository [71]. In particular, GeneWalk v1.1.0 [80](and beyond) have nreps_graph and nreps_null set to 3 by default to reduce the run time. Results correlate strongly with 10 repeats for each (Additional file 1: Supplementary Fig. S2). Recommended memory availability on operating system: 16 Gb or 32 Gb RAM. Recommended number of processors (optional argument: nproc) for a 1–2-h run time is 4 (default 1, run time 6–12 h). Future software update changelogs will be made available as Github releases on github.com/churchmanlab/genewalk/releases [71]. GeneWalk v1.2.0 (and beyond) includes all input genes, irrespective of whether they are directly connected or not, since the results correlate strongly for connected genes (Additional file 1: Supplementary Fig. S2). As a consequence, GeneWalk can run with any number of input genes, but the context generally becomes better defined in the presence of many (> 10) input genes. When preparing an input gene list from for instance a differential expression analysis, it is recommended to use a relatively tolerant DE FDR cutoff value of 0.05 to ensure sufficiently many genes of interest are included for GeneWalk analysis.

### Differential expression analysis of mouse RNA-seq

Mouse *Qki* deletion RNA-seq experiments and DE analysis were described previously [45]. The DE results are re-visualized in Additional file 1: Supplementary Fig. 1B for completeness.

### Differential expression analysis of NET-seq

JQ1 and IsoG NET-seq experiments were previously described in [55, 61], respectively, and the data are available in GEO accession number GSE79290 and GSE86857. In brief, MOLT4 cells (two biological replicates per condition) were treated either with JQ1 (1 μM, 2-h treatment) or DMSO (negative control). For the IsoG study, HeLa S3 cells (two biological replicates per condition) were treated with IsoG (30 μM for 6 h) or DMSO control. NET-seq, 2 replicates, DMSO control.

We generated NET-seq coverage files [59] with modifications described below. Here, we used NET-seq alignment scripts available at [81]. Briefly, we utilized 5′ random hexamers as UMIs by displacing them from the .fastq read sequence and aligned the resultant reads with STAR (v2.5.1a) with genome assembly GRCh38 and annotation from GRCh38v86. We filtered out multimapping alignments, PCR duplicates, and RT mispriming reads (i.e., cases in which RT priming occurred at a position within the RNA fragment instead of the 3′ end), and splicing intermediates. Finally, we generated NET-seq coverage files (.bedgraph format) at nucleotide resolution with HTseq (v0.9.1) using the whole read length.

The coverage files were imported into R (v3.5.0, packages: GenomicRanges v1.32.3, rtracklayer v1.40.3) to determine gene coverage, i.e., the sum over base-pair counts, using Ensembl gene_ids. We filtered for protein-coding genes (annotation acquired with package biomaRt v2.36.1) with positive coverage, i.e., counts per gene averaged over all conditions > 20. The resultant genes and their counts were then utilized to determine differentially expressed genes with DEseq2 [2] (v1.20, default parameters except as follows: FDR = 0.001, betaPrior=false and poscount size factor estimation (JQ1) or total read count as size factor for IsoG). After differential expression, we filtered for genes with an HGNC identifier and gene symbol to ensure INDRA could accept them as an input.

### Context-specific gene-function benchmarks

Through systematic, manual text mining, we first tabulated all (gene, biological context term) pairs (Additional file 3) mentioned together in [45] for the *qki*-context. Biological context terms are broadly defined as anything related to biology. Only the abstract, main text, tables, and figures with legends were used for text mining. For the JQ1-context, we performed the same procedure on [55], the primary publication that described the JQ1 NET-seq experiment, as well as the abstracts from Sanda et al. [56] and Sharma et al. [57] (Additional file 3). For each context, in Additional file 3, we list the genes and biological context terms, the corresponding evidence (text or figures) from the reference publication, a “NOT” qualifier in case the text contains a negation, indicator for the gene’s DE status and the human ortholog for mouse genes (in the *qki*-context). Gene–context terms pairs with a “NOT” qualifier were not used for downstream benchmark analyses. Likewise, genes that are not DE were not utilized by GeneWalk and the other functional analysis methods and could thus not be further considered. Then with this tabulation, we made use of regular expression (Python package: re, function: search) to identify for each (gene, biological context term) pair, the gene’s GO annotations that contain that biological context term as a substring. In this way, relevant (gene,GO annotation) pairs were identified through manual text mining with the aid of a regular expression script. We assigned relevant (gene, GO annotation) pairs with value bm_truth = TRUE (Additional file 3, sheet Benchmark_qki and Benchmark_JQ1). All GO annotations without a matching context term are considered not relevant and are assigned a bm_truth = FALSE value. The ground truth rank ordering for each gene is (tied) rank 1 for all relevant GO annotations with bm_truth = TRUE and (tied) rank 2 for all with bm_truth = FALSE. For the extended ground truth benchmark (Additional file 1: Supplementary Fig. S1H, S3H), we also assigned rank 1 to any GO annotations that were (possibly indirect) parents, as determined with GOAtools [4], of another (direct) GO annotation with bm_truth = TRUE. Further details of the GO annotation rank ordering and comparison with other methods are detailed in the following methods section.

### Systematic comparison of GeneWalk with alternative methods

For GeneWalk, we utilized its gene-padj output values to directly compare with the FDR-adjusted *p* values (padj) of the alternative methods (described in detail in Additional file 1: Supplementary Methods). A custom script in Python (v3.6) was written to append the padj value to each DE gene–GO annotated pair if the GO term was enriched (FDR = 0.1) according to each alternative method. Some methods did not provide any results on GO terms that were not enriched (padj > 0.1). As these cases are not statistically significant, the actual (gene-)padj values, i.e., gene-padj in the case of GeneWalk and padj for all other methods, are less informative for relevance ranking. For these reasons, we classified cases with (gene-)padj ≥ 0.1 for all methods as having (gene-)padj = 1 for our comparison analysis. For each gene, the overall GO term relevance ranking is then the (gene-)padj values in ascending order with the understanding that equal values result in a tie ranking. MGSA is the only Bayesian method that outputs a posterior probability reflecting global relevance to the input gene set, instead of a frequentist *p* value. To make MGSA directly comparable with all other methods, each GO term Bayesian posterior probability > 0.5 was considered statistically relevant with ranking based on the posterior probability in descending order. We also tested a more stringent posterior probability > 0.95, but it made little difference as 0.5 already resulted in very few significantly relevant GO terms. So for MGSA, the effective conversion formula to translate the posterior probability ppost into an equivalent padj value for our rank order comparison is then padj = 1- (ppost/10) if ppost > 0.5 and padj = 1 if ppost ≤ 0.5. To ensure that the rank order correspondences of GeneWalk are quantitatively robust against stochastic variation between runs and parallelization code implementations, we included the model predictions on *qki* and JQ1 from 4 runs: 2 independent seeds (42 and 1234) of GeneWalk v1.0.0 [78] (stage = node_vectors, null_distribution, nreps_graph = 10, nreps_null = 10 and nproc = 8) and the same parameters with improved parallelization code GeneWalk v1.1.0 [80]. No qualitative and only minor quantitative differences were observed between each GeneWalk run, providing confidence in the robustness of our approach. For the “parentally enhanced” methods (Additional file 1: Fig. S1I, S3I), we assigned to any insignificant direct GO annotation (i.e., with (gene-)padj ≥ 0.1), the significant (gene-)*p*-adjust value from a possibly indirect parent (as determined with GOAtools [4]). In case multiple parents had significant (gene-)*p*adj values, their minimal value was assigned.

To determine the rank order correspondence of each method with a ground truth for the myelin genes *Mal*, *Pllp*, and *Plp1* in the *qki*-context, we determined for the list of GO annotations of these genes*,* with regular expression (Python package: re, function: search) if it contained the string “myelin.” If so, a GO annotation was labeled relevant and assigned rank 1. If not, it was labeled not-relevant and assigned rank 2. The rank order of each method was according to (gene-)padj in ascending order, with the understanding that two consecutively ordered significant terms that are relevant are tied in their ranking, because we have no ground truth on which myelin GO annotation is more relevant. For the same reason, all insignificant (padj ≥ 0.1) GO annotations also tie their ranking. With the systematic ground truth benchmarks for the *qki* and JQ1-contexts (Additional file 3), we took the same approach: for the DE gene-biological context term pair from the benchmarks, we searched all GO annotation of that particular DE gene for presence of the respective biological context term and assigned the labels and ranks as described above for biological context term “myelin”. All genes with at least one GO annotation labeled relevant according to the ground truth were then used for downstream analyses. We calculated for each gene the Kendall’s tau rank order correspondence (Python package: scipy, function: stats.kendalltau) between the ranking of the method and the ground truth. This method was used because it can deal with ranking ties better than the Spearman rank correlation. In case all GO annotations were called insignificant by a method, the rank order of all terms is tied, resulting in Kendall’s tau function output: NaN. Because it is understood that there is no correspondence with the ground truth in this case, the NaN value was set to zero. In case all ground-truth ranking of all GO annotations was 1 for both the ground truth and the model prediction, Kendall’s tau function output was also NaN. However, the predicted rank order corresponds perfectly to the ground truth, so the Kendall’s tau value was set to 1. The GeneWalk kendall’s tau values of each gene from the 4 replicate runs described above were averaged to enable a pairwise comparison with each alternative method. To assess the statistical significance of the difference between the Kendall’s tau distribution over all benchmark genes for GeneWalk and versus each alternative method, a (non-parametric) Wilcoxon signed-rank sum test (Package: Scipy, function stats.wilcoxon, argument: correction=False, alternative=“greater”) was used.

Gene–GO annotations from the *qki* and JQ1 contexts ground truth benchmarks are labeled relevant or not, as described above. When assessed as a binary classification problem for each of these gene–GO annotation pairs, the area under receiver operating characteristic (AUROC, Package: scikit-learn, function: roc_auc_score) and ROC curves (Package: scikit-learn, function: roc_curve) over all pairs was determined by comparing each method using the quantitative relevance score -log_10_(*p*-adj+10^−16^) to these benchmark binary ground truths. We also calculated the macro-AUROC and micro-AUROC, where the macro-AUROC is the average of the AUROC values from the *qki* and JQ1-context. The micro-AUROC is determined through taking the union of all model predictions from the two contexts and then calculating the AUROC as described above.

To compare the performance of all methods against random selection, we generated a negative control model as follows. For each DE gene g in the benchmark (with at least one GO annotation labeled relevant according to the ground truth), we randomly selected *N*^*g*^ GO annotations to be predicted as relevant, with *N*^*g*^ a uniformly distributed integer between 0 and the total number of GO annotations for that gene. We then calculated the Kendall’s tau by comparing to the ground truth ordering as described above. We repeated this procedure 100 times and calculated the resulting mean of each gene’s Kendall’s tau as the final random sampling model prediction. For the binary classification, we calculated the AUROC over the benchmark gene–GO annotation pairs for each of the 100 random samples, after which we average these to get the final random sampling AUROC value.

For all input DE genes combined, we determined the set of gene–GO term pairs that had a (global-)padj< 0.1, i.e., global-padj in the case of GeneWalk and padj for all other methods, and counted the number of different (unique) GO terms represented in this set as a percentage over all unique GO annotations. Lastly, we determined for each GO term its level in the ontology with GOAtools [4] (v0.8.12). We compared the levels for all GO terms from the set of the gene–GO term pairs that were significant with (global-)padj < 0.1 for either GeneWalk only, an alternative method only or both GeneWalk and the alternative method with the Mann-Whitney *U* test (Package: Scipy, function stats.mannwhitneyu, argument: use_continuity=True, alternative=“two-sided”).

### GeneWalk model robustness analysis

For model robustness analysis, we developed eleven GeneWalk test versions (Additional file 1: Supplementary Fig. S2), with each code implementation available at [82]. The default version (label: Connected input genes, Additional file 1: Supplementary Fig. S2) that was used to compare each test version against is GeneWalk v1.1.0 [80] (stage = node_vectors, null_distribution) and GeneWalk v1.3.0 [79] (stage = statistics) with arguments nreps_graph = 10, nreps_null = 10, and random_seed = 42. We also ran the default version with a different seed = 1234 to assess the variability between independent runs (Additional file 1: Supplementary Fig. S2). Unless specified otherwise, each test version was run on *qki* and JQ1 data with both INDRA and PC knowledge bases.

Test versions:

1. All input genes present in the GWN, irrespective of whether it is directly connected with another input gene (requirement in default version).
2. Nreps_graph = 3 and nreps_null = 3 instead of 10. This reduces the run time by a factor of 3 and reflect the default settings in GeneWalk code implementation v1.1.0 and beyond.
3. N_iteration = 50 instead of 100. This results in 50% fewer random walks sampling.
4. With randomization of only gene–gene and gene–GO annotation connections (but not connections originating from the GO ontology) to generate a null distribution of similarity values. These random similarity values are now only calculated between (randomly connected) gene–GO annotation pairs as opposed to all node pairs in the partly randomized graph.
5. With all model features from test version (1), (4) and the extra feature: starting random walks from gene or GO annotation nodes only in both the GWN and randomized graphs. The difference with (4) is that the importance of the (context-independent) GO ontology network in informing the vector representations is decreased. The GO ontology is highly structured resulting in higher random gene–GO annotation cosine similarities in test version 4 as opposed to this test version 5 where the GO ontology is undersampled (Additional file 1: Supplementary Fig. S2C, null distribution cumulative distributions). So test version 5 is a more context-specific model than 4 and the default version. Furthermore, test 5 version’s null distribution is determined in the most principled manner. Test version 5 could therefore be preferred, but it has the major practical drawback (as also for test 4) that, for short gene lists, the null distribution remains underpopulated since only randomized gene–GO annotation pairs are used to generate the null distribution. The default version does not suffer from this problem and its null distribution lies in between cases (4) and (5). Notably, in the regime of high random similarity values (> 0.8), which are most important in determining the *p* values, test (4), (5), and the default null distributions do not differ substantially (Additional file 1: Supplementary Fig. S2C, null distribution cumulative distributions). So overall, the default randomization version is a robust approximation to test versions (4) and (5) that works well for any input gene list.
6. Without the GO ontology. Only GO annotations connected to genes are present in the GWN.
7. With added (direct) connections between gene and all (normally not-connected) parent GO terms of a directly connected (child) GO annotation (transitivity property). A parent GO term is defined through an “is_a” edge attribute between the child and parent nodes as determined with GOAtools.
8. With all expressed genes in the genome as input rather than the (context-defining) DE genes. This test version is only run with Pathway Commons as knowledge base in absence of availability of the full INDRA knowledge base.
9. With node2vec [34] instead of DeepWalk [33] as network representation learning method. Node2vec differs from DeepWalk as it samples biased random walks defined through two added parameters: “return” parameter p controls the likelihood of immediately revisiting a node in the walk and parameter q allows the search to differentiate between “inward” and “outward” nodes [33]. We performed a parameter scan with *p* and *q* values elements of [0.25, 1, 4], all with nreps_graph = 3 and nreps_null = 3 to limit the run time. Despite, the increased model complexity of these two added parameters, this test version did not improve performance on our ground truth tasks (best performing model, with *p* = 4 and *q* = 1, prediction shown in Additional file 1: Supplementary Fig. S2B,E) and globally the model correlated well with the control version (Additional file 1: Supplementary Fig. S2C,F).
10. With very long random walk lengths: *L* = 1000 steps and *N*_iteration_ = 1. This version approximates a network representation learning algorithm through spectral decomposition (matrix factorization) as it is mathematically equivalent to DeepWalk with infinite walk lengths [53].
11. No gene–gene connections. Note that this model does not use any input from INDRA or PC. All input DE genes are added to the GWN as nodes and connected to the GO ontology through their GO annotations (if any).

### Likelihood ratio test for uniform distribution of relevant GO terms

To assess how the number of relevant GO terms relates to the number of connected GO terms (Fig. 3h), we developed a likelihood ratio test. Without loss of generality, this test is also applicable to other described cases (Fig. 5e, Additional file 1: Supplementary Figure S3A-B, S6C-D) where the random variable *Y* described on the *y* axis (number of relevant GO terms in Fig. 3h) has the intrinsic dependency *Y* ≤ *X* on a random variable described on the *x* axis *X* (number of connected GO terms in Fig. 3h). First, note that for any discrete joint probability distribution ℙ(*X*, *Y*), we have a conditional probability relation: ℙ(*X* = *x*, *Y* = *y*) = ℙ(*Y* = *y*|*X* = *x*)ℙ(*X* = *x*). The null hypothesis *H*_0_ for our likelihood ratio test is that *Y*|*X* is uniformly distributed between 0 and *X*: $\mathrm{\mathbb{P}}\left(\left.Y=y\right|X=x\right)=\frac{1}{x+1}$. The alternative hypothesis *H*_1_ is that the conditional probability is not uniform, but instead determined by a priori unknown probabilities: ℙ(*Y* = *y*|*X* = *x*) = *p*_*x*_(*y*).

For any given *x*, if we repeatedly observe *Y*|*X=x* for a total of *N*_*x*_ multiple independent times, the joint frequency function, i.e., the collection of numbers of times {*n*_*y*, *x*_} each *y* ∈ {0, 1, ⋯, *x*} value is observed, follows a multinomial distribution [83]: $$ \mathrm{\mathbb{P}}\left(\left.\left\{{n}_{y,x}\right\}\right|x\right)=\left(\begin{array}{c}{N}_x\\ {}{n}_{0,x}\cdots {n}_{x,x}\end{array}\right)p{\displaystyle \begin{array}{c}{n}_{0,x}\\ {}y=0\end{array}}\cdots p{\displaystyle \begin{array}{c}{n}_{x,x}\\ {}y=x\end{array}} $$, with *p*_*y*_ equal to the above described condition probabilities specific for each hypothesis.

The likelihood ratio Λ [83] is by definition the ratio of joint probabilities functions under each hypothesis with maximum likelihood estimated (MLE) parameter values given our observed data {(*X*_*i*_, *Y*_*i*_)}*i* = 1. . *N*: $\Lambda =\frac{{\mathbb{P}}_{H_0}\left({\left\{\left({X}_i,{Y}_i\right)\right\}}_{i=\mathrm{1..}N}\right)}{{\mathbb{P}}_{H_1}\left({\left\{\left({X}_i,{Y}_i\right)\right\}}_{i=\mathrm{1..}N}\right)}=\frac{{\mathbb{P}}_{H_0}\left(\operatorname{}{\left\{{Y}_i\right\}}_{i=\mathrm{1..}N}|{\left\{{X}_i\right\}}_{i=\mathrm{1..}N}\right)}{{\mathbb{P}}_{H_1}\left(\operatorname{}{\left\{{Y}_i\right\}}_{i=\mathrm{1..}N}|{\left\{{X}_i\right\}}_{i=\mathrm{1..}N}\right)}$. In our case, this is the ratio of multinomial distributions with probabilities defined by each hypothesis. Under *H*_1_ the MLEs equal [83]: ${\tilde{p}}_x(y)=\frac{n_{y,x}}{N_x}$ with ${N}_x={\sum}_{y=0}^x{n}_{y,x}$ the total number of y observations for a given *x*. On the other hand under *H*_0_, the uniform distribution fully determines the probabilities and are thus independent of our observations:${\tilde{p}}_x(y)=\frac{1}{x+1}$. Now let *x*^*max*^ ≔ max_*i* = 1. . *N*_*X*_*i*_ be the maximum observed X value. Thus, our likelihood ratio reduces to:

$$ \Lambda =\prod \limits_{x=1}^{x^{max}}\frac{{\left(\frac{1}{x+1}\right)}^{N_x}}{\prod_{y=0}^x{\left(\frac{n_{y,x}}{N_x}\right)}^{n_{y,x}}}. $$

The log-likelihood ratio then simplifies to:

$$ -2\log \left(\Lambda \right)=-2\sum \limits_{x=1}^{x^{max}}\left[{N}_x\log \frac{N_x}{x+1}-\sum \limits_{y=0}^x{n}_{y,x}\log {n}_{y,x}\right]. $$

Finally, we use the theorem that the log-likelihood ratio follows a chi-square distribution $-2\log \left(\Lambda \right)\sim {\chi}_k^2$, with k (the number of degrees of freedom) determined as the difference between the number of unknown parameters of the null and alternative parameters [83]. In our case, $k=\frac{1}{2}{x}^{max}\left({x}^{max}+3\right)-0$. This enables us to perform our likelihood ratio test. We calculated the log-likelihood ratio in Python and utilized the scipy.stats.chi2.sf function to determine the *p* value of our test statistic.

## Supplementary Information

**Additional file 1.** Supplementary figures, legends and methods.

**Additional file 2. **GeneWalk outputs using INDRA or PC as the knowledge bases for differentially expressed genes from the *qki*, JQ1 and IsoG contexts.

**Additional file 3. **Gene function benchmark ground truth and model predictions for *qki* and JQ1 contexts. The sheets named after the publications used to generate the ground truth (SciRep2016Darbelli, MolCell2016Winter, CancerCell2012Sanda_abstract and CancerRes2018Sharma_abstract) contain all relevant genes, biological context terms and text sections extracted from the respective publications (all columns are further described in detail in Materials and Methods section: Context-specific gene-function benchmarks). The remaining two sheets (Benchmark_qki and Benchmark_JQ1) contain all the DE genes and their GO annotations that corresponded to the benchmark genes. For GW (INDRA) the connected GO terms originating from INDRA through its text mining are also listed. The column bm_truth indicates the binary ground truth: TRUE if any context term from the ground truth publications matched the GO term through regular expression and FALSE otherwise (see Methods section: Context-specific gene-function benchmarks for details). Also shown are the FDR adjusted *p*-values (padj) values for each method and the corresponding binarized values (TRUE if padj< 0.1, FALSE otherwise). These were used to compare the performances of each method against the ground truth values.

**Additional file 4.** Twenty genes identified in JQ1 condition with more than 40 GO annotations of which at most 50% were relevant. (CSV 1 kb)

**Additional file 5.** Review history.

## Data Availability

GeneWalk is available as a Python package and can be run as a standalone program. Code is available at https://github.com/churchmanlab/genewalk [71] under the open source BSD-2 license. The software used in this publication has also been archived in additional publicly available repositories [78–82]. Instructions to install and run GeneWalk are described in a tutorial at http://churchman.med.harvard.edu/genewalk. JQ1 and IsoG NET-seq experiments were previously described in [55, 61], respectively, and the data are available in GEO accession number GSE79290 [84] and GSE86857 [85].
