# Supplementary material for: GeneWalk identifies relevant gene functions for a biological context using network representation learning
Source: Genome Biol. 2021 Feb 2;22:55. doi: 10.1186/s13059-021-02264-8 (PMC7852222; doi:10.1186/s13059-021-02264-8)
Supplement: Supplementary file 1 — Additional file 1. Supplementary figures, legends and methods. [file 13059_2021_2264_MOESM1_ESM.pdf]

Additional File 1

- Supplementary Figures and legends

- Supplementary Methods

- Supplementary References

Supplementary Figure S1

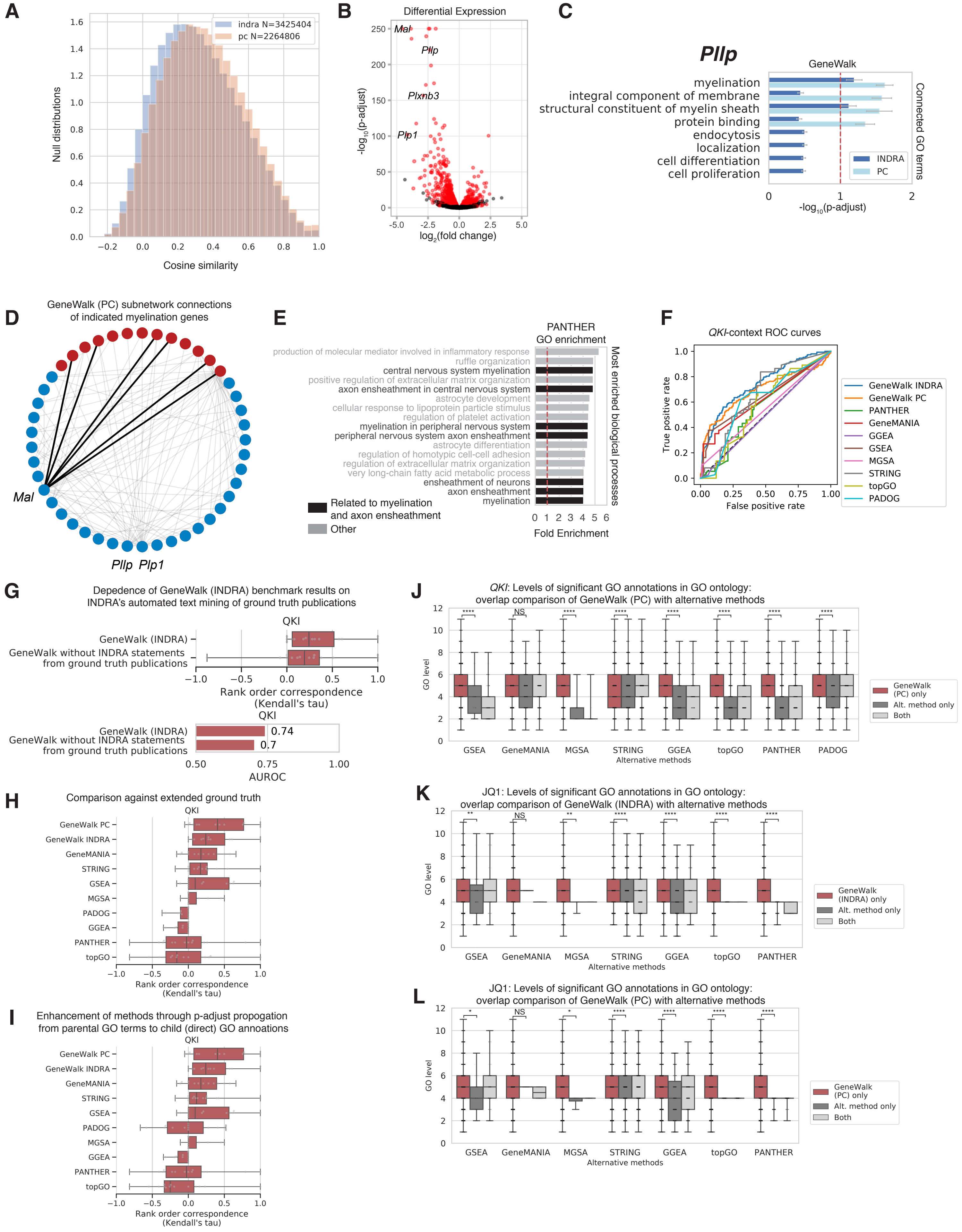

Figure S1
A Histograms of cosine similarity value null distributions from the GeneWalk analyses in the *qki* condition with INDRA and Pathway Commons (PC) as knowledge bases.
B Volcano plot showing the results of a differential expression (DE) analysis that compares gene expression profiles in mouse brains with/without *Qki* deletion, re-visualized from[45]. DE genes (N=1899) are indicated in red, which are used as an input to GeneWalk. All other genes are depicted in black.
C GeneWalk results for *Pllp* in the *qki*-condition using either INDRA or PC as a knowledge base source to assemble the GeneWalk network (GWN). All GO terms connected to *Pllp* are rank-ordered by Benjamini-Hochberg FDR-adjusted p-value (p-adjust), indicating their functional relevance to *Mal* in the context of *Qki* deletion in oligodendrocytes. Error bars indicate 95% confidence intervals of gene p-adjust. FDR=0.1 (dashed red line) is also shown. Additional file 2 shows full GeneWalk results.
D Visualization of the PC GWN subnetwork of myelination-related genes *Mal, PllP,* and *Plp1*, all their connected genes and GO terms. Edges (grey) connecting node pairs indicate the presence of INDRA reaction statements or GO annotations between the two respective nodes. Edges between *Mal* and its GO connections are highlighted (bold).
E Enriched Biological Process GO terms (top 17 shown of 379 enriched terms; Fisher exact test, FDR = 0.01) in *qki*-condition, ranked by fold enrichment, resulting from GO enrichment analysis with PANTHER[1]. Enriched terms related to myelination and axon ensheathment, as reported previously in Darbelli et al, 2017[45], are highlighted in black. Red line indicates a fold enrichment value of 1, indicating the background.
F Receiver Operating Characteristic (ROC) curves resulting from the gene-function relevance binary classification task for GeneWalk and alternative methods (Table 1) on the *qki*-context ground truth benchmark (Additional file 3).
G Dependence of GeneWalk (INDRA) benchmark performance on INDRA's automated text mining of ground truth publications. Kendall's tau rank order correspondence and area under ROC (AUROC) metrics on the *QKI* and JQ1 benchmarks are determined as described in Figure 3D and E respectively.
H Distribution of Kendall's tau rank order correspondences of predictions from GeneWalk and alternative methods (Table 1) to the extended ground truth benchmark for the *qki*-context with parentally-related GO terms of relevant GO annotations are also jointly top-ranked (indicating they are relevant) and all other gene-GO annotations pairs are jointly bottom ranked (not relevant). All methods are ordered by the median of their Kendall's tau distribution, indicating their relative performances.
I As in (**H**) but in this case the original ground truth is used to benchmark the "parentally enhanced" methods where the significant p-adjust values of parentally-related GO terms are propagated down to GO annotations that were itself not enriched.
J Boxplots of the GO term levels of all significant (global p-adjust for GeneWalk and p-adjust for alternative methods at FDR=0.1) gene-GO annotation pairs across across all *qki* DE genes present in the Pathway Commons (PC)-derived GWN. A higher GO level reflects more specific concept information in the GO ontology [7]. Direct overlap comparison of GeneWalk (with PC) with the rankings from alternative methods is indicated with individual data points shown. A Mann-Whitney U-test indicates the statistical differences in median levels between levels significant for

only GeneWalk as compared to only the alternative method, *: $p<0.05$; **$p<0.01$; ***$p<10^{-3}$ and ****$p<10^{-4}$.

K-L As in (**J**) for the JQ1 study with GeneWalk (**K**: INDRA- or **L**: PC-derived GWN).

Supplementary Figure S2

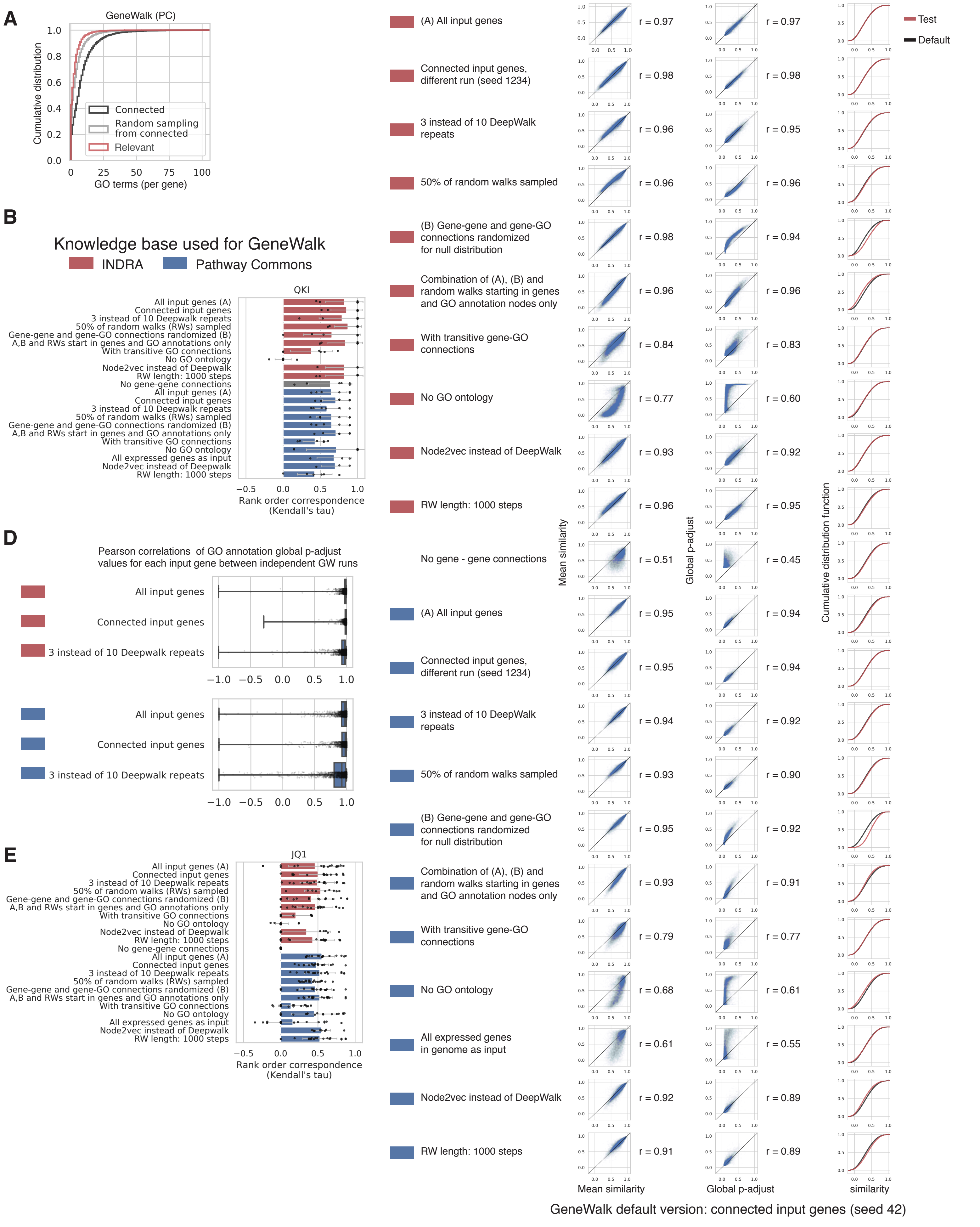

Supplementary Figure S2F JQ1: Test vs default scatter plots connected gene-GO term node pairs

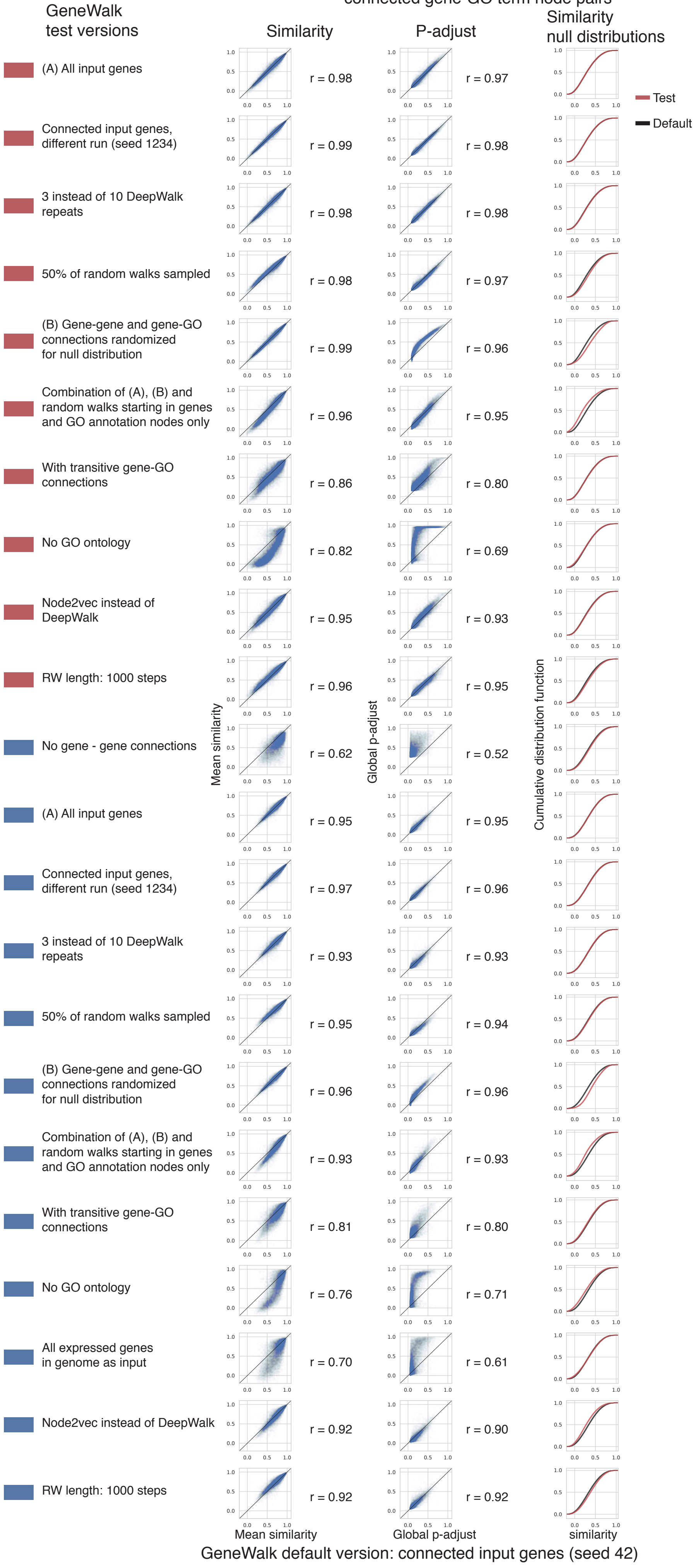

Figure S2

A Cumulative distribution of number of connected (black) and relevant (red) GO terms per gene, alongside a simulation that uniformly randomly sampled from the number of connected terms (grey) for GWNs with PC. The number of relevant GO terms was smaller than with randomly sampling connections (KS test: $p < 1e\text{-}16$).

B Kendall's tau rank order correspondence performance comparison of all GeneWalk test and default (Connected input genes) versions to the same *qki* ground truth ranking which is myelin terms shared 1st and all other annotations shared 2nd . Results indicate mean (and error bars: standard deviation) over the three myelin-related genes *Mal, Pllp and Plp1*, obtained with two independent GeneWalk runs (seed= 42 and 1234). Individual Kendall's tau values are shown (black dots). Color red (blue) indicates use of INDRA (PC) as a knowledge base for GeneWalk. See materials and methods for a detailed description of each GeneWalk test version. The model without gene-gene interactions has no input from a knowledge base and is therefore indicated in grey.

C Correlations scatter plots of all *qki* DE gene-GO annotation pairs in terms of mean similarity and global p-adjust values for GeneWalk test versions with GeneWalk default version (seed 42). All versions as described in (**B**). Also shown are the cumulative distribution functions of the null distributions of each comparison.

D Boxplots with Pearson correlations of GO annotation global p-adjust values for each input gene between independent GeneWalk runs for GeneWalk control (Connected input genes) and two test versions that are also recommended for general use (See materials and methods for details).

E As in (**B**) for the JQ1 study with previously identified transcriptional regulators[55,56,57]: *MYC, MYB, RUNX1, RUNX2, TAL1, SATB1, ERG, ETV6* and *TCF12* (Additional file 3).

F As in (**C**) for the JQ1 study.

Supplementary Figure S3

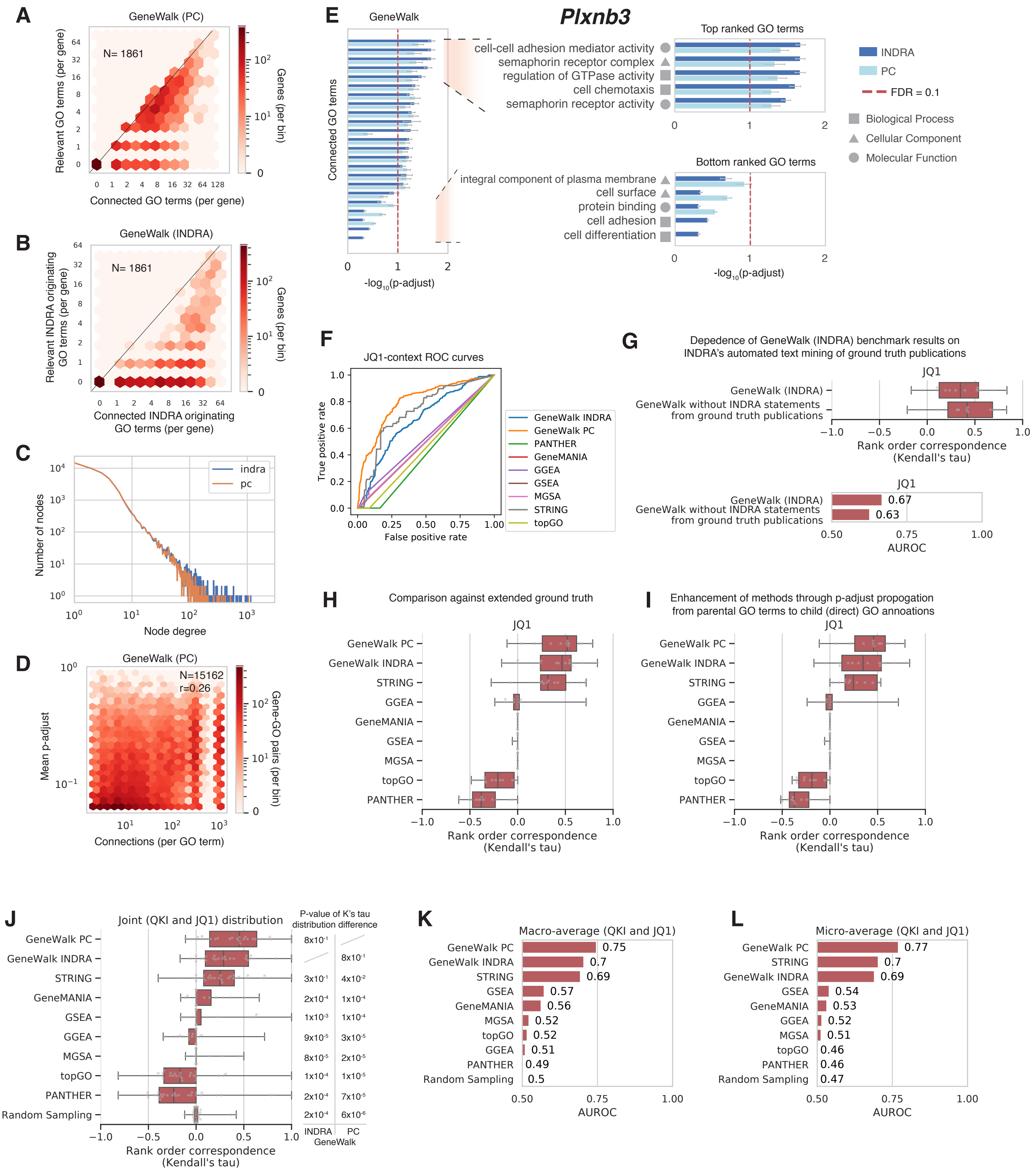

Figure S3

A Hexagon density plot for all genes of interest (N=1861) in terms of number of connected GO terms and number of relevant GO terms (at FDR=0.1) resulting from the *qki* condition GeneWalk using PC as a knowledge base.

B Hexagon density plot for all genes of interest (N=1861) in terms of number of INDRA-originating connected GO terms and number of INDRA-originating relevant GO terms (at FDR=0.1).

C Node degree (number of neighboring nodes) distribution function of *qki* GWNs with INDRA (blue) or PC (orange).

D Hexagon density plot of all tested gene-GO pairs (N= 15162) as a function of GO term connectivity and similarity significance (p-adjust) resulting from the *qki*-condition GeneWalk using Pathway Commons as a knowledge base. Pearson correlation coefficient r=0.26 is also shown.

E GeneWalk results for *Plxnb3* in the *qki*-condition using either INDRA or PC as a knowledge base source to assemble the GeneWalk network. *Plxnb3* is the most strongly downregulated DE gene that also has more than half of its connected GO terms classified as significantly relevant. The top and bottom ranked GO terms are described in the inset. Error bars indicate 95% confidence intervals of gene p-adjust. FDR=0.1 (dashed red line) and domains of GO annotations (square: biological process, triangle: cellular component, and circle: molecular function) are also shown. Additional file 2 show full GeneWalk results using the INDRA or PC knowledge base, respectively.

F Receiver Operating Characteristic (ROC) curves resulting from the gene-function relevance binary classification task for GeneWalk and alternative methods (Table 1) on the JQ1-context ground truth benchmark (Additional file 3).

G Dependence of GeneWalk (INDRA) benchmark performance on INDRA's automated text mining of ground truth publications. Kendall's tau rank order correspondence and area under ROC (AUROC) metrics on the JQ1 benchmarks are determined as described in Figure 4E and F respectively.

H Distribution of Kendall's tau rank order correspondences of predictions from GeneWalk and alternative methods (Table 1) to the extended ground truth benchmark for the JQ1-context where parentally-related GO terms of relevant GO annotations are also jointly top-ranked (indicating they are relevant) and all other gene-GO annotations pairs are jointly bottom ranked (not relevant).

I As in (**H**) but with the original ground truth and "parentally enhanced" methods where the significant p-adjust values of parentally-related GO terms are propagated down to GO annotations that were itself not enriched.

J Joint distribution of Kendall's tau rank order correspondences of predictions from GeneWalk and alternative methods (Table 1) to the union of ground truth benchmarks for the *qki* and JQ1-contexts where all gene GO annotations pairs mentioned in [45,55,56,57] are jointly top-ranked (indicating they are relevant) and all other gene-GO annotations pairs are jointly bottom ranked (not relevant). All methods are ordered by the median of their Kendall's tau distribution, indicating their relative performances. Statistical differences between GeneWalk (INDRA or PC) and other methods are determined with the Wilcoxon signed-rank sum test. See methods for details.

K Bar chart of the area under Receiver Operating Characteristic (AUROC) performance metric for GeneWalk and alternative methods (Table 1). The (macro) average over the AUROC values on the benchmarks from the *qki* and JQ1-context are shown for the binary classification task of identifying gene-function pairs as relevant or not.
L As in (**K**) for the micro-averaged AUROCs: all model predictions from the union over the *qki* and JQ1-context were used to calculate the AUROC.

Supplementary Figure S4

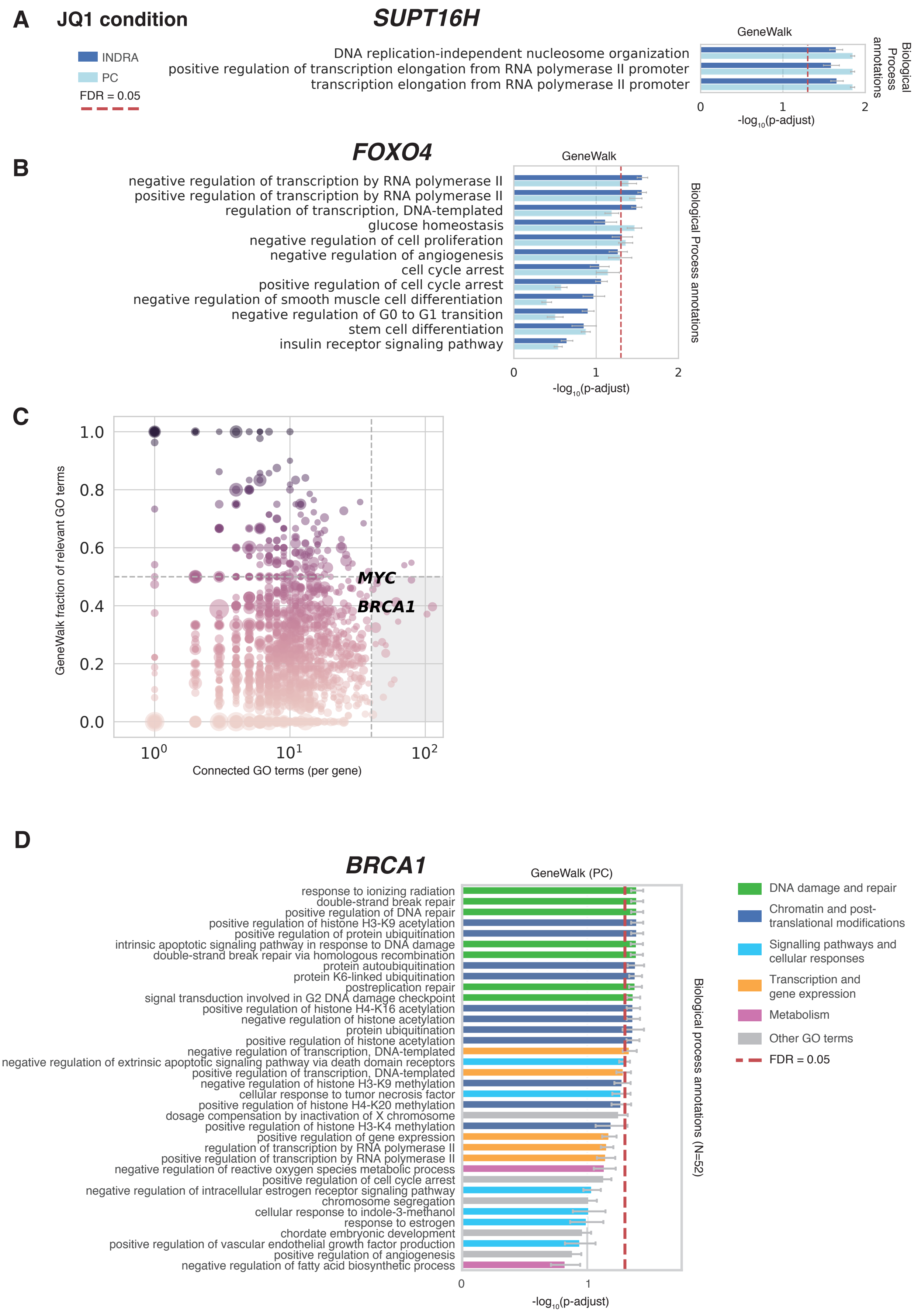

Figure S4

A GeneWalk results for the transcriptional regulator *SUPT16H* under JQ1 treatment. Annotated biological processes are rank-ordered by gene p-adjust. Error bars indicate 95% confidence intervals of gene p-adjust. FDR=0.05 (dashed red line) is also shown. See Additional file 2 for full details.

B As in **(A)** for *FOXO4*.

C Scatter plot with DE genes as data points showing the fraction of relevant GO terms over total number of connected GO terms (min_f, minimum value between INDRA and PC GWNs) as a function of its number of GO connections (N^{GO}). The circle size scales with the differential expression significance strength ($-\log_{10}$(p-adjust)) and the color hue with min_f. Nine genes were identified with min_f < 0.5 and $N^{GO} > 40$ (see Additional file 4 for complete gene list).

D As in (**A**) for *BRCA1* for GeneWalk (PC). Biological process GO annotations are indicated by class: DNA damage and repair (green), Chromatin and post-translational modifications (dark blue), signalling pathways and cellular responses (light blue), transcription and gene expression (yellow), metabolism (purple) and other GO terms (grey).

Supplementary Figure S5

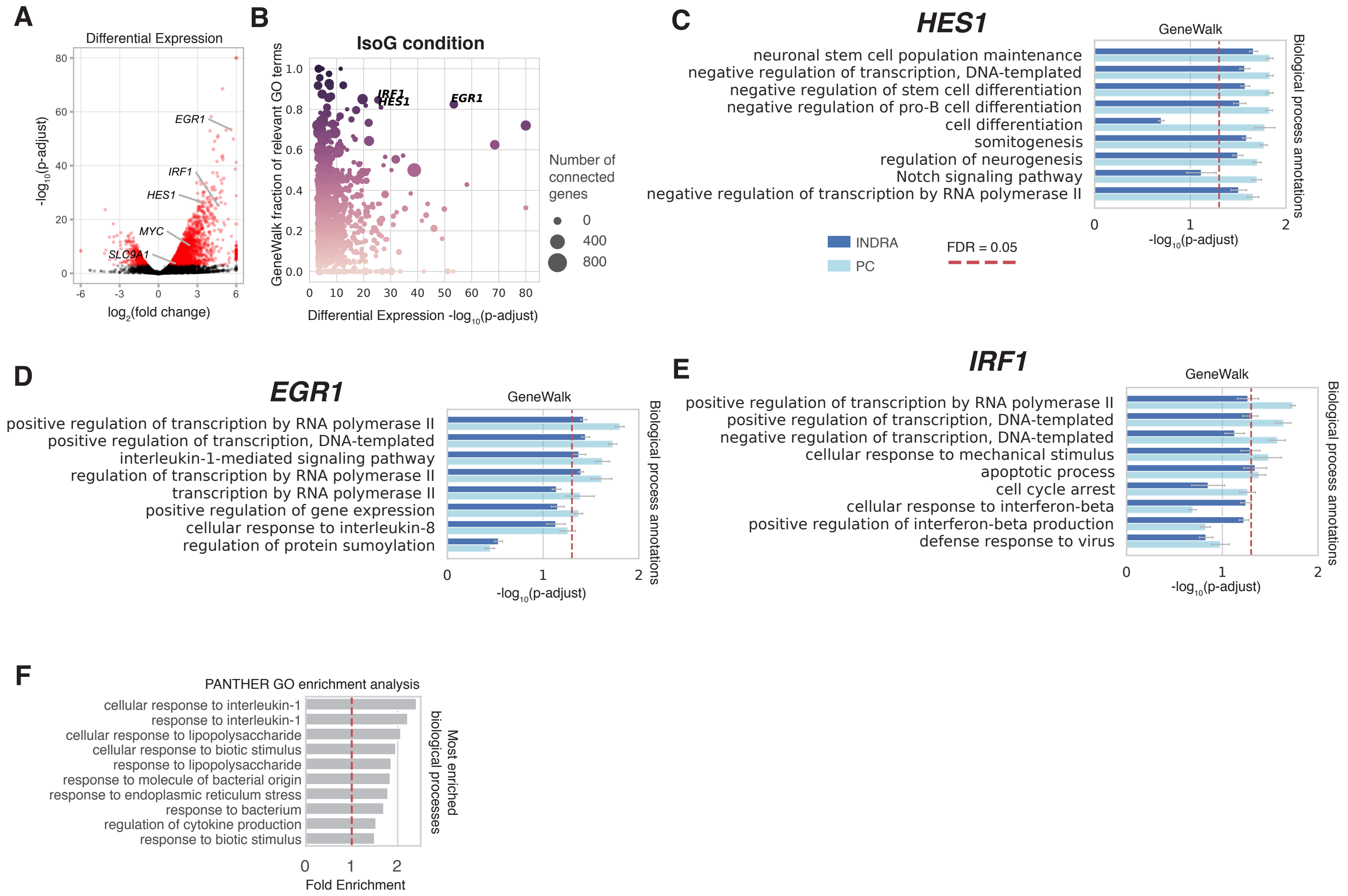

Figure S5

A Volcano plot showing the results of a differential expression (DE) analysis that compares RNA Polymerase II gene coverage between IsoG and DMSO control samples. DE genes (N=2980), indicated in red, were used as an input to GeneWalk. All other genes are depicted in black.

B Scatter plot with DE genes as data points showing the fraction of relevant GO terms over total number of connected GO terms (min_f, minimum value between INDRA and PC GWNs) as a function of the DE significance strength ($-\log_{10}$(p-adjust)). The circle size scales with number of connected genes in GWN (INDRA) and the color hue with min_f.

C GeneWalk results for *HES1* under IsoG treatment with INDRA or PC knowledge bases. Annotated biological process terms are rank-ordered by gene FDR adjusted p-value (p-adjust). Error bars indicate 95% confidence intervals of the gene p-adjust estimate. FDR=0.05 (dashed red line) is also shown. See Additional file 2 for full details.

D-E As in (**C**) for *EGR1* (**D**) and *IRF1* (**E**).

F Enriched Biological Process GO terms (top 10 shown of 41 enriched terms; Fisher exact test, FDR = 0.01) in the IsoG-treated condition, ranked by fold enrichment, obtained by GO enrichment analysis using PANTHER[1]. Red line indicates a fold enrichment value of 1, indicating the background.

Supplementary Figure S6

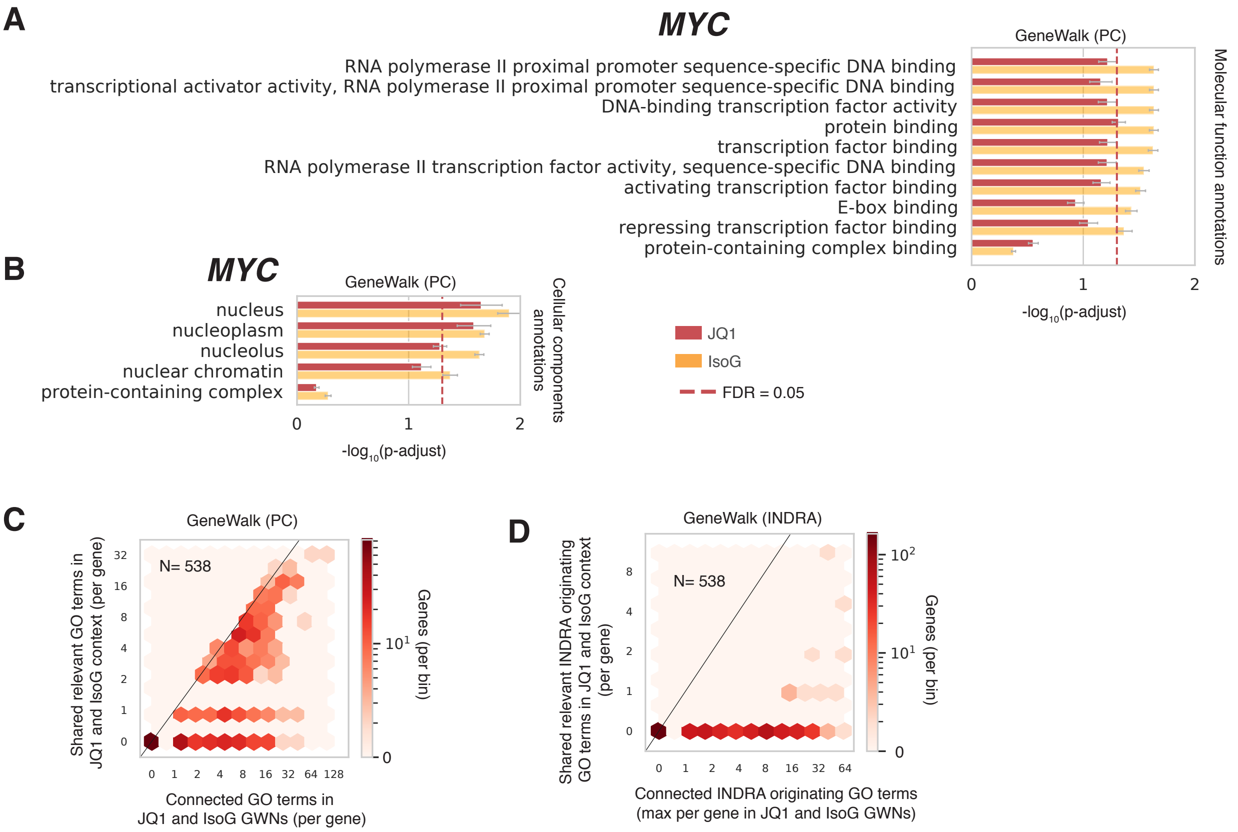

Figure S6
A-B GeneWalk results (with PC as data source) for *MYC* in the JQ1 (red) and IsoG (yellow) condition. Annotated molecular function (**A**) or cellular components (**B**) annotations are rank-ordered by FDR-adjusted p-value to indicate their relative importance under the IsoG condition. See Additional file 2 for full details. Red dashed line indicates FDR=0.05.
C Hexagon density plot for overlapping DE genes (N=538) in terms of number of overlapping relevant GO terms (FDR=0.1) and number of possible shared connected GO terms for the GeneWalk network using PC as a knowledge base.
D Hexagon density plot for overlapping DE genes (N=538) in terms of number of overlapping INDRA originating relevant GO terms (FDR=0.1) and number of INDRA originating possible shared connected GO terms.

Supplemental Methods

PANTHER GO enrichment analysis

To perform GO enrichment analysis with PANTHER[1], we queried the website www.pantherdb.org with options: Overrepresentation Test Released 20181113, GO biological process complete, GO molecular function complete or GO cellular component complete - release 2018-12-01, Fisher Exact test, FDR correction). For JQ1 and IsoG, the analyzed lists consisted of Ensembl identifiers corresponding to the DE genes that were used as an input to INDRA. The reference (background) gene list contained Ensembl identifiers of all genes tested in the DEseq2 analyses. For the *qki* study, GO enrichment analysis was described previously [45] and reproduced here as described for JQ1 and IsoG, with the exceptions that MGD identifiers were used for all the DE genes and reference list, and GO ontology release 2019-01-01 was used.

Gene set enrichment analysis (GSEA)

To apply GSEA[3] (v4.0.2) to our data, we downloaded the GSEA graphical user interface for MacOS from http://software.broadinstitute.org/gsea/index.jsp. Given the data availability (*qki*) and to minimize the effect of global expression biases generating any false positive enriched gene sets (JQ1), we used the GSEAPreranked version on a list with all genes that were tested for differential expression (output from DEseq2) with the Wald statistic ('stat') as the correlation metric used to rank the input genes in a .rnk file. For JQ1 we utilized ensembl gene ids and for *qki* mouse gene symbols as gene identifiers. We selected all GO ontology terms as gene sets (c5.all.v7.0.symbols.gmt) to enable direct comparison with GeneWalk. Other parameters were set according to the website instructions or left as default: collapse Collapse; mode Max_probe; norm meandiv; nperm 1000; scoring_scheme weighted; chip Mouse_Gene_Symbol_Remapping_MSigDB.v7.0.chip (for *qki*) or Human_ENSEMBL_Gene_ID_MSigDB.v7.0.chip (for JQ1); create_svgs false; include_only_symbols true; make_sets true; plot_top_x 150; rnd_seed timestamp; set_max 500; set_min 1; zip_report false.

GeneMANIA

To apply GeneMANIA[5,86] to our data, we downloaded cytoscape for MacOS (v3.7.2 https://cytoscape.org), its GeneMANIA plugin (v3.5.1, http://apps.cytoscape.org/apps/genemania) and subsequently all available human and mouse data sets (v2017-07-13) through the plugin. We then submitted our list of DE genes for *qki* and JQ1 data sets as used for our PANTHER query described above. We ran GeneMANIA with the following settings: automatic weighting; 0 related genes; at most 20 attributes and saved the results panels.

Gene Graph Enrichment Analysis (GGEA)

To apply GGEA[14] to our data, we utilized the EnrichmentBrowser (v2.10.11) and GO.db (v3.6.0) packages in R (v3.5.0) with a custom written Rmarkdown script based on the EnrichmentBrowser vignette. We used as input to GGEA a list with all genes that were tested for differential expression (output from DEseq2) with their respective log2 fold change and DE p-adjust values. For JQ1 we utilized ensembl gene ids and for *qki* mouse gene symbols as input gene identifiers. These input ids were then mapped to entrez ids using the EnrichmentBrowser

function idMap with the appropriate organism identifier org = "hsa" (JQ1) or "mmu" (*qki*), and used to construct a SummarizedExperiment object of the data. The gene regulatory networks were assembled using the compileGRN function with arguments: db="kegg" or "reactome"; act.inh=FALSE. The GO gene sets were assembled with the function getGenesets with parameters: org= set as above, db="go"; go.onto= "BP","CC" or "MF" corresponding to each tested GO domain; go.mode="GO.db". The minimal gene set size was set as GS.MIN.SIZE=1. We then ran GGEA using the function nbea with parameters: method="ggea"; alpha = 0.1; padj.method="BH". The resulting output was generated with function gsRanking and exported to a table (.csv) file for each GO domain and each data set. For our downstream comparison analysis we used the results obtained with the kegg database. However, only for *qki* MF and CC this gave no significant results whilst reactome did, so we chose to present the reactome results for these particular cases.

Model-based Gene Set Analysis (MGSA)
To apply MGSA[13,87] to our data, we utilized the mgsa (v1.28.0), package in R (v3.5.0) with a custom written Rmarkdown script based on the mgsa vignette. We downloaded the mouse (mgi.gaf for *qki*) gene annotation files from the GO website (http://www.geneontology.org) and reused the human version goa_human.gaf (JQ1) that was downloaded for GeneWalk as described above. As input to MGSA we imported our list of DE genes for *qki* and JQ1 data sets as used for our PANTHER query described above. The human ensembl ids were then mapped to UNIPROT ids using the select() function that makes use of the annotation database org.Hs.eg.db R package (v3.6.0). The GO annotation sets were read using the function readGAF() with argument evidence set equal to the evidence codes we used for GeneWalk described above. MGSA results were generated through subsequently calling the function mgsa() with gene list and annotations as arguments and setsResults(). The output comprising all GO annotations from all GO domains with their corresponding Bayesian posterior probabilities, was exported to a table (.csv) file.

Pathway Analysis with Down-weighting Overlapping Genes (PADOG)
To apply gene set enrichment analysis (functional class scoring) with PADOG [15], we utilized the PADOG (v1.24.0) package in R (v3.5.0) with a custom written Rmarkdown script based on the PADOG vignette. Since PADOG requires at least 3 three replicates per condition, PADOG could not be applied to JQ1 as this experiment consisted of 2 biological replicates for both control and JQ1 treatment [55]. For *qki,* we utilized RPKM values of all expressed mouse genes (with MGI identifiers) for all samples [45] as input for PADOG. The GO annotation sets were prepared as described above for MGSA, with the addition that all gene sets were populated with MGI identifiers through the function readGAF@itemName2ItemIndex. PADOG was then run by calling function padog() with arguments: esetm= the gene expression matrix; group=c('c','c','c','d','d','d') reflecting the 3 control and 3 *qki*$^{-}$ genotype samples; gslist= above described GO annotation sets; gs.names= GO term identifiers corresponding to gslist; Nmin=1; dseed=42. The PADOG output consists of all provided GO annotation sets indexed by their GO term identifier and their corresponding Ppadog values, the nominal p-values [15]. These were then corrected for multiple testing using the Benjamini-Hochberg procedure (function p.adjust(method="fdr")), resulting in padj corresponding values. The output was exported to a table (.csv) file.

STRING GO enrichment analysis
To apply GO enrichment analysis with STRING[16] on our data, we utilized the STRINGdb (v1.20.0) package in R (v3.5.0) with a custom written Rmarkdown script based on the STRINGdb vignette. We first set the NCBI taxonomy identifiers 10090 and 9606 for mouse (*qki*) and human (JQ1) respectively. We then initialized the STRING database using the function STRINGdb$new() with arguments: version='10'; species= the above taxonomy identifier. As input to STRING we imported our list of DE genes and reference lists for *qki* and JQ1 data sets as used for our PANTHER query described above. Every id list was then mapped to STRING identifiers using the map() function with argument: removeUnmappedRows = TRUE. The reference lists were set as background using set_background(). STRING enrichment analysis results were generated through subsequently calling the function get_enrichment() with arguments: category="Process","Component" or "Function"; methodMT='fdr'; iea='FALSE'. The output was exported to a table (.csv) file for each GO domain and each data set.

TopGO enrichment analysis
To apply topGO[10] on our data, we utilized the topGO (v2.32.0), org.Mm.eg.db (v3.6.0) and org.Hs.eg.db (v3.6.0) packages in R (v3.5.0) with a custom written Rmarkdown script based on the topGO vignette. As input to topGO we imported our list of DE genes and reference lists for *qki* and JQ1 data sets as used for our PANTHER query described above. The mouse id lists were mapped to ENTREZ identifiers using the MGI2EG() function from org.Mm.eg.db. From the reference and DE lists, a factor list alg was generated indicating for each gene whether is was classified as DE or not. This served as an input to generate a topGOdata object with the function new() with arguments: ontology = "BP","CC" or "MF"; allGenes=alg, nodeSize=1; annot=annFun.org; mapping="org.Mm.eg.db" (*qki*) or "org.Hs.eg.db" (JQ1); gene_id="entrez" (*qki*) or "ensembl" (JQ1). TopGO results object was generated by calling the functions runTest(), with arguments: topGOdata object; algorithm='elim'; statistic='fisher'. The results output was generated with function GenTable(), with arguments: topGOdata object; Fisher.elim=topGO results object; orderBy="Fisher.elim", topNodes= length of topGO results object (all GO nodes). The output was exported to a table (.csv) file for each GO domain and each data set. The topGO p-values were not corrected for multiple testing, so we performed Benjamini-Hochberg multiple testing correction in as described above for GeneWalk prior to the downstream comparison analysis.

Supplemental References
86. Montojo J, Zuberi K, Rodriguez H, Kazi F, Wright G, Donaldson SL, et al. GeneMANIA Cytoscape plugin: fast gene function predictions on the desktop. Bioinformatics. 2010;26:2927–8.

87. Bauer S, Robinson PN, Gagneur J. Model-based gene set analysis for Bioconductor. Bioinformatics. 2011;27:1882–3.
