# Supplementary material for: GeneWalk identifies relevant gene functions for a biological context using network representation learning
Source: Genome Biol. 2021 Feb 2;22:55. doi: 10.1186/s13059-021-02264-8 (PMC7852222; doi:10.1186/s13059-021-02264-8)
Supplement: Supplementary file 5 — Additional file 5. Review history. [file 13059_2021_2264_MOESM5_ESM.docx]

Review History

**First round of review**

**Reviewer 1**

**Were you able to assess all statistics in the manuscript, including the appropriateness of statistical tests used?**

Yes, and I have assessed the statistics in my report.

**Comments to author:**

Summary

The problem that appears to be tackled here is the identification of the specific annotations of a gene that may be more relevant to a given experiment. Authors outline a new approach to ranking the GO terms associated to a gene by their importance in the given experiment. This is done by systematically constructing an associated gene network based on a curated knowledge base derived from the scientific literature. Authors argue that this network provides an experimental context within which annotations are made, and that local network relationships allow GO annotations to be more narrowly focused on that context.

Originality

Unlike other methods GeneWalk constructs a "experiment-specific" gene network through the use of one of two knowledge bases (INDRA or Pathway Commons). This added component allows GeneWalk to evaluate the significance of GO annotations with respect to an experimental context (i.e. local regulatory relationships in the constructed network) versus a global set of differentially expressed genes.

Validity

Authors go to great lengths to try to validate their approach with respect to two "ground truth" datasets derived from previous studies: Qki deletion in brain myelination (RNA-Seq), and the human T-cell acute lymphoblastic leukemia cell line response to treatment with JQ1 (NET-seq). Authors explore alternative methods, as well as alternative configurations of their own architecture. However, the results presented do not provide convincing evidence for the validity of the method at this point.

Recommendation

Major revision

Critical Notes

*	I would recommend given a brief overview of the pathway and GO analysis areas in the introduction. There are plenty of review papers that would be useful here to outline the context of this work. Some comprehensive reviews and even comparative assessments of such methods are now available.

*	The introduction should be revised. The problem is not very well described. The problem definition should include the reasons for proposing this type of analysis, the input to this analysis, the output obtained from it. The reason appears to be the desire to have the ability to rank which of the GO annotations associated to a gene would be more relevant for the given experiment. This appears to be a intellectually reasonable goal but it is not clear why this is useful and how this ranking could be used further. What problem does this solve? For instance, the goal of the GO enrichment analysis is to identify the GO terms (e.g. biological processes) that are important in the given experiment. This is important because one needs to understand the underlying biological processes. This information can be then used to design other experiments to validate whether these processes are truly implicated, develop a mechanistic understanding of what is happening, etc. What is the equivalent here? Assuming that each gene is associated with a ranked list of GO terms, how is this information going to be used? Note that the significant GO terms for the given experiment are already known from the GO enrichment analysis and/or pathway analysis. Also, the association between the given gene and the GO terms is also previously known. How can this ranking be used? If one has two different experiment in which the same genes has a differently ranked list of GO terms, what can one learn from this difference in ranking? How is the approach proposed here going to be useful?

*	It is not clear how the comparison methods have been chosen. For instance, one of the methods chosen is GGEA. This is a rather obscure method with only 63 citations in 11 years since publications.

*	The validation is not clear at all. On page 27, line 755, it is stated that the authors used "systematic manual text mining". However, a few lines later, page 28, line 760, they state that biological context terms … "can represent characteristic parts of words to ensure GO annotations are found with regular expression". Which one is it then? It's either a systematic manual text mining or software analysis using regular expressions, it cannot be both.

*	It is not clear how the rank order for the ground truth was established. Given that the superiority of the proposed method is based entirely on the claim that the ranking yielded by the method is more similar to the ranking of the ground truth, this is a crucial point.

*	The so-called "ground truth" is very questionable. For instance, for the gene Mal, the authors chose the "biological context" as being "myelin". They say that "we searched all GO annotation of that particular DE gene for presence of the respective biological context term and assigned the labels and ranks as described above for biological context term 'myelin'." This is not reasonable. I analyzed the data from this particular experiment and the most significant biological processes (BPs) produced with ORA were "ensheathment of neurons", "axon ensheatment" and "myelination". Note that these are actually related terms: myelination is a type of axon ensheatment which is a type of ensheathment of neurons. They are all correct and extremely relevant. There is absolutely no reasons to only consider the terms that contain "myelin" and not the others.

*	The (mean-)padj seems very unclear and needs to be explained. I initially thought this notation represents the mean of the adjusted p-values. However, later, I have seen a statement "we classified cases of (mean-)padj > 1" (line 774). It is not clear how the mean of p-values (which should all be< 1) can be greater than 1. So I have no idea what was done here.

*	The "ground truth" data sets rely on mining the literature to set up the truth. The obvious question is as follow: since INDRA has mined the same literature, your ground truth is already baked into the GWN you've constructed to validate GeneWalk. This may or may not be true, but it needs a lot more discussion since both rely on text mining and the details of INDRA are rather opaque in this paper. At this time, this appears to be a very strong weakness of this paper since all the superiority of the proposed method may come from this information cross-contamination.

*	The thoroughness of the work behind Figure S2 C is commendable, but, given that this is done with respect to one study (Qki), this appears to be a case of overfitting the architecture to optimize performance. |There is a need for a thorough discussion as to why the version chosen is optimal for all studies, or some discussion for when alternative versions of GeneWalk may be more suitable (e.g., are some of these alternatives equivalent to hyperparameters?)

*	There is a need for some figure (modify Fig1A?) indicating what we're getting from INDRA. I know what GO terms look like, but have no experience with INDRA.

*	221

◦	"PC" used for Pathway Commons, though the abbreviation isn't introduced until 520

*	654 - 752 Editor

◦	though the detail is much appreciated, would these Methods for competing software be better suited as Supplementary Methods?

**Reviewer 2**

**Were you able to assess all statistics in the manuscript, including the appropriateness of statistical tests used?**

Yes, and I have assessed the statistics in my report.

**Comments to author:**

Ietswaart et al. present a new approach, called GeneWalk, to identify the functions (GO terms, specifically) that are relevant in a given context, e.g. in a differential gene expression setting. The motivation is that, among different functions known for a particular gene, one would want to identify those that are specifically relevant in the context of an experiment. GeneWalk addresses this issue by building a network of genes that, for example, are differentially expressed in a specific experiment, along with their GO terms, and then identifies gene-GO term associations that have high similarity in a low-dimensional embedding of the network.

The topic is definitely of broad interest, given that finding relevant functions of the genes that are significant in a high-throughput experiment is a key step in most analytical settings. The manuscript is well written in my opinion, and the results are interesting. The algorithm is described in detail, is logical and justified, and appears to perform well, at least to the extent that the benchmarking datasets show. I also applaud the authors for providing a detailed documentation of the code (linked in the GitHub page).

My comments below are mostly with respect to more accurate representation of current approaches as well as the evaluation of their performance in comparison to GeneWalk:

1. On page 2, the manuscripts says "Thus the challenge lies in prioritizing those genes worthy of further study and identifying their most pertinent functions for the particular biological context. […] GO and gene set enrichment analysis (GSEA)[1,3-5,9-15] can be used to reveal which biological processes are enriched under each specific condition, but do not address the context-specific functions of individual genes". However, in reality, GSEA and other pathway enrichment approaches do provide a method for finding context-specific functions of specific genes. For example, if a specific GO term is enriched among differentially expressed genes using any enrichment analysis approach (e.g. GSEA), that GO term would be the context-specific function of its associated genes in that particular experiment. In fact, GSEA explicitly creates a "leading gene set" for each enriched pathway, providing a list of "genes worthy of further study" and "their most pertinent functions". Therefore, in my opinion GeneWalk does not do something that is conceptually different from existing approaches. The main focus should be therefore on whether GeneWalk works better than existing methods (which appears to be the case). I'd like to mention that I do understand that GeneWalk does not require a pathway to be enriched in order to identify it as a context-specific function of a gene, but that seems more of a difference in methodology rather than in concept.

This inaccurate interpretation of current methods also appears in the abstract and gets repeated throughout the manuscript. For example, in the abstract: "For individual genes, GO annotations are static and biological context can only be added by manual literature searches". However, as I mentioned above, current methods do add biological contexts. I believe the manuscript text should be revised to better reflect this fact.

2. The authors use DeepWalk to obtain an embedding of their gene/GO network, and then use the weights learned by DeepWalk to calculate the similarity between each pair of a gene and a GO term. Can the authors please compare this method to other approaches for network embedding, such as methods based on matrix factorization?

3. In the methods section, it is indicated that "to correct for multiple GO annotation testing for a given gene, we utilized the Benjamini-Hochberg FDR adjusted p-value". Do I understand correctly that FDR adjustment was done for each individual gene separately? In that case, I believe this procedure is incorrect, and the FDR adjustment should be applied to all the tested gene-GO term associations at the same time (what GeneWalk examines is whether, among all the gene-GO term associations, there are significant associations in a given context).

4. The authors start exploring the results of GeneWalk by focusing on three genes, Mal, Pllp, Plp1, in order to identify the context-specific functions of these genes after Qki deletion in mouse oligodendrocytes. They later present a more systematic evaluation of GeneWalk performance in this context; however, it is not clear how those three initial genes were selected. Can the authors please clarify?

5. I found it difficult to parse Additional File 3, which contains the benchmarking data. Could the authors please add more details and information?

**Authors Response**

**Point-by-point responses to the reviewers’ comments:**

*Response to reviewers*

*We thank the reviewers for their positive comments and constructive critiques. In our revised manuscript, we now include many additional analyses, including:*

*1. Benchmark performance assessments to extended ground truth based on parental GO terms*

*2. Comparison analysis of GeneWalk (INDRA) without network edges originating from the ground truth publications.*

*3. Comparison of GeneWalk to a matrix factorization network embedding method.*

*In addition, we made several changes to the text (marked in blue) to describe these results and to increase clarity in response to reviewer comments.*

Reviewer #1:

Summary

The problem that appears to be tackled here is the identification of the specific annotations of a gene that may be more relevant to a given experiment. Authors outline a new approach to ranking the GO terms associated to a gene by their importance in the given experiment. This is done by systematically constructing an associated gene network based on a curated knowledge base derived from the scientific literature. Authors argue that this network provides an experimental context within which annotations are made, and that local network relationships allow GO annotations to be more narrowly focused on that context.

Originality

Unlike other methods GeneWalk constructs a "experiment-specific" gene network through the use of one of two knowledge bases (INDRA or Pathway Commons). This added component allows GeneWalk to evaluate the significance of GO annotations with respect to an experimental context (i.e. local regulatory relationships in the constructed network) versus a global set of differentially expressed genes.

Validity

Authors go to great lengths to try to validate their approach with respect to two "ground truth" datasets derived from previous studies: Qki deletion in brain myelination (RNA-Seq), and the human T-cell acute lymphoblastic leukemia cell line response to treatment with JQ1 (NET-seq). Authors explore alternative methods, as well as alternative configurations of their own architecture. However, the results presented do not provide convincing evidence for the validity of the method at this point.

*Response: We thank the reviewer for the thoughtful and constructive comments. Below we address the concerns point-by-point.*

Recommendation

Major revision

Critical Notes

1* I would recommend given a brief overview of the pathway and GO analysis areas in the introduction. There are plenty of review papers that would be useful here to outline the context of this work. Some comprehensive reviews and even comparative assessments of such methods are now available.

*Response: Per reviewer’s request, we have expanded our overview and referencing of existing GO / pathway analyses and existing comparative assessments in the Background (introduction) section (line 53-64, p2-3).*

2* The introduction should be revised. The problem is not very well described. The problem definition should include the reasons for proposing this type of analysis, the input to this analysis, the output obtained from it. The reason appears to be the desire to have the ability to rank which of the GO annotations associated to a gene would be more relevant for the given experiment. This appears to be a intellectually reasonable goal but it is not clear why this is useful and how this ranking could be used further. What problem does this solve? For instance, the goal of the GO enrichment analysis is to identify the GO terms (e.g. biological processes) that are important in the given experiment. This is important because one needs to understand the underlying biological processes. This information can be then used to design other experiments to validate whether these processes are truly implicated, develop a mechanistic understanding of what is happening, etc. What is the

equivalent here? Assuming that each gene is associated with a ranked list of GO terms, how is this information going to be used? Note that the significant GO terms for the given experiment are already known from the GO enrichment analysis and/or pathway analysis. Also, the association between the given gene and the GO terms is also previously known. How can this ranking be used? If one has two different experiment in which the same genes has a differently ranked list of GO terms, what can one learn from this difference in ranking? How is the approach proposed here going to be useful?

*Response: We have revised the Background section (line 42-90, p2-4) to clarify (1) the problem description, (2) the reasons for proposing this type of analysis, (3) the input of GeneWalk, (4) the output of GeneWalk, (5) how this information can be used and (6) the similarities and distinctions between GeneWalk and GO enrichment analyses. We have illustrated these points with an example on EGFR.*

3* It is not clear how the comparison methods have been chosen. For instance, one of the methods chosen is GGEA. This is a rather obscure method with only 63 citations in 11 years since publications.

*Response: The alternative methods were chosen based on prevalence of usage or characteristic model features that we describe in Table 1. GGEA was chosen because it also incorporates a gene-gene interaction network besides GO annotations. We now mention this reasoning in the Background section where we introduce GO enrichment based methods (line 57, p2).*

4* The validation is not clear at all. On page 27, line 755, it is stated that the authors used "systematic manual text mining". However, a few lines later, page 28, line 760, they state that biological context terms … "can represent characteristic parts of words to ensure GO annotations are found with regular expression". Which one is it then? It's either a systematic manual text mining or software analysis using regular expressions, it cannot be both.

*Response: We have clarified our approach (line 740-763, p27-28). Through systematic, manual text mining, we first tabulated all mentioned genes and the associated terms describing their role in the particular experimental context (biological context term). These pairs are then further analyzed using regular expression analysis to find any GO annotations corresponding to each biological context term. We used regular expression analysis at this step, because GO terms are very particular and an exact string match in the text of the QKI publication is not always feasible.*

5* It is not clear how the rank order for the ground truth was established. Given that the superiority of the proposed method is based entirely on the claim that the ranking yielded by the method is more similar to the ranking of the ground truth, this is a crucial point.

*Response: In the results section, we have now specified how the ground truth rank ordering was determined for the three myelin-related genes, Mal, Plp1 and Pllp (line 208-210, p8) and also for the ground truth benchmark genes (line 230-231, p9).*

*We previously described the rank order comparison procedure in full in the methods section “Systematic comparison of GeneWalk with alternative methods” (line 795-806, p29). To more clearly bring the ground truth rank order establishment to the attention of the reader, we now also describe this for the ground truth benchmark genes in the “Context-specific gene-function benchmarks” methods section (line 757-763, p28) and referred to the following systematic comparison section for completeness.*

6* The so-called "ground truth" is very questionable. For instance, for the gene Mal, the authors chose the "biological context" as being "myelin". They say that "we searched all GO annotation of that particular DE gene for presence of the respective biological context term and assigned the labels and ranks as described above for biological context term 'myelin'." This is not reasonable. I analyzed the data from this particular experiment and the most significant biological processes (BPs) produced with ORA were "ensheathment of neurons", "axon ensheatment" and "myelination". Note that these are actually related terms: myelination is a type of axon ensheatment which is a type of ensheathment of neurons. They are all correct and extremely relevant. There is absolutely no reasons to only consider the terms that contain "myelin" and not the others.

*Response: As the reviewer indicated, myelination, axon ensheathment and ensheathment of neurons are in the GO ontology parentally related via an “is_a” relation. To address the reviewer’s concern, we have now performed two additional benchmark tests which include these related GO terms in a systematic and unbiased manner.*

*First, we assigned rank 1 to GO terms that are related through one or more “is_a” relations (for example axon ensheathment and ensheathment of neurons) to any direct GO annotation that was found in the ground truth publication (for instance myelination, which also has relevance rank 1). With this extended ground truth, we performed the benchmark comparison of GeneWalk and other methods (QKI: line 256-265, p10, Fig S1H, JQ1: line 397-401, p15, Figure S3H).The performance results between GeneWalk and all methods remain nearly indistinguishable from the original ground truth analysis (compare Figure S1H, S3H with Figure 3D and 4E respectively). This is mostly because the vast majority of genes (e.g. Mal ) do not actually have parental GO terms such as axon ensheathment as a direct GO annotation (for verification: Mal GO annotations are listed in Figure 2C). GO enrichment analyses also assign genes to the gene sets of such parental (indirect) GO annotations through the parental transitivity property, as now discussed (line 538-544, p20). This transitivity is useful to find globally enriched processes but it is not useful for GeneWalk’s objective (line 297-300, p11 for a GeneWalk test version with transitivity).*

*To further address the reviewer’s concern and enable more inclusion of the indirect GO annotations that are significantly enriched according to alternative methods, we performed an additional ground truth benchmark comparison analysis (QKI: line 256-265, p10, Fig S1I, JQ1: line 397-401, p15, Figure S3I). Here, we propagated the significant p-adjust values from these parental GO terms to all their children. Direct GO annotations that were themselves not enriched, now become significant results if one (or more) of their parents GO terms were. We then compared the performance of these “parentally enhanced” alternative methods, alongside GeneWalk, whose results remained largely unaltered with this procedure, to the original ground truth ranking benchmark test. We show that also in this case, the conclusions remain unaltered: GeneWalk outperforms the alternative methods (line 256-265, p10).*

*We thank the reviewer for suggesting these new ground truth analyses. The overall conclusion is that enriched indirectly related GO terms do not matter for the problem that GeneWalk solves: to rank order the relevance of a gene’s (direct) GO annotations. We have now mentioned this conclusion in our results section (line 263-265, p10).*

7* The (mean-)padj seems very unclear and needs to be explained. I initially thought this notation represents the mean of the adjusted p-values. However, later, I have seen a statement "we classified cases of (mean-)padj > 1" (line 774). It is not clear how the mean of p-values (which should all be< 1) can be greater than 1. So I have no idea what was done here.

*Response: First, our apologies, we had made a typographical error in the case “we classified cases of (mean-)padj > 1”. We have now corrected this to “we classified cases with (mean-)padj >= 0.1” (line 773, p28), in correspondence with the rest of the text.*

*Furthermore, we understand how our notation could cause confusion. We have now clarified that (mean-)padj indicates a shortened notation for two types of output values: mean-padj in the case of GeneWalk and padj for all alternative methods (line 771-772, p28). These outputs are directly comparable between methods and treated the same for all downstream comparison analyses.*

8* The "ground truth" data sets rely on mining the literature to set up the truth. The obvious question is as follow: since INDRA has mined the same literature, your ground truth is already baked into the GWN you've constructed to validate GeneWalk. This may or may not be true, but it needs a lot more discussion since both rely on text mining and the details of INDRA are rather opaque in this paper. At this time, this appears to be a very strong weakness of this paper since all the superiority of the proposed method may come from this information cross-contamination.

*Response: The reviewer makes a fair point. To address the concern, we first found that the INDRA literature mining of the ground-truth publications resulted in 3 (QKI) and 10 (JQ1) reaction statements that form edges in the respective GWNs. Next, we ran GeneWalk without these edges and showed that the benchmark GeneWalk results remain largely unaltered and GeneWalk overall still outperforms all alternative methods. This result can be intuitively understood because the combination of (1) leveraging all publications results in a very small fraction of omitted edges and (2) GeneWalk’s use of random walks, which makes it relatively insensitive to loss of individual edges to sample the neighborhood. We now include these control simulations in our results section (QKI: line 250-254, p9-10 and JQ1: line 397-401, p15) and Figure S1G and S3G.*

9* The thoroughness of the work behind Figure S2 C is commendable, but, given that this is done with respect to one study (Qki), this appears to be a case of overfitting the architecture to optimize performance. |There is a need for a thorough discussion as to why the version chosen is optimal for all studies, or some discussion for when alternative versions of GeneWalk may be more suitable (e.g., are some of these alternatives equivalent to hyperparameters?)

*Response: We thank the reviewer for the appreciation of our qki model robustness analysis results in Figure S2C. We have now included Figure S2F where we show the equivalent results for the JQ1 study. Altogether, these results show that the robustness conclusions also hold for the JQ1 study and that the GeneWalk architecture is not overfitted to the qki results.*

*As suggested, we have now included more discussion (line 535-567, p20-21) on why the GeneWalk model is generally appropriate. Possible alternative model architectures / hyperparameters settings depending on future use cases are also mentioned.*

10* There is a need for some figure (modify Fig1A?) indicating what we're getting from INDRA. I know what GO terms look like, but have no experience with INDRA.

*Response: We have added a new schematic (Figure 1B) which explains in more detail how the gene network is assembled from the INDRA or Pathway Commons knowledge base. This new schematic visualizes that INDRA provides reaction statements mined from automated literature reading and pathway database queries (line 79-82, p3 and line 960-957, p36) on the genes of interest. These reaction statements are then used as edges in the GeneWalk network.*

11* 221 "PC" used for Pathway Commons, though the abbreviation isn't introduced until 520

*Response: We now introduce the abbreviation PC for Pathway Commons in our results section (line 186, p7).*

12* 654 - 752 Editor ◦ though the detail is much appreciated, would these Methods for competing software be better suited as Supplementary Methods?

*Response: In line with the suggestion, we have moved the indicated methods of the alternative methods to the Additional file 1: Supplemental Methods section. We also refer to these supplemental methods on line 766-768 (p28), where the comparison analysis is explained in detail.*

Reviewer #2: Ietswaart et al. present a new approach, called GeneWalk, to identify the functions (GO terms, specifically) that are relevant in a given context, e.g. in a differential gene expression setting. The motivation is that, among different functions known for a particular gene, one would want to identify those that are specifically relevant in the context of an experiment. GeneWalk addresses this issue by building a network of genes that, for example, are differentially expressed in a specific experiment, along with their GO terms, and then identifies gene-GO term associations that have high similarity in a low-dimensional embedding of the network.

The topic is definitely of broad interest, given that finding relevant functions of the genes that are significant in a high-throughput experiment is a key step in most analytical settings. The manuscript is well written in my opinion, and the results are interesting. The algorithm is described in detail, is logical and justified, and appears to perform well, at least to the extent that the benchmarking datasets show. I also applaud the authors for providing a detailed documentation of the code (linked in the GitHub page).

My comments below are mostly with respect to more accurate representation of current approaches as well as the evaluation of their performance in comparison to GeneWalk:

*Response: We thank the reviewer for the positive feedback and interest. Below we address the particular comments.*

*1. On page 2, the manuscripts says "Thus the challenge lies in prioritizing those genes worthy of further study and identifying their most pertinent functions for the particular biological context. […] GO and gene set enrichment analysis (GSEA)[1,3-5,9-15] can be used to reveal which biological processes are enriched under each specific condition, but do not address the context-specific functions of individual genes". However, in reality, GSEA and other pathway enrichment approaches do provide a method for finding context-specific functions of specific genes. For example, if a specific GO term is enriched among differentially expressed genes using any enrichment analysis approach (e.g. GSEA), that GO term would be the context-specific function of its associated genes in that particular experiment. In fact, GSEA explicitly creates a "leading gene set" for each enriched pathway, providing a list of "genes worthy of further study" and "their most pertinent functions".*

*Therefore, in my opinion GeneWalk does not do something that is conceptually different from existing approaches. The main focus should be therefore on whether GeneWalk works better than existing methods (which appears to be the case). I'd like to mention that I do understand that GeneWalk does not require a pathway to be enriched in order to identify it as a context-specific function of a gene, but that seems more of a difference in methodology rather than in concept.*

This inaccurate interpretation of current methods also appears in the abstract and gets repeated throughout the manuscript. For example, in the abstract: "For individual genes, GO annotations are static and biological context can only be added by manual literature searches". However, as I mentioned above, current methods do add biological contexts. I believe the manuscript text should be revised to better reflect this fact.

*Response: In accordance with the reviewer’s argument, we have now mentioned in the Background (introduction) section (line 59-60, p3) that enrichment-based methods, such as GSEA, can be used to generate gene-specific information. Furthermore, in the abstract we have deleted the sentence mentioned by the reviewer for accuracy.*

2. The authors use DeepWalk to obtain an embedding of their gene/GO network, and then use the weights learned by DeepWalk to calculate the similarity between each pair of a gene and a GO term. Can the authors please compare this method to other approaches for network embedding, such as methods based on matrix factorization?

*Response: We compared DeepWalk directly to Node2vec, a different network embedding method, albeit also based on random walks. We demonstrated that Node2vec does not improve the overall performance of GeneWalk as compared to using DeepWalk for the network embeddings (line 304-307, p11).*

*DeepWalk is mathematically equivalent to a spectral decomposition of the network’s normalized Laplacian matrix in the limit of an infinite random walk length (ref 41: Qiu et al. WSDM’18). So to investigate how performance changes using a method based on matrix factorization, we now include results (line 308-311, p11 and Fig S2) on a modified version of GeneWalk with a long random walk length (L=1000) that performs less well than a GeneWalk with short walks (L = 10 steps). This modification approximates the infinite walk length case and (through mathematical equivalence) a spectral decomposition matrix factorization. Lastly, in the Discussion section, we now refer to several matrix factorization based methods that were previously developed for various biological tasks (line 546-548, p20).*

3. In the methods section, it is indicated that "to correct for multiple GO annotation testing for a given gene, we utilized the Benjamini-Hochberg FDR adjusted p-value". Do I understand correctly that FDR adjustment was done for each individual gene separately? In that case, I believe this procedure is incorrect, and the FDR adjustment should be applied to all the tested gene-GO term associations at the same time (what GeneWalk examines is whether, among all the gene-GO term associations, there are significant associations in a given context).

*Response: The reviewer has correctly understood that the FDR adjustment is applied for each gene individually. We believe this method is appropriate because our methodology is gene-specific. So for any given gene of interest that the end user chooses to look at, GeneWalk provides the FDR adjusted p-values of its GO annotations. If the FDR adjustment would be applied over all gene-GO term associations, this would address a different question: given all genes and their functions, what are the most relevant pairs in the context. This would then be a global comparison across all genes and no longer a gene-specific comparison. We hope that these clarifications resolve this concern of the reviewer.*

4. The authors start exploring the results of GeneWalk by focusing on three genes, Mal, Pllp, Plp1, in order to identify the context-specific functions of these genes after Qki deletion in mouse oligodendrocytes. They later present a more systematic evaluation of GeneWalk performance in this context; however, it is not clear how those three initial genes were selected. Can the authors please clarify?

*Response: We have now clarified why these three initial genes were selected (line 177-180, p7). They were the three most strongly downregulated genes that were also previously known to be involved in myelination. In fact none of the other more differentially expressed genes have any GO annotations with the word myelin present (see Additional file 2, DE genes are ordered by differential expression strength in descending order).*

5. I found it difficult to parse Additional File 3, which contains the benchmarking data. Could the authors please add more details and information?

*Response: We have added more detail on the contents of Additional File 3 in the main text section Additional files (line 1125-1137, p44), where this file is introduced. There, we now refer back to the methods section Context-specific gene-function benchmarks that describes the contents and generation of Additional File 3 in detail.*

**Second round of review**

**Reviewer 1**

Response to reviewers

We thank the reviewers for their positive comments and constructive critiques. In our revised manuscript, we now include many additional analyses, including:

1.	Benchmark performance assessments to extended ground truth based on parental GO terms

2.	Comparison analysis of GeneWalk (INDRA) without network edges originating from the ground truth publications.

3.	Comparison of GeneWalk to a matrix factorization network embedding method.

In addition, we made several changes to the text (marked in blue) to describe these results and to increase clarity in response to reviewer comments.

Reviewer #1:

Summary

The problem that appears to be tackled here is the identification of the specific annotations of a gene that may be more relevant to a given experiment. Authors outline a new approach to ranking the GO terms associated to a gene by their importance in the given experiment. This is done by systematically constructing an associated gene network based on a curated knowledge base derived from the scientific literature. Authors argue that this network provides an experimental context within which annotations are made, and that local network relationships allow GO annotations to be more narrowly focused on that context.

Originality

Unlike other methods GeneWalk constructs a "experiment-specific" gene network through the use of one of two knowledge bases (INDRA or Pathway Commons). This added component allows GeneWalk to evaluate the significance of GO annotations with respect to an experimental context (i.e. local regulatory relationships in the constructed network) versus a global set of differentially expressed genes.

Validity

Authors go to great lengths to try to validate their approach with respect to two "ground truth" datasets derived from previous studies: Qki deletion in brain myelination (RNA-Seq), and the human T-cell acute lymphoblastic leukemia cell line response to treatment with JQ1 (NET-seq). Authors explore alternative methods, as well as alternative configurations of their own architecture. However, the results presented do not provide convincing evidence for the validity of the method at this point.

Response: We thank the reviewer for the thoughtful and constructive comments. Below we address the concerns point-by-point.

Recommendation

Major revision

Critical Notes

1*	I would recommend given a brief overview of the pathway and GO analysis areas in the introduction. There are plenty of review papers that would be useful here to outline the context of

this work. Some comprehensive reviews and even comparative assessments of such methods are now available.

Response: Per reviewer’s request, we have expanded our overview and referencing of existing GO / pathway analyses and existing comparative assessments in the Background (introduction) section (line 53-64, p2-3).

We appreciate the intention to address our comments, but this part still needs to be improved. The authors cited 2 papers as reviews:

A critical assessment of Mus musculusgene function prediction using integrated genomic evidence

<https://dx.doi.org/10.1186/gb-2008-9-s1-s2>

and

Toward a gold standard for benchmarking gene set enrichment analysis.

<http://dx.doi.org/10.1093/bib/bbz158>

The first one is actually completely off target in this context. This paper presents the result of a competition to predict functions for mouse genes and has nothing to do with pathway analysis or gene set analysis. The input of the methods described in the cited paper is a gene, and the output is a prediction of that gene’s function. This can be seen as somewhat similar to what GeneWalk is trying to accomplish (associate a function to a gene) but the GeneWalk approach is actually based on GO and existing annotations (i.e. picking the meaningful ones from a set of existing GO annotations), rather than developing a classifier able to predict _new_ functions, as in this reference. There is really no connection.

Here are some review papers that would be more appropriate for GO and pathway analysis:

Identifying significantly impacted pathways: a comprehensive review and assessment <https://dx.doi.org/10.1186/s13059-019-1790-4>

Comparative study on gene set and pathway topology-based enrichment methods <https://dx.doi.org/10.1186/s12859-015-0751-5>

Methods and approaches in the topology-based analysis of biological pathways <https://dx.doi.org/10.3389/fphys.2013.00278>

The second paper cited by the authors is appropriate and recent but covers only a small category of methods, the gene set analysis methods. It is important to distinguish between gene set analysis methods and pathway analysis methods. The manuscript mentions both in an almost interchangeable way which can be confusing. A gene set is a set of genes, ie. an unordered collection of genes and nothing more. A pathway is a graph. A pathway includes a set of genes, but also a set of directed edges of various types (e.g. activation, repression, etc.). Performing a gene set analysis means relying solely on the set of genes and ignoring the topology of the pathway, all the edges, their types, their directions, and all the phenomena that are described by this topology. Performing a pathway analysis method involves trying to leverage the topology and all the information contained therein. A discussion of the differences between gene set analysis and pathway analysis is made very clearly in the literature, for instance in <https://dx.doi.org/10.3389/fphys.2013.00278> which actually includes a figure illustrating the

difference between MAPK seen as a gene set and the same seen as a pathway. Given a pathway, one can always construct a corresponding gene set by eliminating all the edges. The reverse, however, is not possible. Given a gene set only, the corresponding pathway can never be reconstructed from the gene set alone.

In order to address this point, the author should differentiate between gene sets and pathways, as the objects being analyzed and making the points above. Also, the authors should include the proper definition of the 3 main classes of analysis methods. These are most often described as: - Enrichment analysis (or overrepresentation or ORA) such as the hypergeometric approach was proposed as an approach by Tavazoie et al 1999 ([https://dx.doi.org/10.1038/10343](https://dx.doi.org/10.1038/10343))) and as a tool in 2002 ([https://dx.doi.org/10.1016/s0888-7543(02)00021-6](https://dx.doi.org/10.1016/s0888-7543(02)00021-6,), ). ORA can only analyze gene sets. - Functional class scoring (FCS) such as the Gene Set Enrichment Analysis (GSEA) proposed by Subramanian et al ([https://dx.doi.org/10.1073/pnas.0506580102](https://dx.doi.org/10.1073/pnas.0506580102))) in 2005. FCS methods only analyze gene sets, as well.

- The topological analysis approach (or topology-based, or pathway analysis) such as the Impact Analysis was proposed by Draghici et al. ([https://dx.doi.org/10.1093/bioinformatics/btn577](https://dx.doi.org/10.1093/bioinformatics/btn577))) in 2007. Pathway analysis methods analyze pathways.

From the 3 categories above, it appears that GeneWalk uses enrichment but this should be made clear. Also, would GeneWalk be expected to do better if it were to use FCS or a topology-based approach? It would be worth discussing this briefly.

I would recommend revising the introduction to clarify the aspects above, as well as add the appropriate references.

2*	The introduction should be revised. The problem is not very well described. The problem definition should include the reasons for proposing this type of analysis, the input to this analysis, the output obtained from it. The reason appears to be the desire to have the ability to rank which of the GO annotations associated to a gene would be more relevant for the given experiment. This appears to be a intellectually reasonable goal but it is not clear why this is useful and how this ranking could be used further. What problem does this solve? For instance, the goal of the GO enrichment analysis is to identify the GO terms (e.g. biological processes) that are important in the given experiment. This is important because one needs to understand the underlying biological processes. This information can be then used to design other experiments to validate whether these processes are truly implicated, develop a mechanistic understanding of what is happening, etc. What is the

equivalent here? Assuming that each gene is associated with a ranked list of GO terms, how is this information going to be used? Note that the significant GO terms for the given experiment are already known from the GO enrichment analysis and/or pathway analysis. Also, the association between the given gene and the GO terms is also previously known. How can this ranking be used? If one has two different experiment in which the same genes has a differently ranked list of GO terms, what can one learn from this difference in ranking? How is the approach proposed here going to be useful?

Response: We have revised the Background section (line 42-90, p2-4) to clarify (1) the problem description, (2) the reasons for proposing this type of analysis, (3) the input of GeneWalk, (4) the

output of GeneWalk, (5) how this information can be used and (6) the similarities and distinctions between GeneWalk and GO enrichment analyses. We have illustrated these points with an example on EGFR.

This paragraph (lines 84-90) could still be significantly improved. For instance, it is stated that “GeneWalk automatically assembles a biological network from a knowledge based and GO ontology starting with a list of genes of interest (...) as input”. From here, it is clear that the input is a list of genes. However, according to this phrase, the output appears to be an “biological network” which is not the case. The assembly of this network is part of how GeneWalk works, not what is produced. The paragraph continues with “how” GW works (“the network structure is learned through...”) and what this representation enables, etc. It would be helpful for the reader if the authors stated clearly, the problem, the input, the output, and the utility of the method in a single place, without mixing in details of how the approach works. The “how” part is described in details in the rest of the paper.

Also, maybe a short paragraph could clarify and differences and similarities between GeneWalk and GO enrichment and/or pathway analysis. I think the point is stated clearly a few pages later in lines 268-271 but it would improve the paper if this were to be moved in the Background section when presenting GeneWalk at the general level.

3*	It is not clear how the comparison methods have been chosen. For instance, one of the methods chosen is GGEA. This is a rather obscure method with only 63 citations in 11 years since publications.

Response: The alternative methods were chosen based on prevalence of usage or characteristic model features that we describe in Table 1. GGEA was chosen because it also incorporates a gene-gene interaction network besides GO annotations. We now mention this reasoning in the Background section where we introduce GO enrichment based methods (line 57, p2).

The changes are satisfactory.

4*	The validation is not clear at all. On page 27, line 755, it is stated that the authors used "systematic manual text mining". However, a few lines later, page 28, line 760, they state that biological context terms ... "can represent characteristic parts of words to ensure GO annotations are found with regular expression". Which one is it then? It's either a systematic manual text mining or software analysis using regular expressions, it cannot be both.

Response: We have clarified our approach (line 740-763, p27-28). Through systematic, manual text mining, we first tabulated all mentioned genes and the associated terms describing their role in the particular experimental context (biological context term). These pairs are then further analyzed using regular expression analysis to find any GO annotations corresponding to each biological context term. We used regular expression analysis at this step, because GO terms are very particular and an exact string match in the text of the QKI publication is not always feasible.

The changes are satisfactory.

5*	It is not clear how the rank order for the ground truth was established. Given that the superiority of the proposed method is based entirely on the claim that the ranking yielded by the method is more similar to the ranking of the ground truth, this is a crucial point.

Response: In the results section, we have now specified how the ground truth rank ordering was determined for the three myelin-related genes, *Mal*, *Plp1* and *Pllp* (line 208-210, p8) and also for the ground truth benchmark genes (line 230-231, p9).

We previously described the rank order comparison procedure in full in the methods section “*Systematic comparison of GeneWalk with alternative methods*” (line 795-806, p29). To more clearly bring the ground truth rank order establishment to the attention of the reader, we now also describe this for the ground truth benchmark genes in the “*Context-specific gene-function benchmarks*” methods section (line 757-763, p28) and referred to the following systematic comparison section for completeness.

The changes are satisfactory.

6*	The so-called "ground truth" is very questionable. For instance, for the gene Mal, the authors chose the "biological context" as being "myelin". They say that "we searched all GO annotation of that particular DE gene for presence of the respective biological context term and assigned the labels and ranks as described above for biological context term 'myelin'." This is not reasonable. I analyzed the data from this particular experiment and the most significant biological processes (BPs) produced with ORA were "ensheathment of neurons", "axon ensheatment" and "myelination". Note that these are actually related terms: myelination is a type of axon ensheatment which is a type of ensheathment of neurons. They are all correct and extremely relevant. There is absolutely no reason to only consider the terms that contain "myelin" and not the others.

Response: As the reviewer indicated, myelination, axon ensheathment and ensheathment of neurons are in the GO ontology parentally related via an “is_a” relation. To address the reviewer’s concern, we have now performed two additional benchmark tests which include these related GO terms in a systematic and unbiased manner.

First, we assigned rank 1 to GO terms that are related through one or more “is_a” relations (for example axon ensheathment and ensheathment of neurons) to any direct GO annotation that was found in the ground truth publication (for instance myelination, which also has relevance rank 1). With this extended ground truth, we performed the benchmark comparison of GeneWalk and other methods (QKI: line 256-265, p10, Fig S1H, JQ1: line 397-401, p15, Figure S3H).The performance results between GeneWalk and all methods remain nearly indistinguishable from the original ground truth analysis (compare Figure S1H, S3H with Figure 3D and 4E respectively). This is mostly because the vast majority of genes (e.g. *Mal* ) do not actually have parental GO terms such as axon ensheathment as a direct GO annotation (for verification: *Mal* GO annotations are listed in Figure 2C). GO enrichment analyses also assign genes to the gene sets of such parental (indirect) GO annotations through the parental transitivity property, as now discussed (line 538-544, p20). This transitivity is useful to find globally enriched processes but it is not useful for GeneWalk’s objective (line 297-300, p11 for a GeneWalk test version with transitivity).

To further address the reviewer’s concern and enable more inclusion of the indirect GO annotations that are significantly enriched according to alternative methods, we performed an additional ground truth benchmark comparison analysis (QKI: line 256-265, p10, Fig S1I, JQ1: line 397-401, p15, Figure S3I). Here, we propagated the significant p-adjust values from these parental GO terms to all their children. Direct GO annotations that were themselves not enriched, now become significant results if one (or more) of their parents GO terms were. We then compared the performance of these “parentally enhanced” alternative methods, alongside GeneWalk, whose results remained largely unaltered with this procedure, to the original ground truth ranking benchmark test. We show that also in this case, the conclusions remain unaltered: GeneWalk outperforms the alternative methods (line 256-265, p10).

We thank the reviewer for suggesting these new ground truth analyses. The overall conclusion is that enriched indirectly related GO terms do not matter for the problem that GeneWalk solves: to rank order the relevance of a gene’s (direct) GO annotations. We have now mentioned this conclusion in our results section (line 263-265, p10).

Thank you for the effort to address this comment. I understand what was done to address this point. However, I am not entirely convinced that this is the right thing to do. Propagating the p-values from the parents to the children does not make much sense to this reviewer since the children are more specific than the parents. If there is evidence that a process such as ensheathment of neurons is significant, propagating the p-values to the children would make them significant and there is no evidence that axon ensheathment for instance is now implicated in the phenotype as opposed to other type of (other children of) ensheathment of neurons. Also, there is no reason to believe that ALL children are meaningful, which is essentially what propagating the significant p-values downstream does. In essence, such downstream propagation of p-value is NOT expected to improve the results.

The net effect of the “is_a” relationships is that a given gene is associated to all the GO terms from the most specific term it is associated with, all the way up to the root. That means propagating genes from the children to the parents, not the other way around. When each GO term is considered, as in the GO analysis used here, this redundancy can influence the results of the classical approach in a negative way. This is perhaps related to the “moonlighting genes” mentioned earlier in the paper. However, this issue has been addressed in the literature by methods designed to eliminate cross-talk cause by the presence of the same genes in several biological processes or pathways (see Donato et al, Tomoioaga et al below). Also an even more appropriate thing to do is to simply down-weight the genes that are associated with many GO terms/pathways as proposed in Tarca et al. I believe that all these approaches came with associated R packages so I would suggest including one or more of these as comparable methods (Table 1) and include the results obtained with these in the validation.

Analysis and correction of crosstalk effects in pathway analysis.

<https://dx.doi.org/10.1101/gr.153551.112>

Pathway crosstalk effects: shrinkage and disentanglement using a Bayesian hierarchical model <https://dx.doi.org/10.1007/s12561-016-9160-1>

Down-weighting overlapping genes improves gene set analysis.

<https://dx.doi.org/10.1186/1471-2105-13-136>

7*	The (mean-)padj seems very unclear and needs to be explained. I initially thought this notation represents the mean of the adjusted p-values. However, later, I have seen a statement "we classified cases of (mean-)padj > 1" (line 774). It is not clear how the mean of p-values (which should all be< 1) can be greater than 1. So I have no idea what was done here.

Response: First, our apologies, we had made a typographical error in the case “we classified cases of (mean-)padj > 1”. We have now corrected this to “we classified cases with (mean-)padj >= 0.1” (line 773, p28), in correspondence with the rest of the text.

Furthermore, we understand how our notation could cause confusion. We have now clarified that (mean-)padj indicates a shortened notation for two types of output values: mean-padj in the case of GeneWalk and padj for all alternative methods (line 771-772, p28). These outputs are directly comparable between methods and treated the same for all downstream comparison analyses.

The changes are satisfactory.

8*	The "ground truth" data sets rely on mining the literature to set up the truth. The obvious question is as follow: since INDRA has mined the same literature, your ground truth is already baked into the GWN you've constructed to validate GeneWalk. This may or may not be true, but it needs a lot more discussion since both rely on text mining and the details of INDRA are rather opaque in this paper. At this time, this appears to be a very strong weakness of this paper since all the superiority of the proposed method may come from this information cross-contamination.

Response: The reviewer makes a fair point. To address the concern, we first found that the INDRA literature mining of the ground-truth publications resulted in 3 (QKI) and 10 (JQ1) reaction statements that form edges in the respective GWNs. Next, we ran GeneWalk without these edges and showed that the benchmark GeneWalk results remain largely unaltered and GeneWalk overall still outperforms all alternative methods. This result can be intuitively understood because the combination of (1) leveraging all publications results in a very small fraction of omitted edges and (2) GeneWalk’s use of random walks, which makes it relatively insensitive to loss of individual edges to sample the neighborhood. We now include these control simulations in our results section (QKI: line 250-254, p9-10 and JQ1: line 397-401, p15) and Figure S1G and S3G.

The changes are satisfactory.

9*	The thoroughness of the work behind Figure S2 C is commendable, but, given that this is done with respect to one study (Qki), this appears to be a case of overfitting the architecture to optimize performance. |There is a need for a thorough discussion as to why the version chosen is optimal for all studies, or some discussion for when alternative versions of GeneWalk may be more suitable (e.g., are some of these alternatives equivalent to hyperparameters?)

Response: We thank the reviewer for the appreciation of our *qki* model robustness analysis results in Figure S2C. We have now included Figure S2F where we show the equivalent results for the JQ1 study. Altogether, these results show that the robustness conclusions also hold for the JQ1 study and that the GeneWalk architecture is not overfitted to the *qki* results.

As suggested, we have now included more discussion (line 535-567, p20-21) on why the GeneWalk model is generally appropriate. Possible alternative model architectures / hyperparameters settings depending on future use cases are also mentioned.

The changes are satisfactory.

10*	There is a need for some figure (modify Fig1A?) indicating what we're getting from INDRA. I know what GO terms look like, but have no experience with INDRA.

Response: We have added a new schematic (Figure 1B) which explains in more detail how the gene network is assembled from the INDRA or Pathway Commons knowledge base. This new schematic visualizes that INDRA provides reaction statements mined from automated literature reading and pathway database queries (line 79-82, p3 and line 960-957, p36) on the genes of interest. These reaction statements are then used as edges in the GeneWalk network.

Thank you for your effort to address this. In Figure 1B, last panel, there is a network that involves blue nodes. The blue nodes have been defined above, in 1A, as being genes. However, the text (line 113) states that “we generated specific GWNs ... which generally resulted in fewer gene-gene and only gene-GO annotation connections”. This is very confusing. “only gene-GO annotations connections” would mean the figure should only contain edges between blue and red nodes. Please clarify what kinds of nodes are included in this context specific network at this stage and make Figure 1B consistent with that.

11*	221 "PC" used for Pathway Commons, though the abbreviation isn't introduced until 520

Response: We now introduce the abbreviation PC for Pathway Commons in our results section (line 186, p7).

The changes are satisfactory.

12*	654 - 752 Editor ◦ though the detail is much appreciated, would these Methods for competing software be better suited as Supplementary Methods?

Response: In line with the suggestion, we have moved the indicated methods of the alternative methods to the Additional file 1: Supplemental Methods section. We also refer to these supplemental methods on line 766-768 (p28), where the comparison analysis is explained in detail.

These changes are satisfactory.

However, in the section on Network representation (lines 116, 127), it is not clear how the network is trained. What is the input and what is the output? Is that an auto-encoder i.e. is the desired output for a given vector the same vector of pairs, just looking for a representation in a space with fewer dimensions? Or is the input vector the set of all inputs from the input-output pairs, and the desired output the set of all outputs from these pairs, looking to learn a model for the associations between inputs and outputs?

**Reviewer 2**

I thank the authors for addressing most of my concerns. However, one critical issue remains (about FDR adjustment) that requires revision of some of the results and figures.

The authors have confirmed that they have performed FDR correction for each gene separately, and have indicated in their response to my previous comment that “If the FDR adjustment would be applied over all gene-GO term associations, this would address a different question: given all genes and their functions, what are the most relevant pairs in the context”. However, this global analysis of all gene-GO term associations is in fact what the authors have done in many places in the manuscript, and therefore adjusting the p-value for each gene has resulted in the incorrect FDR. For example:

-	Page 17: “Using INDRA and Pathway Commons as the knowledge bases, we applied GeneWalk to these DE genes and found that 24% had at least one relevant GO term (FDR = 0.05, Additional file 2)”.

-	Figure 3F: “Boxplots of the GO term levels of all significant (p-adjust < 0.1) gene-GO annotation pairs across all qki DE genes present in the GWN.”

-	Figure 3H: “Hexagon density plot for all genes of interest (N=1861) in terms of number of connected GO terms and number of relevant GO terms (at FDR=0.05) …”.

-	Figure 4D: “The number of different unique GO annotations (y-axis) that are significant (p-adjust < 0.1) as a percentage of all unique GO annotation terms across all JQ1 DE genes present in the GWN”.

Please note that the above list is not an exhaustive list of the analyses that rely on this incorrect FDR calculation to look at global patterns across all gene-GO associations. As the authors have mentioned in their comment, the p-value adjustment process that is used in this version of the manuscript is *only* valid if there is a single pre-defined gene that is of interest and the user wants to know what terms are relevant to that one gene. In any other case, the current procedure for FDR calculation is invalid.

I emphasize this issue because not only it affects many of the analyses presented in this paper, but also it is highly prone to misuse and misinterpretation by future users. So, in addition to revising the results in this paper, the authors need to ensure that their package addresses this issue: my recommendation is that if the user has explicitly asked for relevant GO terms of one or a few pre-defined genes, then FDR correction should be done only to the gene-GO pairs involving those genes of interest. Otherwise, a global FDR correction should be provided to the user.

**Authors Response**

**Point-by-point responses to the reviewers’ comments:**

Response to reviewers

We appreciate the reviewers’ effort in reviewing our manuscript again and believe that it has resulted in a stronger paper. We respond below to comments below in purple.

Reviewer #1:

1* I would recommend given a brief overview of the pathway and GO analysis areas in the introduction. There are plenty of review papers that would be useful here to outline the context of this work. Some comprehensive reviews and even comparative assessments of such methods are now available.

Response: Per reviewer’s request, we have now expanded our overview and referencing of existing GO / pathway analyses and existing comparative assessments in the Background (introduction) section (line 53-64, p2-3).

We appreciate the intention to address our comments, but this part still needs to be improved. The authors cited 2 papers as reviews:

A critical assessment of Mus musculusgene function prediction using integrated genomic evidence https://dx.doi.org/10.1186/gb-2008-9-s1-s2

and

Toward a gold standard for benchmarking gene set enrichment analysis.

http://dx.doi.org/10.1093/bib/bbz158

The first one is actually completely off target in this context. This paper presents the result of a competition to predict functions for mouse genes and has nothing to do with pathway analysis or gene set analysis. The input of the methods described in the cited paper is a gene, and the output is a prediction of that gene’s function. This can be seen as somewhat similar to what GeneWalk is trying to accomplish (associate a function to a gene) but the GeneWalk approach is actually based on GO and existing annotations (i.e. picking the meaningful ones from a set of existing GO annotations), rather than developing a classifier able to predict _new_ functions, as in this reference. There is really no connection.

Here are some review papers that would be more appropriate for GO and pathway analysis:

Identifying significantly impacted pathways: a comprehensive review and assessment

https://dx.doi.org/10.1186/s13059-019-1790-4

Comparative study on gene set and pathway topology-based enrichment methods

https://dx.doi.org/10.1186/s12859-015-0751-5

Methods and approaches in the topology-based analysis of biological pathways

https://dx.doi.org/10.3389/fphys.2013.00278

The second paper cited by the authors is appropriate and recent but covers only a small category of methods, the gene set analysis methods. It is important to distinguish between gene set analysis methods and pathway analysis methods. The manuscript mentions both in an almost interchangeable way which can be confusing. A gene set is a set of genes, ie. an unordered collection of genes and nothing more. A pathway is a graph. A pathway includes a set of genes, but also a set of directed edges of various types (e.g. activation, repression, etc.). Performing a gene set analysis means relying solely on the set of genes and ignoring the topology of the pathway, all the edges, their types, their directions, and all the phenomena that are described by this topology. Performing a pathway analysis method involves trying to leverage the topology and all the information contained therein. A discussion of the differences between gene set analysis and pathway analysis is made very clearly in the literature, for instance in https://dx.doi.org/10.3389/fphys.2013.00278 which actually includes a figure illustrating the difference between MAPK seen as a gene set and the same seen as a pathway. Given a pathway, one can always construct a corresponding gene set by eliminating all the edges. The reverse, however, is not possible. Given a gene set only, the corresponding pathway can never be reconstructed from the gene set alone.

In order to address this point, the author should differentiate between gene sets and pathways, as the objects being analyzed and making the points above. Also, the authors should include the proper definition of the 3 main classes of analysis methods. These are most often described as:

- Enrichment analysis (or overrepresentation or ORA) such as the hypergeometric approach was proposed as an approach by Tavazoie et al 1999 (https://dx.doi.org/10.1038/10343) and as a tool in 2002 (https://dx.doi.org/10.1016/s0888-7543(02)00021-6, ). ORA can only analyze gene sets.

- Functional class scoring (FCS) such as the Gene Set Enrichment Analysis (GSEA) proposed by Subramanian et al (https://dx.doi.org/10.1073/pnas.0506580102) in 2005. FCS methods only analyze gene sets, as well.

- The topological analysis approach (or topology-based, or pathway analysis) such as the Impact Analysis was proposed by Draghici et al. (https://dx.doi.org/10.1093/bioinformatics/btn577) in 2007. Pathway analysis methods analyze pathways.

From the 3 categories above, it appears that GeneWalk uses enrichment but this should be made clear. Also, would GeneWalk be expected to do better if it were to use FCS or a topology-based approach? It would be worth discussing this briefly.

I would recommend revising the introduction to clarify the aspects above, as well as add the

appropriate references.

*Response: Based on the reviewer’s valuable suggestions, we have now updated our background section accordingly by introducing the difference between gene sets and pathways. We have included the three above described classes of analysis types (line 58-65, p2) and clarified that GeneWalk makes use of both the GO and gene network topology (line 94-98, p3). In addition, the key novel aspect is that GeneWalk is tailored towards function relevance prediction of individual genes, as opposed to enrichment analyses that aim to look for globally relevant GO terms (line 66-79, p2 and line 93, p3). This is enabled by network representation learning based on the GeneWalk network topology (line 96-98, p3). Furthermore, we have deleted the gene function prediction citation as it was deemed inappropriate and included all the references suggested here by the reviewer.*

2* The introduction should be revised. The problem is not very well described. The problem definition should include the reasons for proposing this type of analysis, the input to this analysis, the output obtained from it. The reason appears to be the desire to have the ability to rank which of the GO annotations associated to a gene would be more relevant for the given experiment. This appears to be a intellectually reasonable goal but it is not clear why this is useful and how this ranking could be used further. What problem does this solve? For instance, the goal of the GO enrichment analysis is to identify the GO terms (e.g. biological processes) that are important in the given experiment. This is important because one needs to understand the underlying biological processes. This information can be then used to design other experiments to validate whether these processes are truly implicated, develop a mechanistic understanding of what is happening, etc. What is the

equivalent here? Assuming that each gene is associated with a ranked list of GO terms, how is this information going to be used? Note that the significant GO terms for the given experiment are already known from the GO enrichment analysis and/or pathway analysis. Also, the association between the given gene and the GO terms is also previously known. How can this ranking be used? If one has two different experiment in which the same genes has a differently ranked list of GO terms, what can one learn from this difference in ranking? How is the approach proposed here going to be useful?

Response: We have revised the Background section (line 42-90, p2-4) to clarify (1) the problem description, (2) the reasons for proposing this type of analysis, (3) the input of GeneWalk, (4) the output of GeneWalk, (5) how this information can be used and (6) the similarities and distinctions between GeneWalk and GO enrichment analyses. We have illustrated these points with an example on EGFR.

This paragraph (lines 84-90) could still be significantly improved. For instance, it is stated that “GeneWalk automatically assembles a biological network from a knowledge based and GO ontology starting with a list of genes of interest (…) as input”. From here, it is clear that the input is a list of genes. However, according to this phrase, the output appears to be an “biological network” which is not the case. The assembly of this network is part of how GeneWalk works, not what is produced. The paragraph continues with “how” GW works (“the network structure is learned through…”) and what this representation enables, etc. It would be helpful for the reader if the authors stated clearly, the problem, the input, the output, and the utility of the method in a single place, without mixing in details of how the approach works. The “how” part is described in details in the rest of the paper.

*Response: We appreciate these suggestions to sharpen our introduction. We now repeat the problem statement in this paragraph (line 93-94, p3). We follow this by further clarifying that the biological network is not the output by adding the word “first” (line 94, p3). Thus, the problem statement (line 93-94, p3), input (line 94-96, p3), output (line 99-101, p3) and utility (line 101-106, p3) are clearly stated together within this paragraph. In addition, we believe that the reader benefits from our short “how” description (line 96-99, p3) in this paragraph as this provides a concise overview to help understand our method.*

Also, maybe a short paragraph could clarify and differences and similarities between GeneWalk and GO enrichment and/or pathway analysis. I think the point is stated clearly a few pages later in lines 268-271 but it would improve the paper if this were to be moved in the Background section when presenting GeneWalk at the general level.

*Response: In response to the reviewer’s previous point, we have expanded the background section to further detail the differences and similarities between GeneWalk, GO enrichment and pathway analysis in an earlier paragraph of the background section (line 54-79, p2). The points the reviewer refers to (“lines 268-271”) are also described in the background section already (line 55-58 and 66-79, p2). In order to avoid repetition within the same background section, we have chosen not to describe these differences again in a new paragraph.*

6* The so-called "ground truth" is very questionable. For instance, for the gene Mal, the authors chose the "biological context" as being "myelin". They say that "we searched all GO annotation of that particular DE gene for presence of the respective biological context term and assigned the labels and ranks as described above for biological context term 'myelin'." This is not reasonable. I analyzed the data from this particular experiment and the most significant biological processes (BPs) produced with ORA were "ensheathment of neurons", "axon ensheatment" and "myelination". Note that these are actually related terms: myelination is a type of axon ensheatment which is a type of ensheathment of neurons. They are all correct and extremely relevant. There is absolutely no reasons to only consider the terms that contain "myelin" and not the others.

Response: As the reviewer indicated, myelination, axon ensheathment and ensheathment of neurons are in the GO ontology parentally related via an “is_a” relation. To address the reviewer’s concern, we have now performed two additional benchmark tests which include these related GO terms in a systematic and unbiased manner.

First, we assigned rank 1 to GO terms that are related through one or more “is_a” relations (for example axon ensheathment and ensheathment of neurons) to any direct GO annotation that was found in the ground truth publication (for instance myelination, which also has relevance rank 1). With this extended ground truth, we performed the benchmark comparison of GeneWalk and other methods (QKI: line 256-265, p10, Fig S1H, JQ1: line 397-401, p15, Figure S3H). The performance results between GeneWalk and all methods remain nearly indistinguishable from the original ground truth analysis (compare Figure S1H, S3H with Figure 3D and 4E respectively). This is mostly because the vast majority of genes (e.g. Mal) do not actually have parental GO terms such as axon ensheathment as a direct GO annotation (for verification: Mal GO annotations are listed in Figure 2C). GO enrichment analyses also assign genes to the gene sets of such parental (indirect) GO annotations through the parental transitivity property, as now discussed (line 538-544, p20). This transitivity is useful to find globally enriched processes but it is not useful for GeneWalk’s objective (line 297-300, p11 for a GeneWalk test version with transitivity).

To further address the reviewer’s concern and enable more inclusion of the indirect GO annotations that are significantly enriched according to alternative methods, we performed an additional ground truth benchmark comparison analysis (QKI: line 256-265, p10, Fig S1I, JQ1: line 397-401, p15, Figure S3I). Here, we propagated the significant p-adjust values from these parental GO terms to all their children. Direct GO annotations that were themselves not enriched, now become significant results if one (or more) of their parents’ GO terms were. We then compared the performance of these “parentally enhanced” alternative methods, alongside GeneWalk, whose results remained largely unaltered with this procedure, to the original ground truth ranking benchmark test. We show that also in this case, the conclusions remain unaltered: GeneWalk outperforms the alternative methods (line 256-265, p10).

We thank the reviewer for suggesting these new ground truth analyses. The overall conclusion is that enriched indirectly-related GO terms do not matter for the problem that GeneWalk solves: to rank order the relevance of a gene’s (direct) GO annotations. We have now mentioned this conclusion in our results section (line 263-265, p10).

Thank you for the effort to address this comment. I understand what was done to address this point. However, I am not entirely convinced that this is the right thing to do. Propagating the pvalues from the parents to the children does not make much sense to this reviewer since the children are more specific than the parents. If there is evidence that a process such as ensheathment of neurons is significant, propagating the p-values to the children would make them significant and there is no evidence that axon ensheathment for instance is now implicated in the phenotype as opposed to other type of (other children of) ensheathment of neurons. Also, there is no reason to believe that ALL children are meaningful, which is essentially what propagating the significant p-values downstream does. In essence, such downstream propagation of p-value is NOT expected to improve the results.

The net effect of the “is_a” relationships is that a given gene is associated to all the GO terms from the most specific term it is associated with, all the way up to the root. That means propagating genes from the children to the parents, not the other way around. When each GO term is considered, as in the GO analysis used here, this redundancy can influence the results of the classical approach in a negative way. This is perhaps related to the “moonlighting genes” mentioned earlier in the paper. However, this issue has been addressed in the literature by methods designed to eliminate cross-talk cause by the presence of the same genes in several biological processes or pathways (see Donato et al, Tomoioaga et al below). Also an even more appropriate thing to do is to simply down-weight the genes that are associated with many GO terms/pathways as proposed in Tarca et al. I believe that all these approaches came with associated R packages so I would suggest including one or more of these as comparable methods (Table 1) and include the results obtained with these in the validation.

Analysis and correction of crosstalk effects in pathway analysis.

https://dx.doi.org/10.1101/gr.153551.112

Pathway crosstalk effects: shrinkage and disentanglement using a Bayesian hierarchical model

https://dx.doi.org/10.1007/s12561-016-9160-1

Down-weighting overlapping genes improves gene set analysis.

https://dx.doi.org/10.1186/1471-2105-13-136

*Response: As the reviewer suggests, we now include an eighth comparable method in our benchmarking. We have now benchmarked GeneWalk with PADOG, developed by Tarca et al (reference 15, https://dx.doi.org/10.1186/1471-2105-13-136). GeneWalk again performs better when benchmarking is done on the QKI data. We were unable to apply it to the JQ1 data due to PADOG’s requirement of three biological replicates, a constraint we confirmed with Prof. Adi Tarca.*

*The other studies mentioned by the reviewer do not have R packages available: Donato et al. did not provide any and Tomoiaga et al. provided a broken link in the publication. So we did not include them in our analysis, but we do cite the studies now in the background section (references 25 and 27). We hope the reviewer is satisfied that we have addressed this issue.*

10* There is a need for some figure (modify Fig1A?) indicating what we're getting from INDRA. I know what GO terms look like, but have no experience with INDRA.

Response: We have added a new schematic (Figure 1B) which explains in more detail how the gene network is assembled from the INDRA or Pathway Commons knowledge bases. This new schematic visualizes that INDRA provides reaction statements from automated literature reading and pathway database queries (line 79-82, p3 and line 960-957, p36) on the genes of interest. These reaction statements are then used as edges in the GeneWalk network.

Thank you for your effort to address this. In Figure 1B, last panel, there is a network that involves blue nodes. The blue nodes have been defined above, in 1A, as being genes. However, the text (line 113) states that “we generated specific GWNs … which generally resulted in fewer gene-gene and only gene-GO annotation connections”. This is very confusing. “only gene-GO annotations connections” would mean the figure should only contain edges between blue and red nodes.

Please clarify what kinds of nodes are included in this context specific network at this stage and make Figure 1B consistent with that.

*Response: We have now further clarified the results text (line 119-122, p3) and updated Figure 1A-B to explain the gene network differences between INDRA or PC in more detail. INDRA provides a gene network that can include gene-GO connections represented by INDRA statements, whilst the PC database only contains gene-gene reaction statements. These differences are also mentioned in the Figure 1 legend (line 980-986, p22). To complete the generation of the full GWN (Figure 1A), both INDRA and PC-derived gene networks are then joined with the GO ontology (Figure 1A) through gene-GO annotation connections.*

12* 654 - 752 Editor ◦ though the detail is much appreciated, would these Methods for competing software be better suited as Supplementary Methods?

Response: In line with the suggestion, we have moved the indicated methods of the alternative methods to the Additional file 1: Supplemental Methods section. We also refer to these supplemental methods on line 766-768 (p28), where the comparison analysis is explained in detail.

The changes are satisfactory.

However, in the section on Network representation (lines 116, 127), it is not clear how the network is trained. What is the input and what is the output? Is that an auto-encoder i.e. is the desired output for a given vector the same vector of pairs, just looking for a representation in a space with fewer dimensions? Or is the input vector the set of all inputs from the input-output pairs, and the desired output the set of all outputs from these pairs, looking to learn a model for the associations between inputs and outputs?

*Response: We have further clarified how the NN is trained in the results section: the input and output vectors are one-hot encodings of the GWN nodes (line 137, p4). The GWN node pairs sampled through random walks form input-output pairs to train the NN (line 134-135, p4). So this is not an auto-encoder NN. For completeness, we have now also included in this section a reference to the methods section where all methodological details are included (line 138, p4).*

Reviewer #2:

3. In the methods section, it is indicated that "to correct for multiple GO annotation testing for a given gene, we utilized the Benjamini-Hochberg FDR adjusted p-value". Do I understand correctly that FDR adjustment was done for each individual gene separately? In that case, I believe this procedure is incorrect, and the FDR adjustment should be applied to all the tested gene-GO term associations at the same time (what GeneWalk examines is whether, among all the gene-GO term associations, there are significant associations in a given context).

Response: The reviewer has correctly understood that the FDR adjustment is applied for each gene individually. We believe this method is appropriate because our methodology is gene-specific. So for any given gene of interest that the end user chooses to look at, GeneWalk provides the FDR adjusted p-values of its GO annotations. If the FDR adjustment would be applied over all gene-GO term associations, this would address a different question: given all genes and their functions, what are the most relevant pairs in the context. This would then be a global comparison across all genes and no longer a gene-specific comparison. We hope that these clarifications resolve this concern of the reviewer.

I thank the authors for addressing most of my concerns. However, one critical issue remains (about FDR adjustment) that requires revision of some of the results and figures.

The authors have confirmed that they have performed FDR correction for each gene separately, and have indicated in their response to my previous comment that “If the FDR adjustment would be applied over all gene-GO term associations, this would address a different question: given all genes and their functions, what are the most relevant pairs in the context”. However, this global analysis of all gene-GO term associations is in fact what the authors have done in many places in the manuscript, and therefore adjusting the p-value for each gene has resulted in the incorrect FDR. For example:

- Page 17: “Using INDRA and Pathway Commons as the knowledge bases, we applied GeneWalk to these DE genes and found that 24% had at least one relevant GO term (FDR = 0.05, Additional file 2)”.

- Figure 3F: “Boxplots of the GO term levels of all significant (p-adjust < 0.1) gene-GO annotation pairs across all qki DE genes present in the GWN.”

- Figure 3H: “Hexagon density plot for all genes of interest (N=1861) in terms of number of connected GO terms and number of relevant GO terms (at FDR=0.05) …”.

- Figure 4D: “The number of different unique GO annotations (y-axis) that are significant (p-adjust < 0.1) as a percentage of all unique GO annotation terms across all JQ1 DE genes present in the GWN”.

Please note that the above list is not an exhaustive list of the analyses that rely on this incorrect FDR calculation to look at global patterns across all gene-GO associations. As the authors have mentioned in their comment, the p-value adjustment process that is used in this version of the manuscript is *only* valid if there is a single pre-defined gene that is of interest and the user wants to know what terms are relevant to that one gene. In any other case, the current procedure for FDR calculation is invalid.

I emphasize this issue because not only it affects many of the analyses presented in this paper, but also it is highly prone to misuse and misinterpretation by future users. So, in addition to revising the results in this paper, the authors need to ensure that their package addresses this issue: my recommendation is that if the user has explicitly asked for relevant GO terms of one or a few pre-defined genes, then FDR correction should be done only to the gene-GO pairs involving those genes of interest. Otherwise, a global FDR correction should be provided to the user.

*Response: We agree with the reviewer’s comments. We have now performed a global FDR correction to all our global analyses and we term the resulting globally FDR corrected p-value as “global p-adjust”. To clarify the difference, the gene-specific FDR corrected p-value, previously named mean p-adjust, is now referred to as “gene p-adjust”. This has led us to update all our global analyses results throughout the manuscript and figures:*

*1C*

*3C (%uniq), F, G, H ,I*

*4D (%uniq), G*

*5E*

*S1J, K, L*

*S2A, C, D, F*

*S3A, B, D*

*S4C*

*S5B*

*S6C,D*

*Additional File 2 and 4*

*We conclude that, despite this more stringent global FDR correction, the overall conclusions did not change. Only a few (gene, GO term) pairs are no longer statistically significantly relevant when comparing gene-padj to global-padj at FDR = 0.1 (line 300 p7 ,line 389, p9 and Additional file 2). Nevertheless, we are gratified to know that the method is now more precise and rigorous.*

*Lastly, we have also updated the statistics in our GeneWalk software package to include both the global_padj and gene_padj side-by-side. Because many people might perform global analyses, we have emphasized the global_padj as the first data column that is closest to the gene-GO pair information in the main GeneWalk output file (provided for all contexts in Additional file 2). The gene-padj is located to the right of the global-padj and thus less emphasized for the reader. We now also explain in the manuscript (line 154-156, p4 and line 996-999, p22) and on the Github readme page of the package that the global_padj should be used when GeneWalk is used for global analyses or to identify relevant gene functions by browsing through the GeneWalk output file. The gene-padj is appropriate when the user investigates the function relevance of a pre-defined gene.*
